# Genome-Wide Association Meta-analysis of Neuropathologic Features of Alzheimer's Disease and Related Dementias

**DOI:** 10.1371/journal.pgen.1004606

**Published:** 2014-09-04

**Authors:** Gary W. Beecham, Kara Hamilton, Adam C. Naj, Eden R. Martin, Matt Huentelman, Amanda J. Myers, Jason J. Corneveaux, John Hardy, Jean-Paul Vonsattel, Steven G. Younkin, David A. Bennett, Philip L. De Jager, Eric B. Larson, Paul K. Crane, M. Ilyas Kamboh, Julia K. Kofler, Deborah C. Mash, Linda Duque, John R. Gilbert, Harry Gwirtsman, Joseph D. Buxbaum, Patricia Kramer, Dennis W. Dickson, Lindsay A. Farrer, Matthew P. Frosch, Bernardino Ghetti, Jonathan L. Haines, Bradley T. Hyman, Walter A. Kukull, Richard P. Mayeux, Margaret A. Pericak-Vance, Julie A. Schneider, John Q. Trojanowski, Eric M. Reiman, the Alzheimer's Disease Genetics Consortium (ADGC), Gerard D. Schellenberg, Thomas J. Montine

**Affiliations:** 1 John P. Hussman Institute for Human Genomics, University of Miami, Miami, Florida, United States of America; 2 Division of Epidemiology, University of Pennsylvania, Philadelphia, Pennsylvania, United States of America; 3 Neurogenomics Division, Translational Genomics Research Institute, Phoenix, Arizona, United States of America; 4 Department of Psychiatry & Behavioral Sciences, University of Miami, Miami, Florida, United States of America; 5 Department of Molecular Neuroscience, University College London, London, United Kingdom; 6 New York Brain Bank, Columbia University, New York, New York, United States of America; 7 Department of Neuroscience, Mayo Clinic College of Medicine, Jacksonville, Florida, United States of America; 8 Department of Neurological Sciences, Rush University, Chicago, Illinois, United States of America; 9 Department of Neurology and Psychiatry, Brigham and Women's Hospital, Boston, Harvard Medical School, Boston, Massachusetts, United States of America; 10 Group Health Research Institute, Seattle, Washington, United States of America; 11 Department of Medicine, University of Washington, Seattle, Washington, United States of America; 12 Department of Human Genetics, University of Pittsburgh Graduate School of Public Health, Pittsburgh, Pennsylvania, United States of America; 13 Department of Pathology, University of Pittsburgh School of Medicine, Pittsburgh, Pennsylvania, United States of America; 14 Department of Neurology, University of Miami, Miami, Florida, United States of America; 15 Department of Psychiatry, Vanderbilt University, Nashville, Tennessee, United States of America; 16 Department of Psychiatry, Mount Sinai Hospital, New York, New York, United States of America; 17 Department of Neurology, Oregon Health & Science University, Portland, Oregon, United States of America; 18 Biomedical Genetics, Boston University School of Public Health, Boston, Massachusetts, United States of America; 19 C.S. Kubik Laboratory for Neuropathology, Massachusetts General Hospital, Charlestown, Massachusetts, United States of America; 20 Department of Pathology and Laboratory Medicine, Indiana University, Indianapolis, Indiana, United States of America; 21 Department of Epidemiology and Biostatistics, Case Western Reserve University School of Medicine, Cleveland, Ohio, United States of America; 22 Department of Neurology, Massachusetts General Hospital, Harvard Medical School, Boston, Massachusetts, United States of America; 23 Department of Epidemiology, National Alzheimer's Coordinating Center, University of Washington, Seattle, Washington, United States of America; 24 Department of Neurology, Taub Institute for Research on Alzheimer's Disease and the Aging Brain, Columbia University, New York, New York, United States of America; 25 Department of Pathology (Neuropathology), Rush University Medical Center, Chicago, Illinois, United States of America; 26 Department of Pathology and Laboratory Medicine, University of Pennsylvania, Philadelphia, Pennsylvania, United States of America; 27 Arizona Alzheimer's Consortium, Banner Alzheimer's Institute, Phoenix, Arizona, United States of America; 28 Department of Psychiatry, University of Arizona, Phoenix, Arizona, United States of America; 29 Department of Pathology, University of Washington, Seattle, Washington, United States of America; Georgia Institute of Technology, United States of America

## Abstract

Alzheimer's disease (AD) and related dementias are a major public health challenge and present a therapeutic imperative for which we need additional insight into molecular pathogenesis. We performed a genome-wide association study and analysis of known genetic risk loci for AD dementia using neuropathologic data from 4,914 brain autopsies. Neuropathologic data were used to define clinico-pathologic AD dementia or controls, assess core neuropathologic features of AD (neuritic plaques, NPs; neurofibrillary tangles, NFTs), and evaluate commonly co-morbid neuropathologic changes: cerebral amyloid angiopathy (CAA), Lewy body disease (LBD), hippocampal sclerosis of the elderly (HS), and vascular brain injury (VBI). Genome-wide significance was observed for clinico-pathologic AD dementia, NPs, NFTs, CAA, and LBD with a number of variants in and around the apolipoprotein E gene (*APOE*). GalNAc transferase 7 (*GALNT7*), ATP-Binding Cassette, Sub-Family G (WHITE), Member 1 (*ABCG1*), and an intergenic region on chromosome 9 were associated with NP score; and Potassium Large Conductance Calcium-Activated Channel, Subfamily M, Beta Member 2 (*KCNMB2*) was strongly associated with HS. Twelve of the 21 non-*APOE* genetic risk loci for clinically-defined AD dementia were confirmed in our clinico-pathologic sample: *CR1*, *BIN1*, *CLU*, *MS4A6A*, *PICALM*, *ABCA7*, *CD33*, *PTK2B*, *SORL1*, *MEF2C*, *ZCWPW1*, and *CASS4* with 9 of these 12 loci showing larger odds ratio in the clinico-pathologic sample. Correlation of effect sizes for risk of AD dementia with effect size for NFTs or NPs showed positive correlation, while those for risk of VBI showed a moderate negative correlation. The other co-morbid neuropathologic features showed only nominal association with the known AD loci. Our results discovered new genetic associations with specific neuropathologic features and aligned known genetic risk for AD dementia with specific neuropathologic changes in the largest brain autopsy study of AD and related dementias.

## Author Summary

Alzheimer's disease (AD) and related dementias are a major public health challenge and present a therapeutic imperative for which we need additional insight into molecular pathogenesis. We performed a genome-wide association study (GWAS), as well as an analysis of known genetic risk loci for AD dementia, using data from 4,914 brain autopsies. Genome-wide significance was observed for 7 genes and pathologic features of AD and related diseases. Twelve of the 22 genetic risk loci for clinically-defined AD dementia were confirmed in our pathologic sample. Correlation of effect sizes for risk of AD dementia with effect size for hallmark pathologic features of AD were strongly positive and linear. Our study discovered new genetic associations with specific pathologic features and aligned known genetic risk for AD dementia with specific pathologic changes in a large brain autopsy study of AD and related dementias.

## Introduction

Studies of brain aging with brain autopsy endpoint have repeatedly demonstrated that dementia in older individuals most often derives from three common diseases: Alzheimer's disease (AD), vascular brain injury (VBI) especially small vessel disease, and Lewy body (LB) disease (LBD) [1]. Importantly, while each of these diseases exclusively can cause dementia, they commonly co-exist in older patients with dementia [2]. These same brain autopsy studies also commonly observed pathologic changes of AD, LBD, and/or VBI in older individuals carefully demonstrated to be cognitively normal proximate to death. Indeed, it was these observations from cognitively intact “controls” that first led to the hypothesis of cognitive reserve, the idea of excess functional capacity that can mask clinical expression of disease. Biomarker and molecular neuroimaging studies also have observed abnormal changes in cognitively normal individuals that typically occur in patients with dementia, and have demonstrated with longitudinal observation that some of these changes are pathologic [3]. Thus, dementia in older individuals is a syndrome that derives most commonly from the idiosyncratic convergence of three chronic disease processes, AD, VBI, and/or LBD, that each appear to have prevalent latency prior to clinical expression.

The suffering to patients and loved ones and cost to health care systems from the global burden of dementia are staggering, and projected to increase markedly in the coming 20 years [4]. While interventions exist to mitigate some types of VBI, the major unmet medical needs are disease-modifying therapies for AD and LBD. Many efforts are underway to achieve this therapeutic imperative, although none yet has met with reproducible success in clinical trials. One response to these setbacks is to revise the approach to existing therapeutic targets, such as using existing experimental interventions in earlier stages of disease or in selected subgroups. Another approach is to increase the repertoire of therapeutic targets by expanding our knowledge of the molecular drivers of disease; this rationale animates the several recent genome-wide association studies (GWAS) for AD [5]–[11].

Given the pathologic complexity of AD and related dementias, and the prevalence of latent disease, we hypothesized that direct analysis of neuropathologic features might align known genetic risk loci with specific diseases processes as well as identify novel genetic variants associated with specific neuropathologic features. To test these hypotheses the Alzheimer's Disease Genetics Consortium (ADGC) assembled a set of 4,914 samples with genome-wide genotyping data and neuropathologic data, and performed genome-wide association tests of AD and related diseases. We performed both genome wide association and analyses focused on known AD dementia genetic risk loci while using three approaches to the neuropathologic data: a clinico-pathologic definition of AD dementia or controls, focus on the core neuropathologic features of AD, i.e., neurofibrillary tangles (NFTs) and neuritic plaques (NPs), in cases and controls combined, and inclusion of commonly co-morbid neuropathologic features observed in older individuals with dementia, i.e., cerebral amyloid angiopathy (CAA), LBD, hippocampal sclerosis in the elderly (HS), and VBI.

## Results

### Genome-wide association

First we performed a GWAS of clinico-pathologic AD dementia, that is, cases with clinical dementia confirmed to have moderate to high levels of core AD pathologic changes and controls without dementia and no or low levels of core AD pathologic changes (**Table 1**). Results and Quantile-Quantile plots are detailed in the supporting material (**Tables S1, S2; Figures S1, S2**). A number of variants in and around the apolipoprotein E gene (*APOE*) achieved genome-wide significance (rs6857, p-value = 2×10^−62^). One additional variant in the PHD Finger Protein 21B gene (*PHF21B*) achieved genome-wide significance (chr22:45354131, p-value = 1.9×10^−8^). However, the variant was only imputed in one dataset (albeit the largest set, ADC), had a low minor allele frequency (MAF) ( = 0.017), had a modest imputation info score ( = 0.428), and was not well-supported by association at other variants in the region (**Figure S3**). These are typically the signs of a false positive, but we do note that a recent meta-analysis of primarily clinic-based samples by the International Genomics of Alzheimer's Project (IGAP) [11] found nominal association at this locus (p-value = 0.00659), with the same direction of effect as we report. There is however some sample overlap between this and the Lambert et al study.

**Table 1:** Case-control criteria.

Clinical^a^	Pathologic			Phenotype Decision
	*NIA/Reagan* b	*NP score* c	*NFT Braak stage* d	
Dementia Group	intermediate or high likelihood	—	—	Neuropathologic AD
	—	moderate or frequent	III–VI	
Not Demented Group	low likelihood	—	—	Neuropathologic Control
	—	none or sparse	0, I, or II	
	—	none	III or IV	

Neuropathologically-confirmed AD and control criteria. Abbreviations: NP: neuritic plaque; NFT: neurofibrillary tangles. AD: Alzheimer's disease.

a Dementia Group met DSM-IV criteria or had a clinical dementia rating greater than zero. Not Demented Group did not meet DSM-IV criteria for dementia, had no mild cognitive impairment and—when available—a clinical dementia rating of zero.

b Hyman and Trojanowski, 1997 [23].

c Mirra et al., 1993 [21].

d Braak and Braak, 1991 [22].

**Figure 1:**
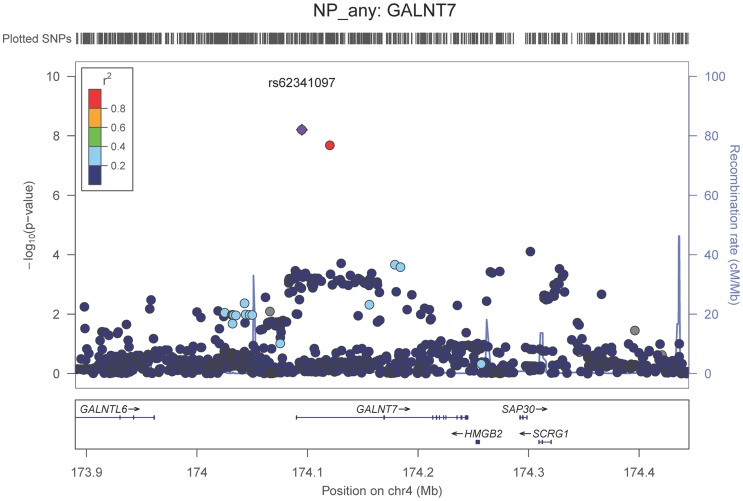
Regional association plot for *GALNT7* and the neuritic plaque (any vs. none) analysis. The purple dot indicates the most associated SNP in the region. The x-axis is basepair position, and y-axis is the −log(p-value), base 10.

**Figure 2:**
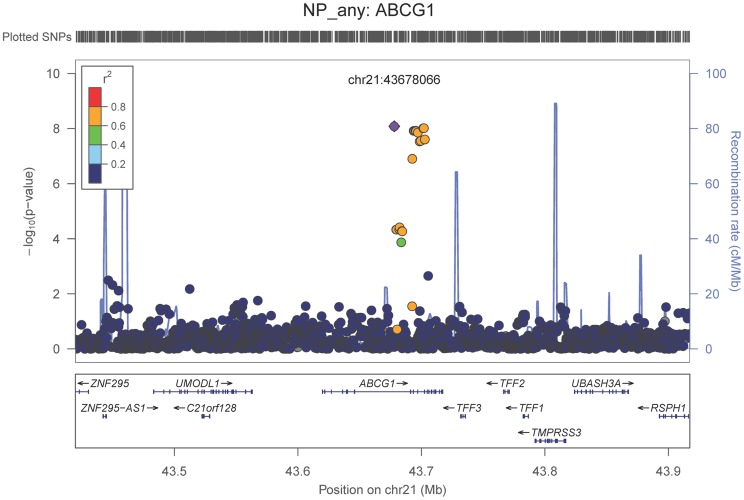
Regional association plot for *ABCG1* and the neuritic plaque (any vs. none) analysis. The purple dot indicates the most associated SNP in the region. The x-axis is basepair position, and y-axis is the −log(p-value), base 10.

**Figure 3:**
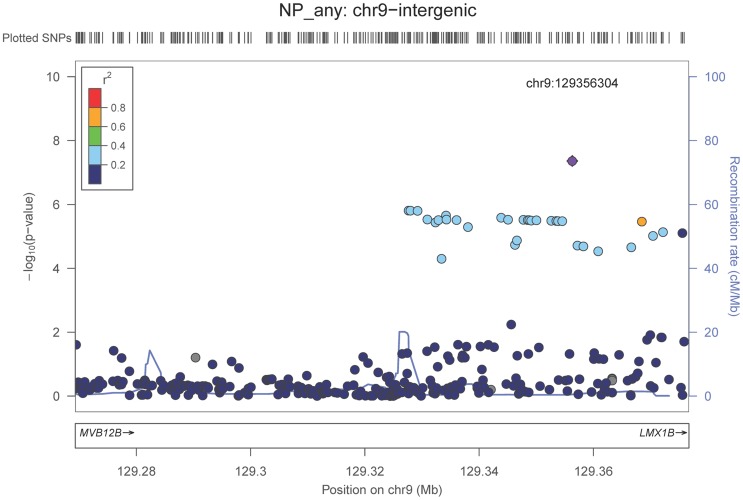
Regional association plot for the chromosome 9:129,280,000–129,380,000 region and the neuritic plaque (any vs. none) analysis. The purple dot indicates the most associated SNP in the region. The x-axis is basepair position, and y-axis is the −log(p-value), base 10.

We also performed a GWAS of core AD pathologic changes; we performed two ordinal analyses for NFT and an “any vs. none” NP analysis (presence of NPs as one category and absence of NPs as another; see Methods). We found variants in the *APOE* region to be highly associated with both NPs (p-value<10^−46^ any-none; p-value<10^−26^ ordinal) and NFTs (p-value<10^−46^ ordinal with seven categories; p-value<10^−43^ ordinal with four categories) (**Tables S3, S4, S5, S6, Figures S4, S5, S6, S7**). Three additional loci were significantly associated with NPs in the “any vs. none” analysis: GalNAc transferase 7 (*GALNT7*; minimum p-value = 6.0×10^−9^; **Figure 1**), ATP-Binding Cassette, Sub-Family G (WHITE), Member 1 (*ABCG1*; minimum p-value = 8.0×10^−9^; **Figure 2**), and an intergenic region on chromosome 9 (minimum p-value = 4.3×10^−8^; **Figure 3**). Each of these three loci were tested in multiple datasets, and there was no evidence of heterogeneity of effect size (heterogeneity p-value>0.5 for each; **Figures S8, S9, S10**). These three additional loci were not significantly associated in the recent IGAP analysis (p-value>0.05). There were no additional genome-wide significant loci with the NP ordinal analysis or the NFT analyses (**Tables S3, S4, S5, S6**).

**Figure 4:**
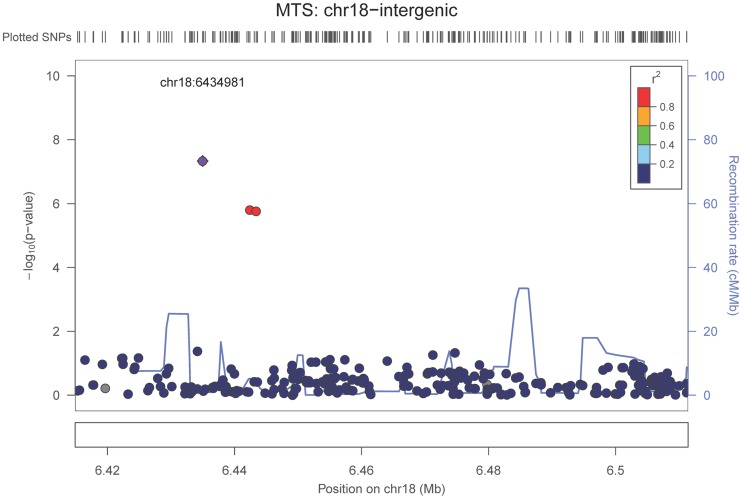
Regional association plot for the chromosome 18:6,420,000–6,520,000 region and the hippocampal sclerosis (any vs. none) analysis. The purple dot indicates the most associated SNP in the region. The x-axis is basepair position and y-axis is the −log(p-value), base 10.

**Figure 5:**
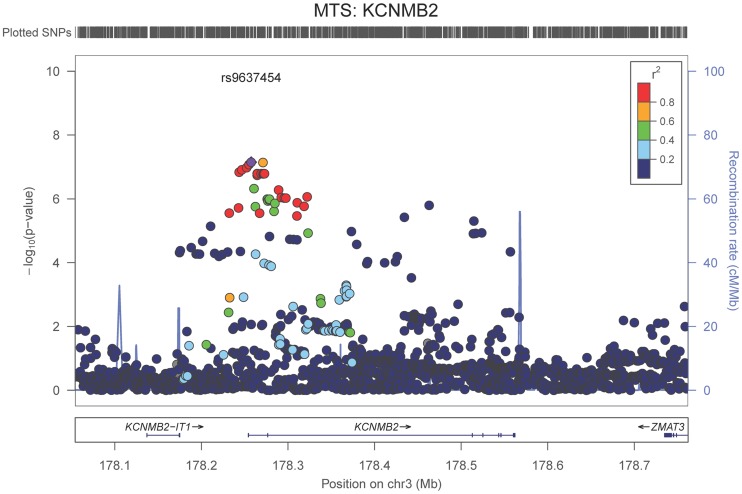
Regional association plot for *KCNMB2* and the hippocampal sclerosis (any vs. none) analysis. The purple dot indicates the most associated SNP in the region. The x-axis is basepair position, and y-axis is the −log(p-value), base 10.

Our last GWAS was of co-morbid neuropathologic features. *APOE* showed significant genome wide association for both CAA (minimum p-value = 2.8×10^−23^), and LBD (minimum p-value<1.1×10^−12^) but was not strongly associated with VBI or with HS (**Tables S7, S8, S9, S10, S11, S12, S13; Figures S11, S12, S13, S14, S15, S16, S17**). HS had significant genome-wide association with an intergenic region on chromosome 18 (minimum p-value = 4.6×10^−8^; **Figure 4**) and strong association at the Potassium Large Conductance Calcium-Activated Channel, Subfamily M, Beta Member 2 gene (*KCNMB2*) on chromosome 3 (minimum p-value = 7.1×10^−8^; **Figure 5**). The chromosome 18 locus was only tested in one dataset – the largest set, ADC, while the KCNMB2 locus was tested in multiple datasets and showed no evidence of heterogeneity of effect size (heterogeneity p-value>0.5; **Figure S18**). The chromosome 18 locus did show suggestive association with AD risk in the IGAP analysis (p-value = 0.0611) with an effect size in the same direction reported here. No other significant genome-wide association was discovered for CAA, LBD, or VBI (**Table 2**).

**Table 2:** Genome-wide significant results.

Phenotype	Marker	Chr	Position	EA	RA	Freq	minFreq	maxFreq	Effect	StdErr	Pval	Direction	Gene	Het Pval
Primary (Case-Control)	rs6857	19	45,392,254	T	C	0.29	0.15	0.39	1.614	0.097	2.02E-62	++++++?−	APOE region	0.407
Primary (Case-Control)	22-45354131	22	45,354,131	A	G	0.02	0.02	0.02	−2.408	0.429	1.90E-08	?−??????	PHF21B	.
Complete (Case-Control)	rs6857	19	45,392,254	T	C	0.29	0.15	0.38	1.501	0.116	3.48E-38	++++++−	APOE region	0.486
Complete (Case-Control)	22-45354131	22	45,354,131	A	G	0.02	0.02	0.02	−2.407	0.429	2.00E-08	?−?????	PHF21B	.
NFT Braak (Ordinal I)	rs6857	19	45,392,254	T	C	0.33	0.15	0.39	0.663	0.046	4.73E-47	+++++++?−+	APOE region	<0.0001
NFT Braak (Ordinal II)	rs6857	19	45,392,254	T	C	0.32	0.15	0.39	0.787	0.057	4.83E-44	+++++++?−++	APOE region	0.015
Neuritic Plaque (Ordinal)	rs6857	19	45,392,254	T	C	0.32	0.15	0.40	0.947	0.066	3.14E-47	+++++++?	APOE region	0.207
Neuritic Plaque (Case-Control)	rs6857	19	45,392,254	T	C	0.27	0.15	0.36	1.293	0.119	1.78E-27	++++++?−	APOE region	0.278
Neuritic Plaque (Case-Control)	rs62341097	4	174,094,940	A	G	0.05	0.02	0.06	−1.147	0.198	6.00E-09	−−−−−−−+	GALNT7	0.995
Neuritic Plaque (Case-Control)	21-43678066	21	43,678,066	T	C	0.01	0.01	0.01	−2.108	0.366	8.00E-09	−−??????	ABCG1	0.999
Neuritic Plaque (Case-Control)	9-129356304	9	129,356,304	T	G	0.02	0.01	0.02	−1.766	0.322	4.30E-08	?−−?−−??	none	0.945
Lewy Body (Ordinal I)	rs429358	19	45,411,941	C	T	0.73	0.65	0.87	−0.500	0.070	1.10E-12	−−−−−−	APOE region	0.816
Lewy Body (Case-Control)	rs429358	19	45,411,941	C	T	0.73	0.65	0.87	−0.491	0.074	2.83E-11	−−−−−−	APOE region	0.839
Lewy Body (Ordinal II)	rs429358	19	45,411,941	C	T	0.73	0.65	0.87	−0.508	0.074	4.87E-12	−−−−−−	APOE region	0.815
MTS (Case-Control)	18-6434981	18	6,434,981	A	C	0.01	0.01	0.01	2.560	0.468	4.60E-08	?+??	none	.
CAA (Case-Control)	rs6857	19	45,392,254	T	C	0.34	0.15	0.40	0.671	0.071	2.92E-21	++++?+	APOE region	0.755

Legend:

EA: Effect Allele.

RA: Reference Allele.

Freq: frequency of the effect allele.

min/max Freq: minimum and maximum frequency across the differing cohorts.

Effect: the beta coefficient effect of the non-reference allele.

Het Pval: P-value of the heterogeneity test.

### Analysis of known AD dementia genetic risk loci

**Figure 6:**
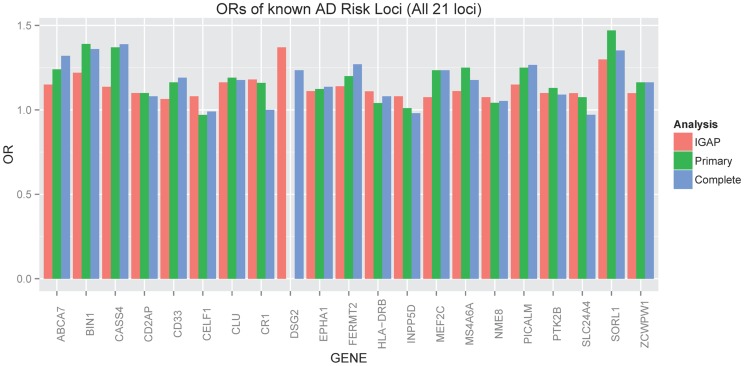
Barplot of OR for known AD risk loci (all 21 loci). Red indicates the estimated OR previously reported through the IGAP consortium [11], green indicates the estimated OR from our primary clinico-neuropathologic case-control analysis, and blue indicates the estimated OR from a more restrictive analysis that required complete documentation of all relevant neuropathologic phenotypes.

The ADGC, together with international collaborators under the banner of IGAP (International Genomics of Alzheimer's Project) has to-date identified 21 common genetic loci associated with AD dementia using primarily clinically ascertained datasets [7], [8], [10], [11], in addition to confirming the *APOE* locus [12]. One of our goals in the study presented here was to test the hypothesis that pathologic confirmation of dementia or control status might reduce phenotypic heterogeneity and thereby enhance known genetic associations with AD dementia. We used two sets that met our clinico-pathologic criteria for case or control (**Table 1**): the “primary” dataset had 3887 cases and 1027 controls which allowed some incomplete documentation of pathologic features, and the “complete” dataset which had 3044 case and 658 controls with more stringent documentation standards. The primary clinico-pathologic analysis confirmed (p-value<0.05) association with 12 of the 21 previously identified [11] non-*APOE* loci: *CR1*, *BIN1*, *CLU*, *MS4A6A*, *PICALM*, *ABCA7*, *CD33*, *PTK2B*, *SORL1*, *MEF2C*, *ZCWPW1*, and *CASS4*; 9 of these also were confirmed in the “complete” analysis (**Table 3**). All loci but one (*CELF1*) had a consistent direction of effect with the odds ratio (OR) previously reported (**Figure 6**). Nine of the twelve loci confirmed in the clinico-pathologic datasets had stronger OR with AD dementia than was observed previously. This is more than would be expected at random (paired t-test p-value = 0.00029 among confirmed loci; p-value = 0.033 among all 21 non-*APOE* loci); OR for *CLU* and *PTK2B* were essentially unchanged from the previous report, and the OR for *CR1* was reduced in our study. These results are consistent with less heterogeneity in our clinico-pathologic sample compared to studies with purely clinical ascertainment schemes. Rare variation in the recently discovered TREM2 and PLD3 genes [13], [14] was not imputed on our reference panel and as such was not assessed in this study. Some nominal association (p-value<0.05) was noted in these genes (*e.g.*, with PLD3 and the MTS phenotype, marker 19-40373284 had a p-value = 0.006118; with TREM2 and the four category Braak phenotype, marker rs17328707 had a p-value = 0.001132, etc). However, given that over 240 markers were tested in each gene, even these minimum p-values would not withstand a gene-based multiple testing correction.

**Table 3:** Clinico-pathologic analysis of IGAP loci.

						IGAP Paper*			Primary Analysis			Complete Analysis		
Type	SNP	GENE	CHR	POS	Major/Minor	MAF	OR	p-value	MAF	OR	p-value	MAF	OR	p-value
Known	rs6656401	CR1	1	207,692,049	G/A	0.197	1.18	5.7E-24	0.197	1.16	**4.00E-02**	0.197	1.00	9.80E-01
Known	rs6733839	BIN1	2	127,892,810	C/T	0.409	1.22	6.9E-44	0.430	**1.39**	**3.40E-06**	0.431	**1.36**	**2.50E-04**
Known	rs10948363	CD2AP	6	47,487,762	A/G	0.266	1.10	5.2E-11	0.278	1.10	1.60E-01	0.274	1.08	3.80E-01
Known	rs11771145	EPHA1	7	143,110,762	G/A	0.338	0.90	1.1E-13	0.334	**0.89**	7.30E-02	0.338	**0.88**	9.10E-02
Known	rs9331896	CLU	8	27,467,686	T/C	0.379	0.86	2.8E-25	0.376	**0.84**	**4.70E-03**	0.370	**0.85**	**4.20E-02**
Known	rs983392	MS4A6A	11	59,923,508	A/G	0.403	0.90	6.1E-16	0.398	**0.80**	**1.30E-04**	0.396	**0.85**	**3.60E-02**
Known	rs10792832	PICALM	11	85,867,875	G/A	0.358	0.87	9.3E-26	0.351	**0.80**	**1.30E-04**	0.351	**0.79**	**1.70E-03**
Known	rs4147929	ABCA7	19	1,063,443	G/A	0.190	1.15	1.1E-15	0.188	**1.24**	**1.40E-02**	0.186	**1.32**	**1.00E-02**
Known	rs3865444	CD33	19	51,727,962	C/A	0.307	0.94	3.0E-06	0.297	**0.86**	**1.60E-02**	0.296	**0.84**	**2.60E-02**
New D	rs9271192	HLA-DRB5/HLA-DRB	6	32,578,530	A/C	0.276	1.11	2.9E-12	0.286	1.04	5.90E-01	0.283	1.08	3.40E-01
New D	rs28834970	PTK2B	8	27,195,121	T/C	0.366	1.10	7.4E-14	0.383	**1.13**	**4.60E-02**	0.381	1.09	2.30E-01
New D	rs11218343	SORL1	11	121,435,587	T/C	0.039	0.77	9.7E-15	0.040	**0.68**	**4.30E-03**	0.039	**0.74**	1.00E-01
New D	rs10498633	SLC24A4/RIN3	14	92,926,952	G/T	0.217	0.91	5.5E-09	0.216	0.93	3.10E-01	0.218	1.03	7.20E-01
New D	rs8093731	DSG2	18	29,088,958	C/T	0.017	0.73	1.0E-04	.	.	.	0.014	0.81	8.40E-01
New D&R	rs35349669	INPP5D	2	234,068,476	C/T	0.488	1.08	3.2E-08	0.485	1.01	8.40E-01	0.490	0.98	8.40E-01
New D&R	rs190982	MEF2C	5	88,223,420	A/G	0.408	0.93	3.2E-08	0.412	**0.81**	**1.10E-03**	0.406	**0.81**	**6.60E-03**
New D&R	rs2718058	NME8	7	37,841,534	A/G	0.373	0.93	4.8E-09	0.359	0.96	5.50E-01	0.354	0.95	4.70E-01
New D&R	rs1476679	ZCWPW1	7	100,004,446	T/C	0.287	0.91	5.6E-10	0.291	**0.86**	**1.80E-02**	0.285	**0.86**	**6.10E-02**
New D&R	rs10838725	CELF1	11	47,557,871	T/C	0.316	1.08	1.1E-08	0.308	0.97	6.20E-01	0.308	0.99	8.80E-01
New D&R	rs17125944	FERMT2	14	53,400,629	T/C	0.092	1.14	7.9E-09	0.093	**1.20**	8.50E-02	0.092	**1.27**	6.90E-02
New D&R	rs7274581	CASS4	20	55,018,260	T/C	0.083	0.88	2.5E-08	0.073	**0.73**	**3.30E-03**	0.076	**0.72**	**1.10E-02**

*Lambert et al., 2013 [11].

IGAP: International Genomics of Alzheimer's Project.

Bolded OR indicates an OR more extreme than that reported by the IGAP paper [11].

Bolded p-value indicates a p-value significant at the alpha = 0.05 level, uncorrected.

**Figure 7:**
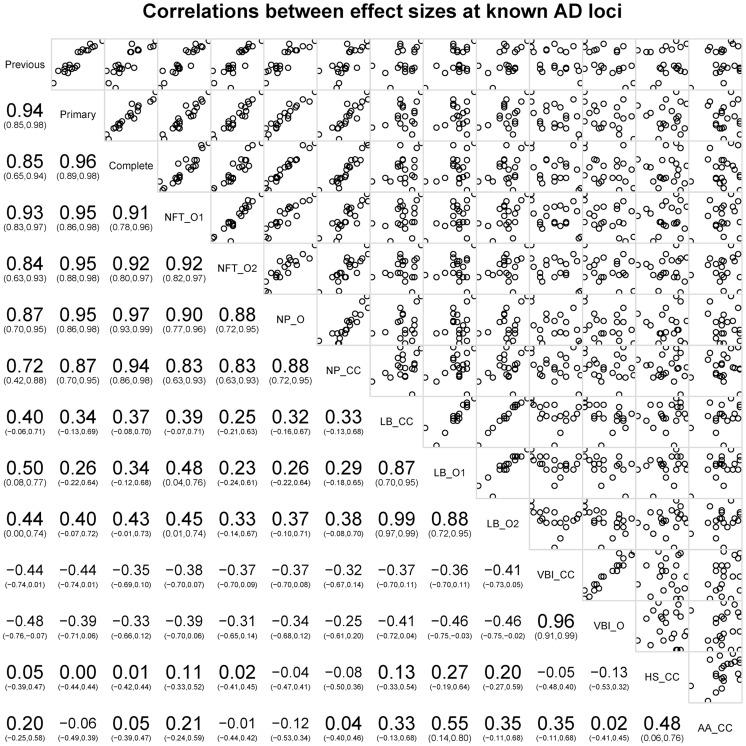
Correlations of OR for known AD risk loci and the neuropathology phenotypes. Bottom left indicates the magnitude, direction, and confidence interval of the correlation. Top right shows plots of the data points against each other. The diagonal box indicates the phenotypes; off-diagonal boxes indicate the correlations of the OR for the corresponding phenotypes.

Next we tested the hypothesis that the 21 common AD loci are varyingly associated with the core AD neuropathologic features among all subjects, combining cases and controls (n = 4,914). The effect sizes for 12 of the 21 loci (11 of the 12 confirmed loci) were significantly associated with one or both of the core neuropathologic features of AD with a consistent direction of effect (****). Though *CR1* is associated with clinico-pathologic AD, we do not confirm the previously reported associations with NPs and CAA [15]; we do confirm the previous associations with *CD2AP* and *ABCA7* and NPs [15]. To gain additional insight into the potential molecular drivers of specific neuropathologic features, we compared the effect sizes for risk of AD dementia (function) with the effect sizes for the two core AD neuropathologic features (structure) across these 21 loci in all subjects. Effect size estimates for both NFTs and NPs were strongly positively correlated to the previously reported effect sizes (p-value<10^−6^ for the NFT ordinal traits; p-value<10^−6^ for the NP ordinal analysis; p-value<10^−4^ for NP “any vs. none” analysis (**Figure 7** **; Figure S19**).

The co-incident neuropathologic features showed only nominal association with the known AD loci (**Table S15**). LBD (n = 3526) was nominally associated with *MEF2C* with effect sizes similar to both NFTs and NPs. LBD was also nominally associated with *SORL1*, with an effect size direction opposite that previously reported for AD dementia. HS (n = 2866) was nominally associated with *PTK2B*; VBI (n = 2940) showed nominal association at *NME8*; CAA (n = 2807) showed no association with any of the previously reported loci. We next compared the co-morbid neuropathologic features effect size estimates to the known AD risk effects (Figure 7). While the core AD feature effect sizes showed strong, positive correlations with the previously reported effect sizes, the picture at co-morbid features was more complicated. LBD effect sizes showed weak moderate positive correlations with the previously reported effect sizes (**Figure S20**; slope p-value = 0.035), while VBI showed a moderate negative correlation with the previously reported AD dementia effect sizes (**Figure S21**; slope p-value = 0.0292). To test if these correlations had to do with the study ascertainment scheme, we repeated the analysis but using cases only; in the case-only set LBD effect sizes were no longer correlated with the previously reported effect sizes (p-value = 0.86), while the correlation of the VBI effect sizes with those previously reported was stronger (p-value = 4.22×10^−4^) (**Figures S20, S21**).

Finally, we compared NP effect sizes against the effect sizes for NFTs and CAA at each of these loci; we observed a strong, positive, and linear association between effect sizes of NPs and NFTs, but no correlation between NPs and CAA (**Figure 7**).

## Discussion

Cognitive impairment among older individuals is a complex convergent trait that often occurs with mixed pathology: AD, LBD, and VBI, which each have prevalent prodromal and latent stages in addition to full clinical expression as dementia. Indeed, approximately 95% of subjects without dementia in this age group have some pathologic evidence of at least one of these three diseases. These facts as first discovered in neuropathologic studies of brain aging and subsequently validated in part by neuroimaging and biomarker studies present serious challenges to diagnose accurately and comprehensively the diseases that cause an individual's dementia based exclusively on clinical data, and clearly demonstrate that age-matched control populations are variably, and potentially extensively, “contaminated” with latent disease. Virtually all previous GWAS of AD have been based on cases and controls defined exclusively by clinical criteria. To address this limitation, we undertook a GWAS of dementia that focused on neuropathologic data (i) to test the hypothesis that clinico-pathologic characterization of cases and controls could be used to strengthen previous genetic associations with AD made by clinical criteria, (ii) to test the hypothesis that genetic associations for AD dementia will selectively align with specific neuropathologic features, and (iii) to discover new genetic associations with neuropathologic features of AD and related dementias.

Although we assembled a large brain autopsy cohort, it is still a relatively modest number of samples for GWAS compared to the larger IGAP GWAS where subjects were primarily clinically diagnosed cases and controls. The reduction in statistical power due to a smaller sample must be balanced against improved phenotypic homogeneity. With this balance in mind, our study confirmed 12 of the 21 previously reported loci as significantly associated with AD dementia. Our association with clinico-pathologic AD dementia was strengthened for 9 of these 12 loci (*ABCA7*, *BIN1*, *CASS4*, *CD33*, *MEF2C*, *MS4A6A*, *PICALM*, *SORL1*, and *ZCWPW1*), essentially unchanged for *CLU* and *PYK2B*, and diminished for *CR1* to unity with increasingly stringent pathologic criteria. *CR1* also is the only locus that we confirmed as associated with AD dementia that was not significantly associated with a specific neuropathologic feature. One possible explanation is that previous associations of *CR1* with AD dementia may be related more to clinical expression of disease rather than to the accumulation of NPs and NFTs; it is of note though that *CR1* has been associated with NPs and CAA previously [15]. The same may be true for the other 9 loci that were not confirmed in our clinico-pathologic case-control analysis; however, lack of confirmation also may be the result of limitations of smaller sample size outweighing the potential advantages of reduced phenotypic heterogeneity or perhaps even spurious associations in previous studies.

Clinical expression of dementia encompasses at least several processes that include disease mechanisms that produce stress and injury, response to injury, physiologic compensation, and cognitive reserve. Our second major goal was to determine the alignment between AD dementia genetic risk loci and specific neuropathologic features that are core to (NPs and NFTs) or co-morbid with (LBD, VBI, HS, CAA) AD in an attempt to clarify the relevant molecular mechanisms that underlie these characteristic lesions that are related to injury and response to injury. Association tests for NFTs *and* NPs were significant for five of the previously reported loci that were validated in our clinico-pathologic GWAS (*ABCA7*, *BIN1*, *CASS4*, *MEF2C*, and *PICALM*); NFTs were specifically associated with *CLU*, *SORL1*, and *ZCWPW1*, and NPs were specifically associated with *MS4A6A* and *CD33*. In all instances the effect sizes for NPs or NFTs were in the same direction as the previously reported effects for AD dementia. Effects sizes for LBD were increased with *SORL1* and decreased with *MEF2C*, and HS was associated with *PTK2B*; no significant association was observed between previously reported AD dementia loci and VBI or CAA. Association of LBD with *SORL1* locus is novel and, interestingly, the effect size was in the direction opposite to its association with NFTs. The *SORL1* locus has not been identified in previous studies of Parkinson's disease (PD), another LBD, but these were investigations of PD defined by its movement disorder rather than LBD in the context of AD dementia as we have done here; this unexpected association will require replication and further investigation. We analyzed NPs and NFTs as binary (any *vs.* none) and finer graded (ordinal) phenotypes; stronger associations were observed with binary analyses perhaps because of variation in assigning histopathologic stages to these characteristic AD lesions.

We observed a linear correlation between the previously reported AD dementia effect sizes and the effect sizes for NPs and NFTs determined in our cohort whether we included all 21 IGAP loci or limited our analysis to the 12 loci confirmed in our clinico-pathologic investigation. We do not think this is a foregone conclusion. Indeed, the literature is replete with commentary that one or both of the core AD lesions are a product of brain aging rather than AD. Our results strongly support the view that the molecular drivers underlying the accumulation of NPs and NFTs in brain are largely shared with the molecular drivers of severely impaired cognitive function. Interestingly, there was not a significant correlation with CAA, suggesting that this other form of amyloidosis has a different genetic risk profile than NP accumulation. Using the same approach, correlation of effect sizes for IGAP loci with effect sizes for LBD was at best weakly positive, and for VBI was negative. This last observation was unexpected and, if validated, might indicate that the genetic risk architecture for AD and VBI are inversely related, an intriguing possibility that stimulates speculation over why this may be. It also suggests a possible approach to genetic stratification of risk for two of the most common causes of cognitive impairment and dementia in older individuals.

Our third goal was genome-wide discovery of associations with the six neuropathologic features to gain additional insights into the molecular drivers of these characteristic lesions. As expected, there was a very strong association of the *APOE* locus with our clinico-pathologically defined AD dementia. We observed a strong association between the *APOE* locus and NFTs, NPs, CAA, and LBD, but not with HS or VBI. While the association of *APOE* with both core features and a secondary neuropathologic feature of AD is expected from ascertainment bias, the association with LBD may not be so simple. Indeed, an association between *APOE* and Dementia with Lewy bodies (DLB) has been varyingly supported or refuted in several smaller studies that did not include pathologic evaluation to exclude contribution from unknown and likely varying amounts of AD. However, in a recent large study of DLB and PD dementia that included pathologic evaluation, we demonstrated that inheritance of *APOE ε4* allele is a significant risk factor for DLB even in the absence of AD [16]. In addition, our GWAS showed a genome-wide significant association between NPs and *ABCG1*, a locus previously implicated in AD [17]. The association of NPs with *GALNT7* is novel and potentially reinforces existing genetic associations with other genes that encode for members of this family of transferases. Indeed, alteration in the O-glycosylation pattern of amyloid beta peptides and NPs has been reported in AD [18]–[20]. Finally, we made a novel association of HS with a variant of *KCMNB2*, which encodes a subunit of a Ca^++^-gated K^+^ channel that is key to neuronal excitability and is highly expressed by hippocampal pyramidal neurons in sector CA1. Although this discovery requires validation, it may have significance beyond AD dementia since HS also occurs frequently in some forms of fronto-temporal dementia. While additional replication datasets are ideal for these novel loci, we do note that the meta-analysis approach here is a type of replication, and that all of the novel variants with support from multiple datasets had no signs of heterogeneity of effect size.

In summary, this genetic association study of autopsy brain enhanced some and diminished other known genetic risk loci for AD dementia, highlighted a subset of the loci previously associated with AD dementia as potential molecular drivers of specific neuropathologic features of AD, and discovered new genetic loci associated with specific neuropathologic features. These novel results provide new insights into new candidate therapeutic targets for AD and related dementias that will require validation and functional investigation.

## Methods

### Sample selection

Samples were contributed by the National Institute on Aging (NIA) Alzheimer's Disease Centers (ADCs) and ADGC-collaborating studies. The ADC dataset includes samples ascertained and evaluated by the Clinical and Neuropathology Cores of the 29 NIA-funded ADCs. The National Alzheimer's Coordinating Center (NACC) organizes a collection of phenotype data from the ADCs, organizes its database, coordinates implementation of definitions of AD cases and controls, and oversees collection and distribution of samples.

The ADGC collaborating studies include the Adult Changes in Thought study (ACT; Eric B. Larson, PI), the Mayo Clinic Alzheimer's Disease Research Center (MAYO; Steven G. Younkin, PI), the University of Miami Brain Endowment Bank (MBB; Deborah Mash, PI), the National Institute on Aging Late-Onset Alzheimer's Disease Family Study (NIA-LOAD; Richard Mayeux, PI), the Oregon Health & Science University Alzheimer's Disease Center (OHSU; Patricia Kramer, PI), the Religious Orders Study and Memory and Aging Project (ROSMAP; David Bennett, PI), the Translational Genomics Research Institute (TGEN; Eric Reiman, PI), the University of Pittsburgh Alzheimer's Disease Research Center (UP; Kamboh, PI), the University of Miami Hussman Institute for Human Genomics (UM; Pericak-Vance, PI), the Vanderbilt University Center for Human Genetics Research (VU; Jonathan Haines, PI), and the Mount Sinai School of Medicine (MSSM; Joseph Buxbaum PI) (see **Table S16** for details; descriptive statistics found in **Table S17**).

All samples had thorough neuropathologic examination according to consensus criteria that existed at the time that our study was initiated. Prior to analysis, neuropathologic data were reviewed and harmonized by a single neuropathologist (TJM) to ensure consistency across assessment sites.

### Neuropathology methods and eligibility criteria

Samples were evaluated at each site. Assessment of NPs and NFTs followed the protocols of CERAD [21], and Braak and Braak [22], respectively, at all sites. Criteria for assessment of co-morbid features were reviewed with each site and only those using comparable methods, i.e., immunohistochemistry for alpha-synuclein to detect LBs, and similar sampling protocols were included in our analyses. For inclusion as a case, the subject must have been diagnosed with dementia (DSM-IV criteria or clinical dementia rating greater than zero) and the subject's autopsy must have either NIA/Reagan classification of intermediate or high likelihood of AD [23], or have an NP score of moderate/frequent and NFT Braak stage of III–VI. For inclusion as a neuropathologically-confirmed control, each subject must not have had a clinical diagnosis of dementia or MCI within two years of death (does not meet DSM-IV criteria for dementia, no mild cognitive impairment, and—when available—clinical dementia rating of zero), no diagnosis of other neurologic disease by clinical or neuropathologic evaluation, and must have had low likelihood of AD by NIA/Reagan criteria, or none/sparse NPs and NFT Braak stage of 0, I, or II. If no NPs were identified, then an NFT Braak stage III or IV was accepted in the control group (Table 2). In total 5,981 samples were eligible for inclusion in our analyses.

### Core AD neuropathologic phenotypes

The core AD neuropathologic phenotypes analyzed were NFTs and NPs. NFTs were analyzed in two well-established ordinal rankings (seven Braak stages: none, I, II, III, IV, V, and VI; and four Braak groups: none, transentorhinal, limbic, isocortical). NPs were analyzed with one ordinal ranking (four CERAD scores: none, sparse, moderate, frequent) and a presence vs. absence (any NPs vs. no NPs) analysis. Sample sizes by cohort are described in **Table S18**.

### Co-morbid neuropathologic phenotypes

There were four co-morbid neuropathologic phenotypes analyzed: LBD, VBI, HS, CAA. LBD was analyzed with two ordinal rankings (five categories: none, brainstem-predominant, limbic, neocortical, and other regions or not specified; three categories: none, brainstem-predominant, and all other regions or not specified) and a presence vs. absence (any LBD vs. no LBD) analysis. VBI was analyzed in an ordinal ranking (three categories: none, any microinfarcts, any lacunar or territorial infarcts) and in a presence vs. absence (any VBI vs. no VBI) analysis. HS and CAA were both analyzed using presence vs. absence analyses. Sample sizes by cohort are described in **Table S18**.

### Clinico-pathologic AD dementia phenotypes

Additionally, we performed a case/control analysis of clinico-pathologic AD dementia. That is, cases had clinical dementia with core AD neuropathologic changes, and controls were not clinically demented and had none or minimal AD neuropathologic changes. Two “case-control” datasets were considered. For the “primary” dataset, all cases and controls were included, regardless of the documentation from the primary neuropathologist. The secondary dataset (“complete” dataset) required more thorough documentation of the neuropathologic assessment, including documentation of the NIA/Reagan assessment or complete documentation of both the NFT Braak stage and the NP score. Sample sizes by cohort are described in **Table S18**.

### Genotyping and quality control (QC)

Genotyping for the ADC samples was performed at the Children's Hospital of Pennsylvania on the Illumina 660k beadchip. Genotyping of ADGC collaborating centers was performed on a variety of genotyping platforms and is described in Naj et al 2011 [8]. QC and statistical analyses are summarized in **Figure S22**. Preliminary QC includes checks for sample relatedness, sex inconsistency, sample missingness, and principal components analysis of genotype data to confirm the sample's race, and is described by Naj and colleagues [8], [24], [25]. The top principal components were further used as covariates in the association analysis (see below).

### Genotype imputation and final QC

To infer genotypes at loci not on the genotyping chips, we used IMPUTE v2 software [26] in all datasets, using the 1,000 Genomes Project data as a reference [27]. Imputation and imputation QC were performed independently across the different datasets, but with all using the December 2010 release of the 1,000 Genomes Data. For inclusion in the statistical analysis, variants must have high quality imputation (IMPUTE info score greater than 0.40 and a dosage variance greater than 0.0198 (the estimated variance of a 1% MAF SNP). We also required an MAF>1% (estimated based on dosage data) as the study is not well-powered for low frequency variants.

### Statistical analysis

See Supporting Material for an overview of analyses. For binary traits, logistic regression was used, with principal components 1–3 included as covariates to account for population substructure; the analysis was performed using PLINK genetic analysis software [24]. For ordinal traits, we used polytomous logistic regression with principal components 1–3 included as covariates to account for population substructure; the analysis was performed with the *polr* function (part of the *MASS* package [28]) in the R statistical analysis software (http://www.r-project.org/). Polytomous logistic regression is an extension of logistic regression that allows for analysis of categorical data. As these data were either binary or ordinal, we used the proportional odds assumption to obtain a single effect-size estimate for each SNP for each of the ordinal traits.

Association analysis was performed within each cohort separately, and results were meta-analyzed across cohorts. Because some datasets had small sample size or had incomplete phenotyping, we only considered a set for a particular analysis if that set had at least two categories with at least five individuals in each category. That is, for binary traits the set had to include both cases and controls. For categorical traits, the set had to have at least two categories on which analysis could be performed. The sets included (and categories used) in the particular analyses are described in Table S8. After the within dataset association analysis, we used METAL [29], to meta-analyze result across datasets, weighting each effect size by the inverse of the variance estimated from the logistic or polytomous logistic regression analyses, and to calculate heterogeneity statistics for each meta-analysis. QQ-plots for each trait are included (see supporting material).

To compare effect sizes at previously identified AD loci, we performed linear regression and correlation analyses of the estimated effect sizes (and OR) at the loci. Regression was performed using the R statistical software with the “glm” function [30], and the “corrgrams” package [31]. As linear regression in small datasets can be heavily influenced by outliers, we removed the outliers (plus or minus 3 standard deviations from the mean) prior to regression and correlation analyses.

## Supporting Information

Figure S1 QQ plot for primary endpoint. QQ Plot for the primary clinico-neuropathologic case-control analysis. The analysis was not inflated for false positives (GIF = 0.972).
(TIF)

Figure S2 QQ plot for complete endpoint. QQ plot for the “complete” clinico-neuropathologic case-control analysis. The analysis was not inflated for false positives (GIF = 0.949).
(TIF)

Figure S3 Regional association plot of the PHF21B locus. The purple dot indicates the most associated SNP in the region. The x-axis is basepair position, and y-axis is the −log(p-value), base 10.
(TIF)

Figure S4 QQ plot for NFT Braak (four category) endpoint. QQ plot for the NFT Braak (four category ordinal) analysis. The analysis was not inflated for false positives (GIF = 0.975).
(TIF)

Figure S5 QQ plot for NFT Braak (seven category) endpoint. QQ plot for the NFT Braak (seven category ordinal) analysis. The analysis was not inflated for false positives (GIF = 0.987).
(TIF)

Figure S6 QQ plot for Neuritic plaque (any-none) endpoint. QQ plot for the Neuritic plaque (case-control) analysis. The analysis was not inflated for false positives (GIF = 0.962).
(TIF)

Figure S7 QQ plot for Neuritic plaque (ordinal) endpoint. QQ plot for the Neuritic plaque (ordinal) analysis. The analysis was not inflated for false positives (GIF = 0.977).
(TIF)

Figure S8 Forest plot for GALNT7 locus and Neuritic plaque(rs62341097). Forest plot of the rs62341097 variant, in terms of the odds ratio.
(TIF)

Figure S9 Forest Plot for ABCG1 locus and Neuritic plaque (chr21:43,678,066). Forest plot of the associated chr21 locus, in terms of the odds ratio.
(TIF)

Figure S10 Forest Plot for chr9 locus and Neuritic plaque (chr9:129,356,304). Forest plot of the associated chr9 locus, in terms of the odds ratio.
(TIF)

Figure S11 QQ plot for Lewy Body (any-none) endpoint. QQ plot for the Lewy Body (case-control) analysis. The analysis was not inflated for false positives (GIF = 0.954).
(TIF)

Figure S12 QQ plot for Lewy Body (three category) endpoint. QQ plot for the Lewy Body (three category ordinal) analysis. The analysis was not inflated for false positives (GIF = 0.956).
(TIF)

Figure S13 QQ plot for Lewy Body (five category) endpoint. QQ plot for the Lewy Body(five category ordinal) analysis. The analysis was not inflated for false positives (GIF = 0.963).
(TIF)

Figure S14 QQ plot for Amyloid Angiopathy endpoint. QQ plot for the Amyloid Angiopathy (case-control) analysis. The analysis was not inflated for false positives (GIF = 0.956).
(TIF)

Figure S15 QQ plot for Hippocampal Sclerosis endpoint. QQ plot for the Hippocampal Sclerosis (case-control) analysis. The analysis was not inflated for false positives (GIF = 0.968).
(TIF)

Figure S16 QQ plot for vascular brain injury (any-none) endpoint. QQ plot for the VBI (case-control) analysis. The analysis was not inflated for false positives (GIF = 0.967).
(TIF)

Figure S17 QQ plot for vascular brain injury (ordinal) endpoint. QQ plot for the VBI (ordinal) analysis. The analysis was not inflated for false positives (GIF = 0.967).
(TIF)

Figure S18 Forest plot of the KCNMB2 locus and hippocampal sclerosis (rs9637454). Forest plot of the associated rs9637454 locus, in terms of the odds ratio.
(TIF)

Figure S19 Correlation of IGAP reported effect sizes and core AD neuropathology effect sizes. Regression of effect size estimates (betas) against those previously reported for the core neuropathology features.
(TIF)

Figure S20 Correlation of IGAP reported effect sizes and Lewy Body neuropathology effect sizes. Regression of effect size estimates (betas) against those previously reported for Lewy Body features.
(TIF)

Figure S21 Correlation of IGAP reported effect sizes and vascular brain injury effect sizes. Regression of effect size estimates (betas) against those previously reported for VBI features.
(TIF)

Figure S22 Analysis workflow. Overview of the analysis process. This approach was taken for each phenotype independently of the other phenotypes.
(TIF)

Table S1 Top association signals from the primary case-control phenotype. Chr: chromosome number; EA: effect allele; RA: reference allele; Freq: frequency of effect allele; min/maxFreq: the minimum and maximum within cohort allele frequency; Effect: allele effect, in terms of the beta coefficient.
(PDF)

Table S2 Top association signals from the complete case-control phenotype. Chr: chromosome number; EA: effect allele; RA: reference allele; Freq: frequency of effect allele; min/maxFreq: the minimum and maximum within cohort allele frequency; Effect: allele effect, in terms of the beta coefficient.
(PDF)

Table S3 Top association signals from the neurofibrillary tangle (NFT) Braak ordinal I phenotype. Chr: chromosome number; EA: effect allele; RA: reference allele; Freq: frequency of effect allele; min/maxFreq: the minimum and maximum within cohort allele frequency; Effect: allele effect, in terms of the beta coefficient.
(PDF)

Table S4 Top association signals from the neurofibrillary tangle (NFT) Braak ordinal II phenotype. Chr: chromosome number; EA: effect allele; RA: reference allele; Freq: frequency of effect allele; min/maxFreq: the minimum and maximum within cohort allele frequency; Effect: allele effect, in terms of the beta coefficient.
(PDF)

Table S5 Top association signals from the neuritic plaque ordinal phenotype. Chr: chromosome number; EA: effect allele; RA: reference allele; Freq: frequency of effect allele; min/maxFreq: the minimum and maximum within cohort allele frequency; Effect: allele effect, in terms of the beta coefficient.
(PDF)

Table S6 Top association signals from the neuritic plaque case-control phenotype. Chr: chromosome number; EA: effect allele; RA: reference allele; Freq: frequency of effect allele; min/maxFreq: the minimum and maximum within cohort allele frequency; Effect: allele effect, in terms of the beta coefficient.
(PDF)

Table S7 Top association signals from the Lewy body disease (LBD) ordinal I phenotype. Chr: chromosome number; EA: effect allele; RA: reference allele; Freq: frequency of effect allele; min/maxFreq: the minimum and maximum within cohort allele frequency; Effect: allele effect, in terms of the beta coefficient.
(PDF)

Table S8 Top association signals from the Lewy body disease (LBD) case-control phenotype. Chr: chromosome number; EA: effect allele; RA: reference allele; Freq: frequency of effect allele; min/maxFreq: the minimum and maximum within cohort allele frequency; Effect: allele effect, in terms of the beta coefficient.
(PDF)

Table S9 Top association signals from the Lewy body disease (LBD) ordinal II phenotype. Chr: chromosome number; EA: effect allele; RA: reference allele; Freq: frequency of effect allele; min/maxFreq: the minimum and maximum within cohort allele frequency; Effect: allele effect, in terms of the beta coefficient.
(PDF)

Table S10 Top association signals from the vascular brain injury (VBI) case-control phenotype. Chr: chromosome number; EA: effect allele; RA: reference allele; Freq: frequency of effect allele; min/maxFreq: the minimum and maximum within cohort allele frequency; Effect: allele effect, in terms of the beta coefficient.
(PDF)

Table S11 Top association signals from the vascular brain injury (VBI) ordinal phenotype. Chr: chromosome number; EA: effect allele; RA: reference allele; Freq: frequency of effect allele; min/maxFreq: the minimum and maximum within cohort allele frequency; Effect: allele effect, in terms of the beta coefficient.
(PDF)

Table S12 Top association signals from the hippocampal sclerosis (HS) case-control phenotype. Chr: chromosome number; EA: effect allele; RA: reference allele; Freq: frequency of effect allele; min/maxFreq: the minimum and maximum within cohort allele frequency; Effect: allele effect, in terms of the beta coefficient.
(PDF)

Table S13 Top association signals from the cerebral amyloid angiopathy (CAA) case-control phenotype. Chr: chromosome number; EA: effect allele; RA: reference allele; Freq: frequency of effect allele; min/maxFreq: the minimum and maximum within cohort allele frequency; Effect: allele effect, in terms of the beta coefficient.
(PDF)

Table S14 Association of common AD risk variants with core AD neuropathologic features. Bold text indicates p-values meeting an alpha = 0.05 threshold, uncorrected for multiple testing.
(PDF)

Table S15 Association of common AD risk variants with coincident neuropathologic features. Bold text indicates p-values meeting an alpha = 0.05 threshold; uncorrected for multiple testing.
(PDF)

Table S16 Cohort contact information. ACT: Adult Changes in Thought Study; ADC: Alzheimer's Disease Center; MAYO: Mayo Clinic Alzheimer's Disease Research Center; MBB: University of Miami Brain Endowment Bank; NIA-LOAD: National Institute on Aging Late–Onset Alzheimer's Disease Family Study; OHSU: Oregon Health & Science University Alzheimer's Disease Center; ROSMAP: Religious Orders Study and Memory and Aging Project; TGEN: Translational Genomics Research Institute; UM/VU/MSSM: University of Miami Hussman Institute for Human Genomics/Vanderbilt University Center for Human Genetics Research/Mount Sinai School of Medicine; UP: University of Pittsburgh Alzheimer's Disease Research Center; ADGC: Alzheimer's Disease Genetics Consortium; dbGAP: database of genotypes and phenotypes; eMERGE: electronic medical records and genomics; NACC: National Alzheimer's Coordinating Center; NCRAD: National Cell Repository for Alzheimer's Disease.
(PDF)

Table S17 Descriptive statistics of cohorts. AAO: age at onset; AAD: age at death; AAE: age at exam; SD: standard deviation. APOE: relative frequency of APOE genotypes where * represents E2 or E3. Cohorts: ACT: Adult Changes in Thought Study; ADC: Alzheimer's Disease Center; TGEN: Translational Genomics Research Institute; LOAD: National Institute on Aging Late-Onset Alzheimer's Disease Family Study; MAYO: Mayo Clinic Alzheimer's Disease Research Center; ROSMAP: Religious Orders Study and Memory and Aging Project; UPITT: University of Pittsburgh Alzheimer's Disease Research Center; UM/MASH: University of Miami Brain Endowment Bank; OHSU: Oregon Health & Science University Alzheimer's Disease Center; UM/VU/MSSM: University of Miami Hussman Institute for Human Genomics/Vanderbilt University Center for Human Genetics Research/Mount Sinai School of Medicine.
(PDF)

Table S18 Sample size and cohort inclusion by phenotype. A missing point (“.”) indicates the category had fewer than 5 observations and was not included in the analysis. A cohort must have 5 observations in two or more categories to be included in a particular analysis. Ordinal traits were coded in the order listed here. ACT: Adult Changes in Thought Study; ADC: Alzheimer's Disease Center; OHSU: Oregon Health & Science University Alzheimer's Disease Center; MAYO: Mayo Clinic Alzheimer's Disease Research Center; MBB: University of Miami Brain Endowment Bank; NIA-LOAD: National Institute on Aging Late-Onset Alzheimer's Disease Family Study; ROSMAP: Religious Orders Study and Memory and Aging Project; TGEN: Translational Genomics Research Institute; UM/VU/MSSM: University of Miami Hussman Institute for Human Genomics/Vanderbilt University Center for Human Genetics Research/Mount Sinai School of Medicine; UP: University of Pittsburgh Alzheimer's Disease Research Center.
(PDF)

Text S1 Additional Alzheimer's Disease Genetics Consortium (ADGC) members and affiliations.
(DOCX)

## References

[1] Sonnen JA, Larson EB, Crane PK, Haneuse S, Li G, et al (2007) Pathological correlates of dementia in a longitudinal, population-based sample of aging. Ann Neurol 62: 406–413. 17879383 10.1002/ana.21208

[2] Sonnen JA, Santa Cruz K, Hemmy LS, Woltjer R, Leverenz JB, et al (2011) Ecology of the aging human brain. Arch Neurol 68: 1049–1056. 21825242 10.1001/archneurol.2011.157 PMC3218566

[3] Jack CR Jr, Knopman DS, Jagust WJ, Shaw LM, Aisen PS, et al (2010) Hypothetical model of dynamic biomarkers of the Alzheimer's pathological cascade. Lancet Neurol 9: 119–128. 20083042 10.1016/S1474-4422(09)70299-6 PMC2819840

[4] Hurd MD, Martorell P, Delavande A, Mullen KJ, Langa KM (2013) Monetary costs of dementia in the United States. N Engl J Med 368: 1326–1334. 23550670 10.1056/NEJMsa1204629 PMC3959992

[5] Jun G, Naj AC, Beecham GW, Wang LS, Buros J, et al (2010) Meta-analysis confirms CR1, CLU, and PICALM as alzheimer disease risk loci and reveals interactions with APOE genotypes. Arch Neurol 67: 1473–1484. 20697030 10.1001/archneurol.2010.201 PMC3048805

[6] Naj AC, Beecham GW, Martin ER, Gallins PJ, Powell EH, et al (2010) Dementia revealed: novel chromosome 6 locus for late-onset Alzheimer disease provides genetic evidence for folate-pathway abnormalities. PLoS Genet 6: e1001130. 20885792 10.1371/journal.pgen.1001130 PMC2944795

[7] Hollingworth P, Harold D, Sims R, Gerrish A, Lambert JC, et al (2011) Common variants at ABCA7, MS4A6A/MS4A4E, EPHA1, CD33 and CD2AP are associated with Alzheimer's disease. Nat Genet 43: 429–435. 21460840 10.1038/ng.803 PMC3084173

[8] Naj AC, Jun G, Beecham GW, Wang LS, Vardarajan BN, et al (2011) Common variants at MS4A4/MS4A6E, CD2AP, CD33 and EPHA1 are associated with late-onset Alzheimer's disease. Nat Genet 43: 436–441. 21460841 10.1038/ng.801 PMC3090745

[9] Jun G, Vardarajan BN, Buros J, Yu CE, Hawk MV, et al (2012) Comprehensive search for Alzheimer disease susceptibility loci in the APOE region. Arch Neurol 69: 1270–1279. 22869155 10.1001/archneurol.2012.2052 PMC3579659

[10] Miyashita A, Koike A, Jun G, Wang LS, Takahashi S, et al (2013) SORL1 is genetically associated with late-onset Alzheimer's disease in Japanese, Koreans and Caucasians. PLoS One 8: e58618. 23565137 10.1371/journal.pone.0058618 PMC3614978

[11] Lambert JC, Ibrahim-Verbaas CA, Harold D, Naj AC, Sims R, et al (2013) Meta-analysis of 74,046 individuals identifies 11 new susceptibility loci for Alzheimer's disease. Nat Genet 45: 1452–1458. 24162737 10.1038/ng.2802 PMC3896259

[12] Corder EH, Saunders AM, Strittmatter WJ, Schmechel DE, Gaskell PC, et al (1993) Gene dose of apolipoprotein E type 4 allele and the risk of Alzheimer's disease in late onset families. Science 261: 921–923. 8346443 10.1126/science.8346443

[13] Benitez BA, Jin SC, Guerreiro R, Graham R, Lord J, et al (2014) Missense variant in TREML2 protects against Alzheimer's disease. Neurobiol Aging 35: 1510.e1519–1526. 24439484 10.1016/j.neurobiolaging.2013.12.010 PMC3961557

[14] Cruchaga C, Karch CM, Jin SC, Benitez BA, Cai Y, et al (2014) Rare coding variants in the phospholipase D3 gene confer risk for Alzheimer's disease. Nature 505: 550–554. 24336208 10.1038/nature12825 PMC4050701

[15] Shulman JM, Chen K, Keenan BT, Chibnik LB, Fleisher A, et al (2013) Genetic susceptibility for Alzheimer disease neuritic plaque pathology. JAMA Neurol 70: 1150–1157. 23836404 10.1001/jamaneurol.2013.2815 PMC3773291

[16] Tsuang D, Leverenz JB, Lopez OL, Hamilton RL, Bennett DA, et al (2013) APOE epsilon4 increases risk for dementia in pure synucleinopathies. JAMA Neurol 70: 223–228. 23407718 10.1001/jamaneurol.2013.600 PMC3580799

[17] Wollmer MA, Sleegers K, Ingelsson M, Zekanowski C, Brouwers N, et al (2007) Association study of cholesterol-related genes in Alzheimer's disease. Neurogenetics 8: 179–188. 17387528 10.1007/s10048-007-0087-z

[18] Espinosa B, Guevara J, Hernandez P, Slomianny MC, Guzman A, et al (2003) Characterization of an O-glycosylated plaque-associated protein from Alzheimer disease brain. J Neuropathol Exp Neurol 62: 34–41. 12528816 10.1093/jnen/62.1.34

[19] Ariga T, Kobayashi K, Hasegawa A, Kiso M, Ishida H, et al (2001) Characterization of high-affinity binding between gangliosides and amyloid beta-protein. Arch Biochem Biophys 388: 225–230. 11368158 10.1006/abbi.2001.2304

[20] Graebert KS, Popp GM, Kehle T, Herzog V (1995) Regulated O-glycosylation of the Alzheimer beta-A4 amyloid precursor protein in thyrocytes. Eur J Cell Biol 66: 39–46. 7750518

[21] Mirra SS, Hart MN, Terry RD (1993) Making the diagnosis of Alzheimer's disease. A primer for practicing pathologists. Arch Pathol Lab Med 117: 132–144. 8427562

[22] Braak H, Braak E (1991) Neuropathological stageing of Alzheimer-related changes. Acta Neuropathol 82: 239–259. 1759558 10.1007/BF00308809

[23] Hyman BT, Trojanowski JQ (1997) Consensus recommendations for the postmortem diagnosis of Alzheimer disease from the National Institute on Aging and the Reagan Institute Working Group on diagnostic criteria for the neuropathological assessment of Alzheimer disease. J Neuropathol Exp Neurol 56: 1095–1097. 9329452 10.1097/00005072-199710000-00002

[24] Purcell S, Neale B, Todd-Brown K, Thomas L, Ferreira MA, et al (2007) PLINK: a tool set for whole-genome association and population-based linkage analyses. Am J Hum Genet 81: 559–575. 17701901 10.1086/519795 PMC1950838

[25] Price AL, Patterson NJ, Plenge RM, Weinblatt ME, Shadick NA, et al (2006) Principal components analysis corrects for stratification in genome-wide association studies. Nat Genet 38: 904–909. 16862161 10.1038/ng1847

[26] Marchini J, Howie B, Myers S, McVean G, Donnelly P (2007) A new multipoint method for genome-wide association studies by imputation of genotypes. Nat Genet 39: 906–913. 17572673 10.1038/ng2088

[27] Abecasis GR, Auton A, Brooks LD, DePristo MA, Durbin RM, et al (2012) An integrated map of genetic variation from 1,092 human genomes. Nature 491: 56–65. 23128226 10.1038/nature11632 PMC3498066

[28] Venables W, Ripley D (2003) Modern Applied Statistics with S. New York: Springer. 497 p.

[29] Willer CJ, Li Y, Abecasis GR (2010) METAL: fast and efficient meta-analysis of genomewide association scans. Bioinformatics 26: 2190–2191. 20616382 10.1093/bioinformatics/btq340 PMC2922887

[30] Hastie T, Pregibon D (1992) Generalized linear models. In: Chambers J, Hastie T, editors. Statistical Models in S. Pacific Grove, CA: Wadsworth & Brooks/Cole. pp. 195–246.

[31] Friendly M (2002) Corrgrams: Exploratory displays for correlation matrices. American Statistician 56: 316–324.

