# Correction: Genome-Wide Association Meta-analysis of Neuropathologic Features of Alzheimer’s Disease and Related Dementias

**DOI:** 10.1371/journal.pgen.1004867

**Published:** 2014-11-12

**Authors:** The PLOS Genetics Staff

Supplementary Text S1 contained an incorrect list of the Alzheimer's Disease Genetics Consortium members and affiliations. The correct list is provided below.

## Supporting Information

Text S1 Additional Alzheimer's Disease Genetics Consortium (ADGC) members and affiliations (updated).
(DOCX)
