# Supplementary figures and images for: Genome-Wide Association Meta-analysis of Neuropathologic Features of Alzheimer's Disease and Related Dementias
Source: PLoS Genet. 2014 Sep 4;10(9):e1004606. doi: 10.1371/journal.pgen.1004606 (PMC4154667; doi:10.1371/journal.pgen.1004606)

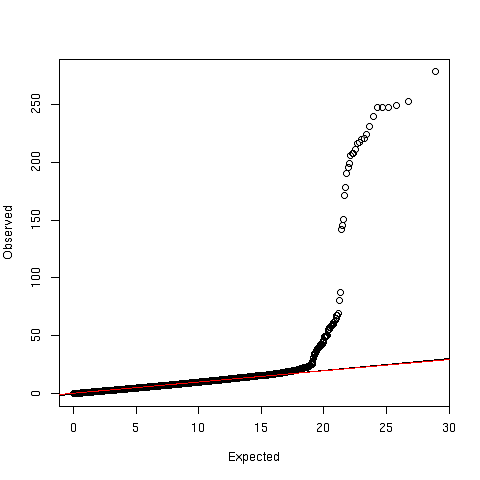

Supplement: Figure S1 — QQ plot for primary endpoint. QQ Plot for the primary clinico-neuropathologic case-control analysis. The analysis was not inflated for false positives (GIF = 0.972). (TIF) [file pgen.1004606.s001.tif]

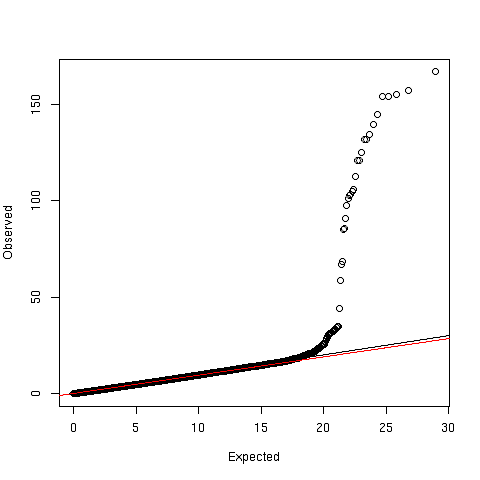

Supplement: Figure S2 — QQ plot for complete endpoint. QQ plot for the “complete” clinico-neuropathologic case-control analysis. The analysis was not inflated for false positives (GIF = 0.949). (TIF) [file pgen.1004606.s002.tif]

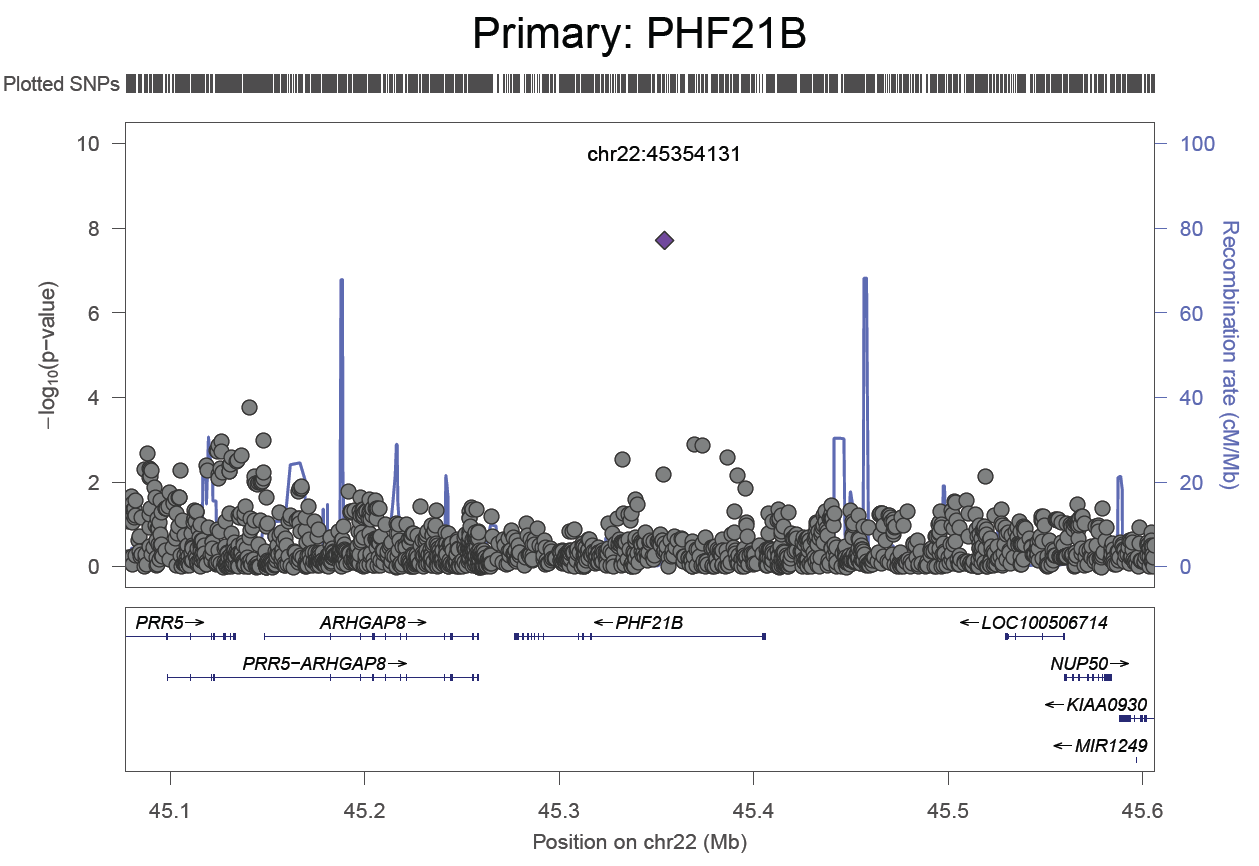

Supplement: Figure S3 — Regional association plot of the PHF21B locus. The purple dot indicates the most associated SNP in the region. The x-axis is basepair position, and y-axis is the −log(p-value), base 10. (TIF) [file pgen.1004606.s003.tif]

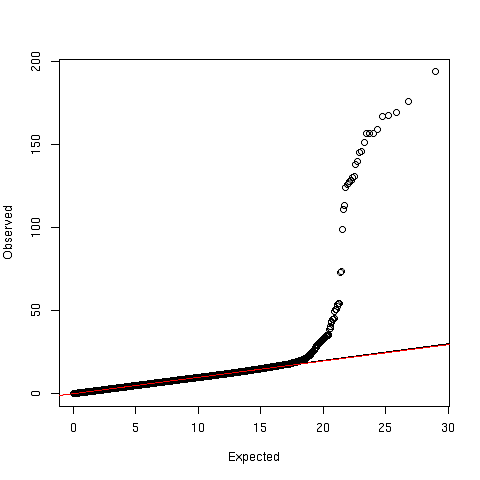

Supplement: Figure S4 — QQ plot for NFT Braak (four category) endpoint. QQ plot for the NFT Braak (four category ordinal) analysis. The analysis was not inflated for false positives (GIF = 0.975). (TIF) [file pgen.1004606.s004.tif]

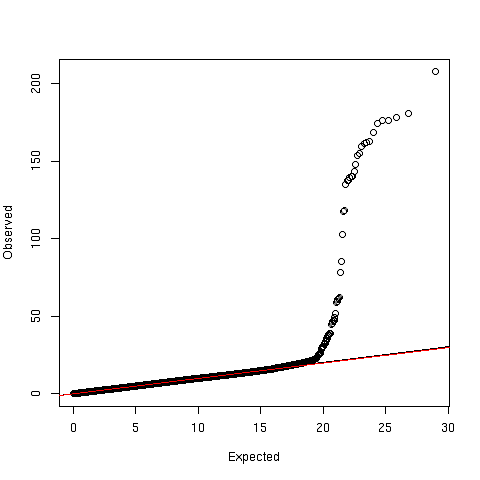

Supplement: Figure S5 — QQ plot for NFT Braak (seven category) endpoint. QQ plot for the NFT Braak (seven category ordinal) analysis. The analysis was not inflated for false positives (GIF = 0.987). (TIF) [file pgen.1004606.s005.tif]

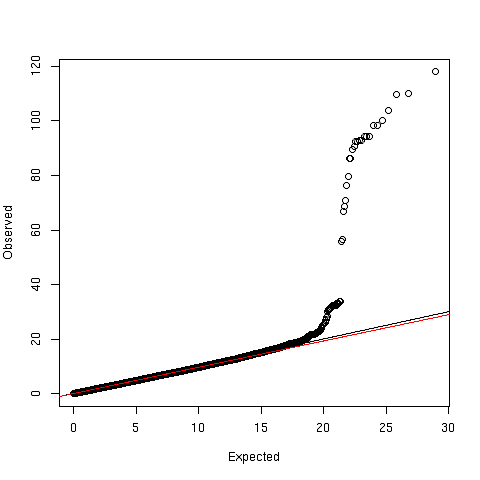

Supplement: Figure S6 — QQ plot for Neuritic plaque (any-none) endpoint. QQ plot for the Neuritic plaque (case-control) analysis. The analysis was not inflated for false positives (GIF = 0.962). (TIF) [file pgen.1004606.s006.tif]

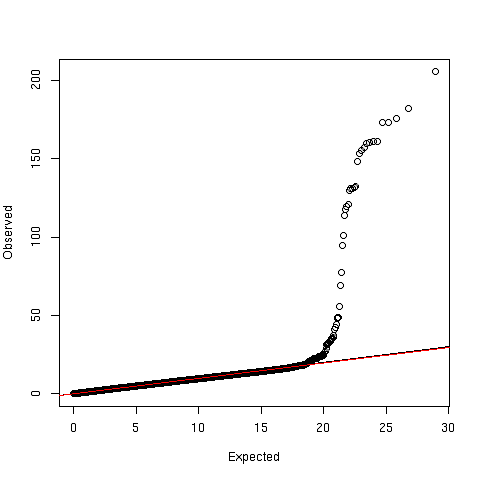

Supplement: Figure S7 — QQ plot for Neuritic plaque (ordinal) endpoint. QQ plot for the Neuritic plaque (ordinal) analysis. The analysis was not inflated for false positives (GIF = 0.977). (TIF) [file pgen.1004606.s007.tif]

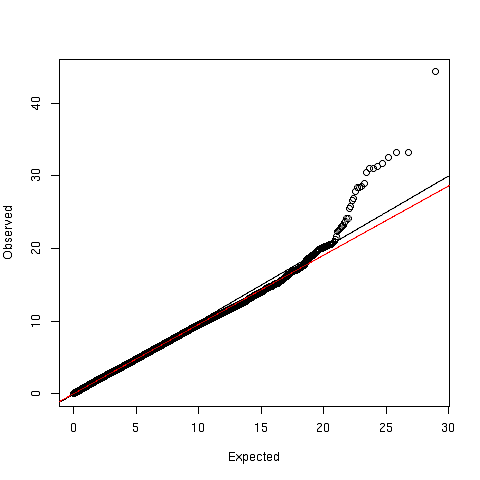

Supplement: Figure S11 — QQ plot for Lewy Body (any-none) endpoint. QQ plot for the Lewy Body (case-control) analysis. The analysis was not inflated for false positives (GIF = 0.954). (TIF) [file pgen.1004606.s011.tif]

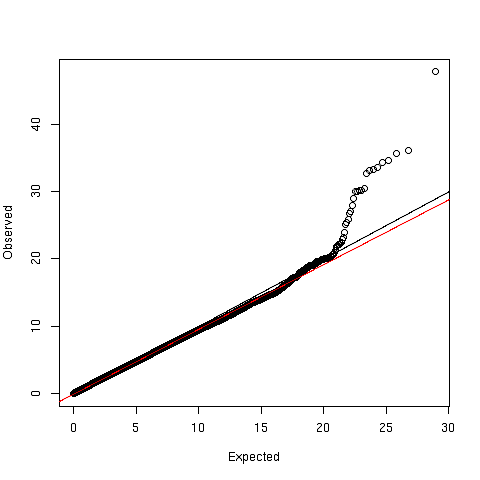

Supplement: Figure S12 — QQ plot for Lewy Body (three category) endpoint. QQ plot for the Lewy Body (three category ordinal) analysis. The analysis was not inflated for false positives (GIF = 0.956). (TIF) [file pgen.1004606.s012.tif]

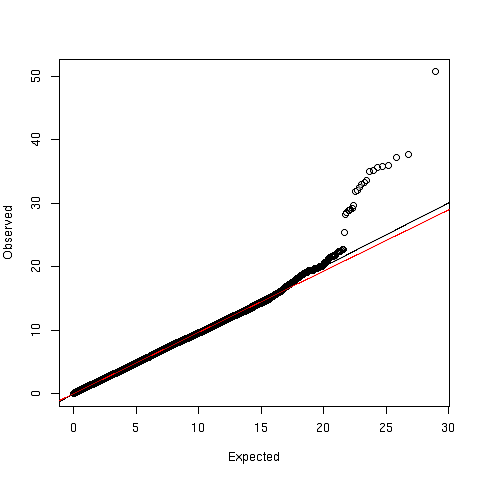

Supplement: Figure S13 — QQ plot for Lewy Body (five category) endpoint. QQ plot for the Lewy Body(five category ordinal) analysis. The analysis was not inflated for false positives (GIF = 0.963). (TIF) [file pgen.1004606.s013.tif]

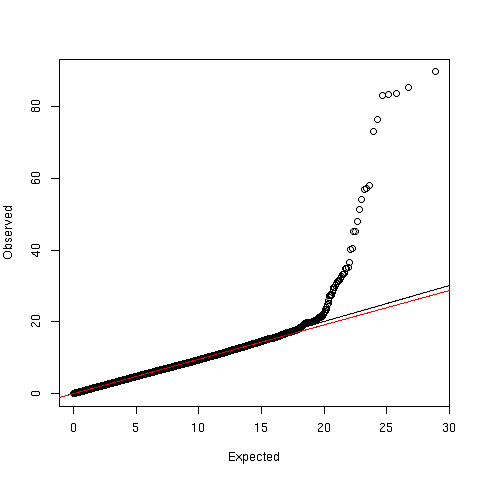

Supplement: Figure S14 — QQ plot for Amyloid Angiopathy endpoint. QQ plot for the Amyloid Angiopathy (case-control) analysis. The analysis was not inflated for false positives (GIF = 0.956). (TIF) [file pgen.1004606.s014.tif]

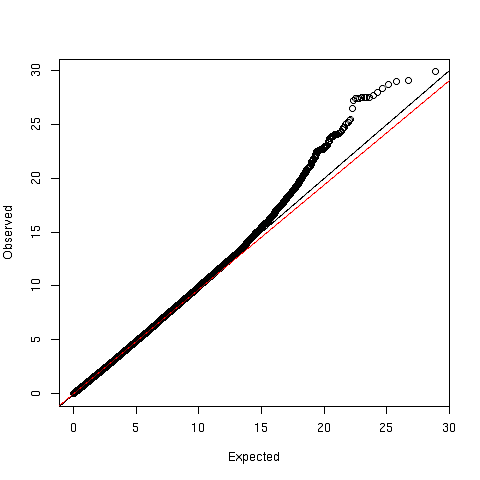

Supplement: Figure S15 — QQ plot for Hippocampal Sclerosis endpoint. QQ plot for the Hippocampal Sclerosis (case-control) analysis. The analysis was not inflated for false positives (GIF = 0.968). (TIF) [file pgen.1004606.s015.tif]

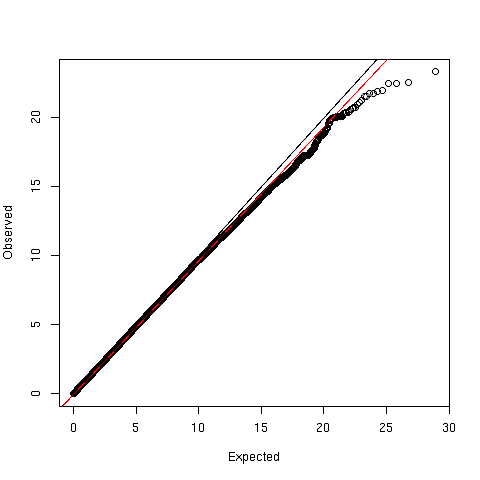

Supplement: Figure S16 — QQ plot for vascular brain injury (any-none) endpoint. QQ plot for the VBI (case-control) analysis. The analysis was not inflated for false positives (GIF = 0.967). (TIF) [file pgen.1004606.s016.tif]

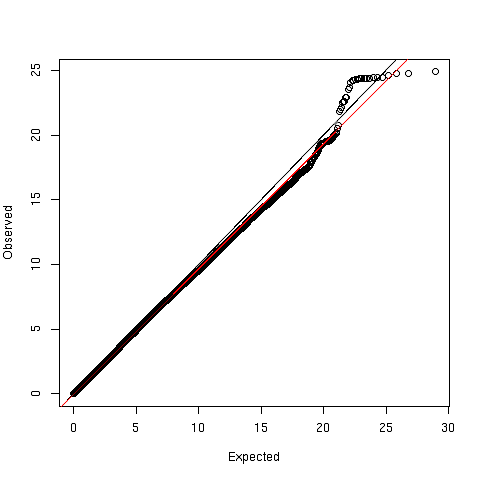

Supplement: Figure S17 — QQ plot for vascular brain injury (ordinal) endpoint. QQ plot for the VBI (ordinal) analysis. The analysis was not inflated for false positives (GIF = 0.967). (TIF) [file pgen.1004606.s017.tif]

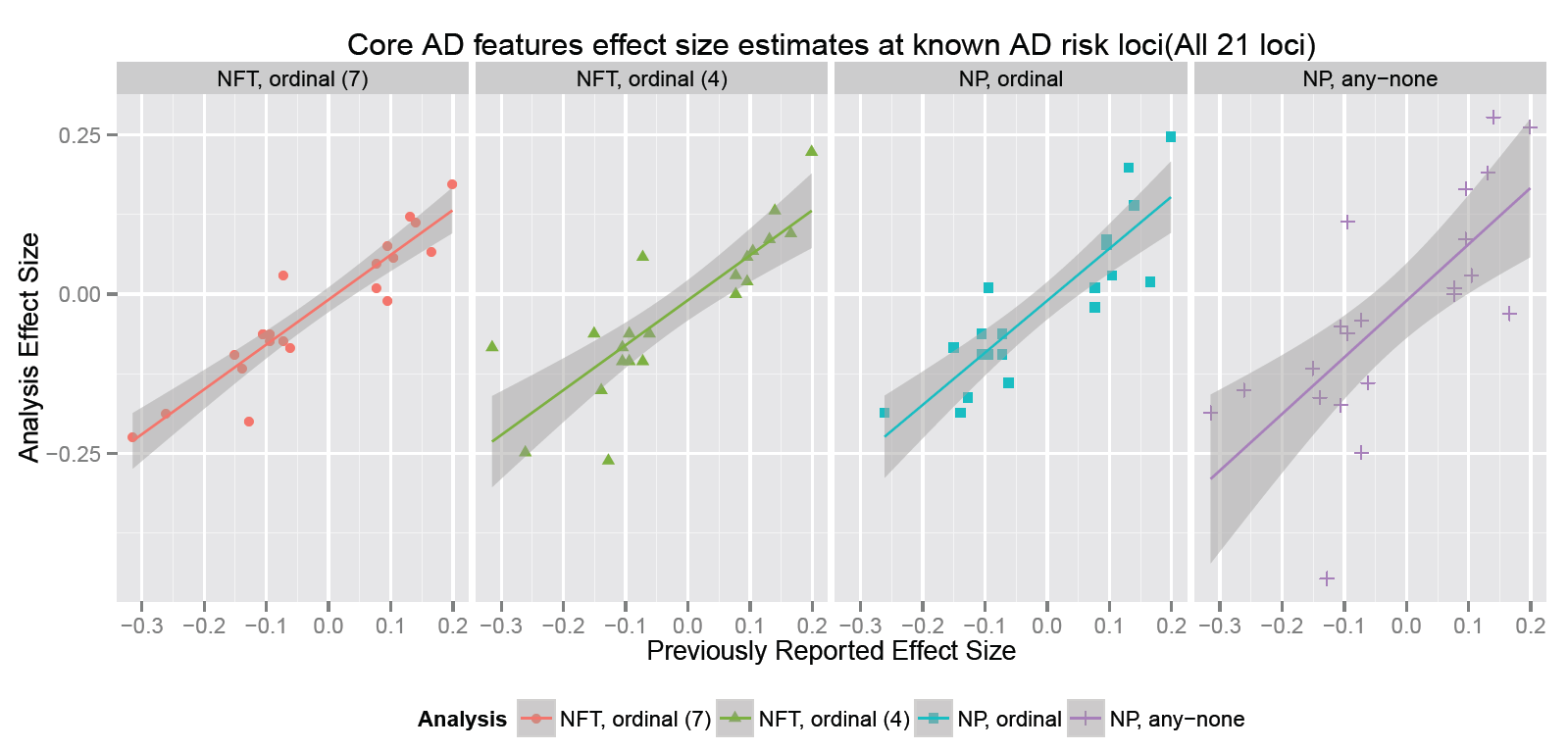

Supplement: Figure S19 — Correlation of IGAP reported effect sizes and core AD neuropathology effect sizes. Regression of effect size estimates (betas) against those previously reported for the core neuropathology features. (TIF) [file pgen.1004606.s019.tif]

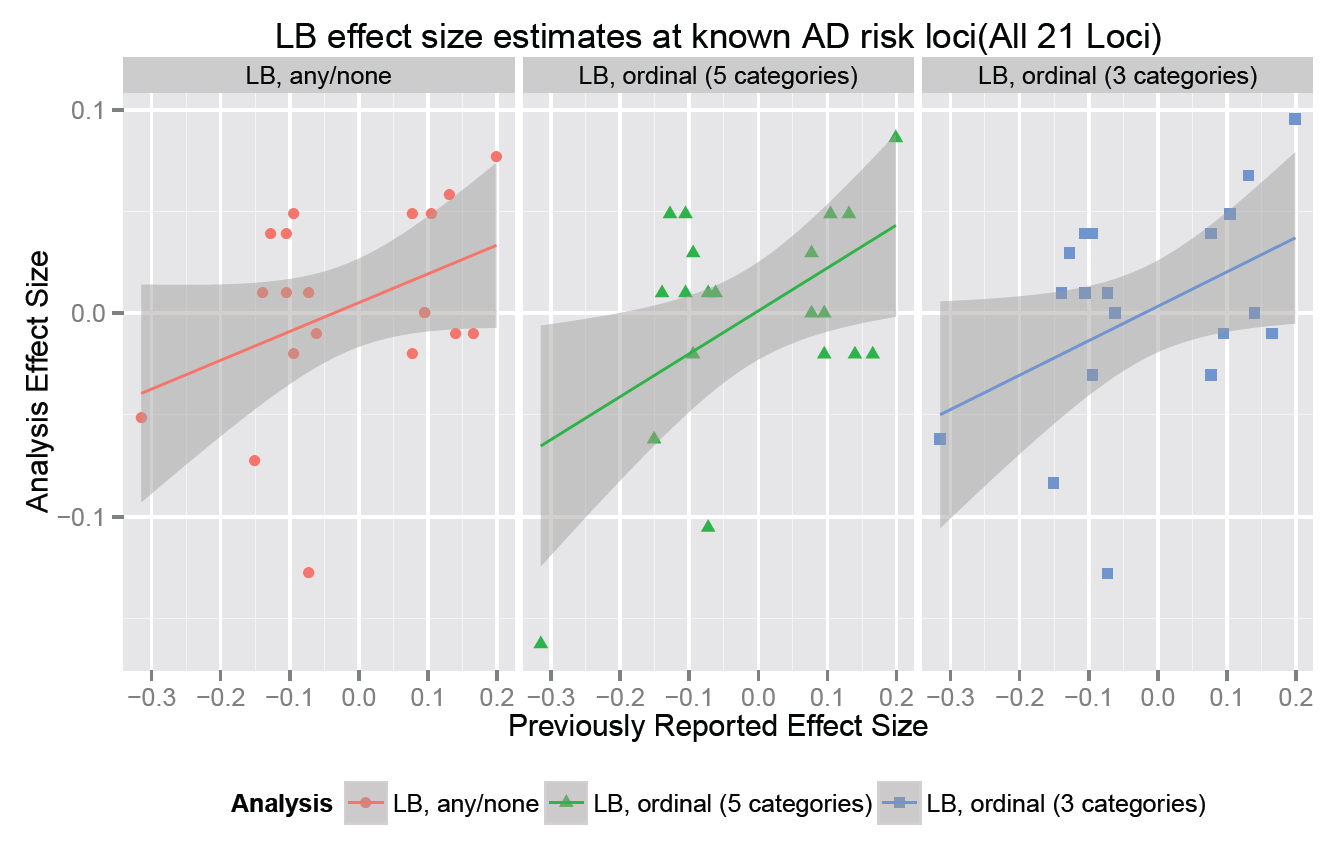

Supplement: Figure S20 — Correlation of IGAP reported effect sizes and Lewy Body neuropathology effect sizes. Regression of effect size estimates (betas) against those previously reported for Lewy Body features. (TIF) [file pgen.1004606.s020.tif]

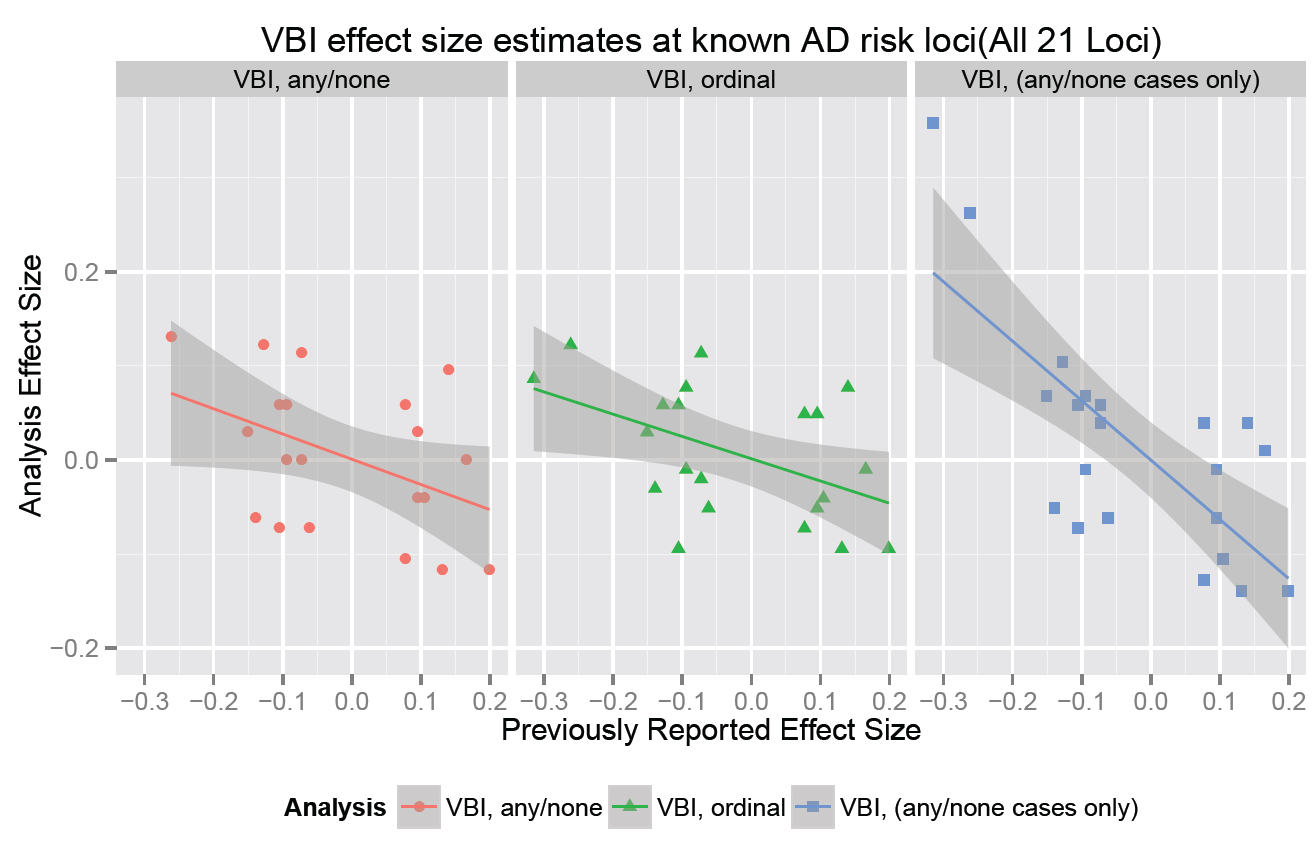

Supplement: Figure S21 — Correlation of IGAP reported effect sizes and vascular brain injury effect sizes. Regression of effect size estimates (betas) against those previously reported for VBI features. (TIF) [file pgen.1004606.s021.tif]

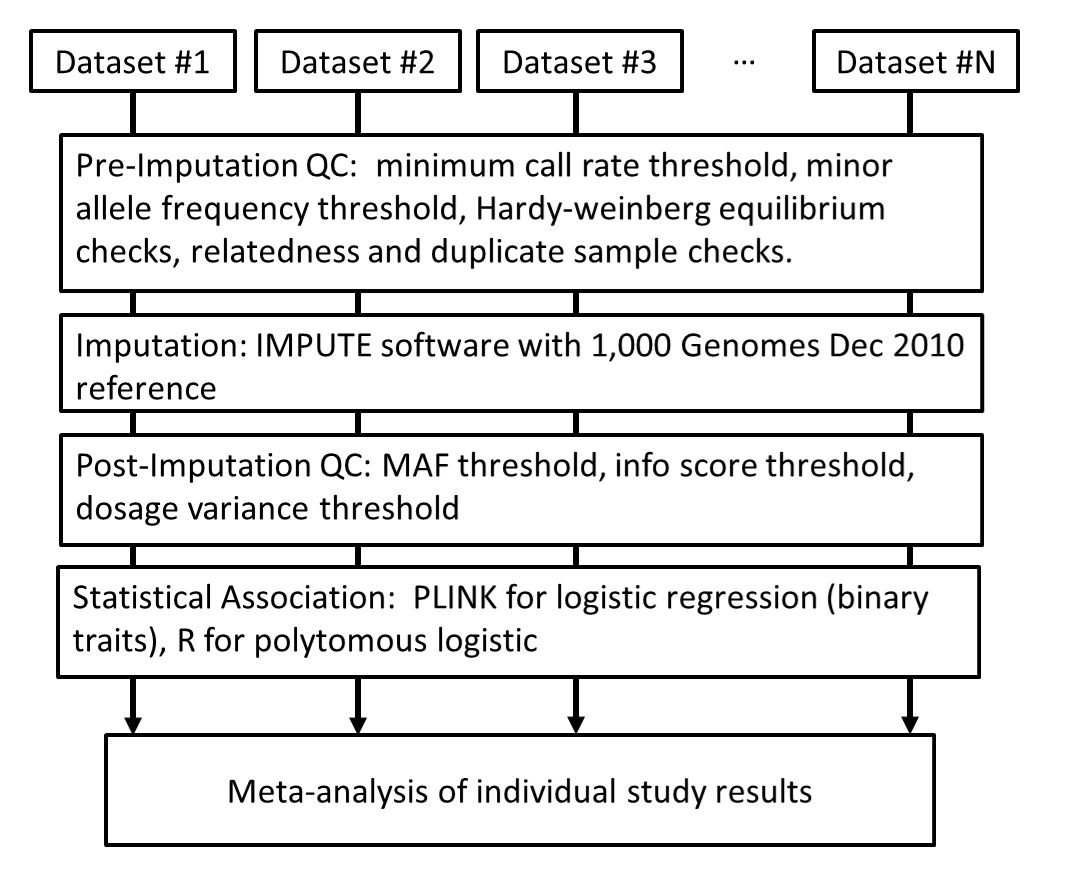

Supplement: Figure S22 — Analysis workflow. Overview of the analysis process. This approach was taken for each phenotype independently of the other phenotypes. (TIF) [file pgen.1004606.s022.tif]
