# Supplementary material for: Genome-Wide Association Meta-analysis of Neuropathologic Features of Alzheimer's Disease and Related Dementias
Source: PLoS Genet. 2014 Sep 4;10(9):e1004606. doi: 10.1371/journal.pgen.1004606 (PMC4154667; doi:10.1371/journal.pgen.1004606)
Supplement: Table S1 — Top association signals from the primary case-control phenotype. Chr: chromosome number; EA: effect allele; RA: reference allele; Freq: frequency of effect allele; min/maxFreq: the minimum and maximum within cohort allele frequency; Effect: allele effect, in terms of the beta coefficient. (PDF) [file pgen.1004606.s023.pdf]

Table S1: Top association signals from the primary case-control phenotype

Marker	Chr	Position	EA	RA	Freq	minFreq	maxFreq	Effect	StdErr	Pval	Direction	Gene
rs6857	19	45,392,254	T	C	0.2883	0.1465	0.3948	1.6136	0.0968	2.02E-62	++++++?-	PVRL2
22-45354131	22	45,354,131	A	G	0.0172	0.0172	0.0172	-2.4084	0.4288	1.90E-08	?-??????	PHF21B
rs1741309	20	4,144,577	A	G	0.3847	0.356	0.4097	0.3232	0.0659	9.43E-07	-+++++?+	SMOX
rs12604324	18	21,744,432	A	G	0.0201	0.0117	0.0372	-1.8953	0.3891	1.11E-06	--?-????	OSBPL1A
16-87690482	16	87,690,482	T	C	0.0116	0.0116	0.012	-2.0387	0.4245	1.56E-06	--??????	JPH3
rs4846835	1	230,281,156	A	G	0.1959	0.1844	0.228	-0.3966	0.0826	1.59E-06	------?-	GALNT2
rs9969783	9	4,888,441	A	C	0.4562	0.3864	0.492	-0.2949	0.0615	1.63E-06	-------+	none
rs3779483	7	95,455,845	T	C	0.0553	0.0553	0.0553	-1.2925	0.2716	1.96E-06	?-??????	DYNC1I1
rs11158198	14	58,576,320	C	A	0.492	0.4141	0.519	-0.286	0.0601	1.97E-06	------?-	C14orf37
rs55643152	14	50,486,791	T	C	0.0402	0.0324	0.045	-1.0479	0.2236	2.78E-06	----?-??	none
rs6733839	2	127,892,810	T	C	0.43	0.3844	0.4533	0.3288	0.0708	3.42E-06	+++++-++	none
10-824004	10	824,004	G	T	0.9846	0.9829	0.9864	1.3723	0.2957	3.46E-06	+++-????	none
20-1193706	20	1,193,706	A	G	0.0301	0.0192	0.0417	-1.1614	0.2504	3.53E-06	--?--??+	none
rs7239679	18	10,443,693	T	C	0.45	0.4414	0.4702	0.2971	0.0644	3.95E-06	++++++++	none
5-161442770	5	161,442,770	T	C	0.0136	0.0129	0.0159	-1.8342	0.4009	4.76E-06	--?-????	none
rs6835098	4	174,089,238	T	C	0.3308	0.3207	0.3609	-0.2778	0.0609	5.05E-06	------+-	none
rs17539289	12	41,126,330	A	C	0.0535	0.0434	0.0641	-0.6468	0.142	5.21E-06	-------?	CNTN1
12-34593353	12	34,593,353	A	C	0.0745	0.0745	0.0745	-1.274	0.2798	5.30E-06	?-??????	none
8-3777515	8	3,777,515	C	T	0.965	0.965	0.965	1.6687	0.3677	5.66E-06	?+??????	CSMD1
22-36621624	22	36,621,624	T	G	0.0224	0.0137	0.0243	-1.2016	0.2652	5.85E-06	------??	none
21-43678066	21	43,678,066	T	C	0.0107	0.0107	0.0109	-1.6947	0.3759	6.52E-06	--??????	ABCG1
14-97513230	14	97,513,230	T	C	0.0664	0.0664	0.0664	-1.057	0.2346	6.60E-06	?-??????	none
rs3850579	5	141,864,373	G	A	0.5266	0.4946	0.568	0.2647	0.059	7.16E-06	+++++++-	none
11-37229442	11	37,229,442	C	T	0.9625	0.8845	0.9858	1.3744	0.3068	7.46E-06	?+????-?	none
rs16898904	5	29,368,024	T	C	0.054	0.0409	0.0724	0.6617	0.1477	7.50E-06	+++-+++?	none
6-106452945	6	106,452,945	C	A	0.9873	0.9859	0.9877	2.1702	0.4851	7.68E-06	?+?+????	none
2-55470453	2	55,470,453	C	T	0.9879	0.9876	0.9888	1.6314	0.3667	8.64E-06	?+?+????	MTIF2
rs61174036	3	190,848,752	G	A	0.7102	0.6692	0.7296	0.289	0.065	8.71E-06	++++++++	none
rs4533502	2	52,452,204	A	G	0.3106	0.2785	0.3305	0.2875	0.0649	9.54E-06	++++++?-	none

Chr: chromosome number; EA: effect allele; RA: reference allele; Freq: frequency of effect allele; min/maxFreq: the minimum and maximum within cohort allele frequency; Effect: allele effect, in terms of the beta coefficient
