# Supplementary material for: Genome-Wide Association Meta-analysis of Neuropathologic Features of Alzheimer's Disease and Related Dementias
Source: PLoS Genet. 2014 Sep 4;10(9):e1004606. doi: 10.1371/journal.pgen.1004606 (PMC4154667; doi:10.1371/journal.pgen.1004606)
Supplement: Table S2 — Top association signals from the complete case-control phenotype. Chr: chromosome number; EA: effect allele; RA: reference allele; Freq: frequency of effect allele; min/maxFreq: the minimum and maximum within cohort allele frequency; Effect: allele effect, in terms of the beta coefficient. (PDF) [file pgen.1004606.s024.pdf]

Table S2: Top association signals from the complete case-control phenotype

Marker	Chr	Position	EA	RA	Freq	minFreq	maxFreq	Effect	StdErr	Pval	Direction	Gene
rs6857	19	45,392,254	T	C	0.2864	0.1465	0.3786	1.501	0.1162	3.48E-38	++++++-	PVRL2
22-45354131	22	45,354,131	A	G	0.0172	0.0172	0.0172	-2.4069	0.4288	2.00E-08	?-?????	PHF21B
11-37229442	11	37,229,442	C	T	0.9858	0.9858	0.9858	1.8518	0.3497	1.19E-07	?+?????	none
rs41526548	9	94,891,778	C	T	0.7663	0.7427	0.7745	-0.6776	0.1393	1.16E-06	??-?--?	none
16-87690482	16	87,690,482	T	C	0.0116	0.0116	0.012	-2.0377	0.4245	1.59E-06	--?????	JPH3
rs3779483	7	95,455,845	T	C	0.0554	0.0554	0.0554	-1.2911	0.2717	2.01E-06	?-?????	DYNC1I1
rs7453498	6	32,446,717	C	T	0.8656	0.8415	0.8992	0.8142	0.1746	3.09E-06	-++??+?	none
rs6822736	4	54,541,846	C	A	0.6565	0.6486	0.6751	-0.3637	0.0785	3.59E-06	-------	none
rs4846835	1	230,281,156	A	G	0.1984	0.1844	0.228	-0.4595	0.1	4.33E-06	-------	GALNT2
rs35862341	14	56,361,738	C	T	0.9866	0.963	0.9874	1.521	0.3312	4.39E-06	-+?+??+	none
rs8105265	19	2,920,705	A	G	0.1173	0.0954	0.1541	-0.5203	0.1133	4.39E-06	-------	none
rs6665019	1	25,328,009	A	G	0.1211	0.0866	0.2252	-0.5008	0.1096	4.90E-06	-------	none
12-34593353	12	34,593,353	A	C	0.0745	0.0745	0.0745	-1.2739	0.2798	5.28E-06	?-?????	none
rs3003214	1	244,605,036	A	G	0.3176	0.2266	0.3385	0.37	0.0813	5.31E-06	+++++++	ADSS
rs34487851	2	106,642,554	G	A	0.7378	0.6808	0.7924	0.4164	0.0916	5.47E-06	+++++++	none
6-106452945	6	106,452,945	C	A	0.987	0.9843	0.9877	2.2238	0.4902	5.72E-06	?+?+???	none
8-3777515	8	3,777,515	C	T	0.965	0.965	0.965	1.6673	0.3677	5.77E-06	?+?????	CSMD1
21-43693789	21	43,693,789	A	G	0.011	0.0108	0.0164	-1.6439	0.3648	6.60E-06	--?+???	ABCG1
14-97513230	14	97,513,230	T	C	0.0665	0.0665	0.0665	-1.0557	0.2347	6.84E-06	?-?????	none
rs8037790	15	92,542,831	A	G	0.23	0.2083	0.2685	-0.443	0.0988	7.39E-06	-------	SLCO3A1
14-35114172	14	35,114,172	A	G	0.0185	0.0142	0.0194	-1.5867	0.3554	8.03E-06	--?--?-	none
10-115495938	10	115,495,938	G	A	0.9881	0.9879	0.9897	1.6516	0.3713	8.68E-06	?+??++?	none
1-96582508	1	96,582,508	A	G	0.0142	0.0101	0.0166	-1.6052	0.3615	8.96E-06	?-?-?-?	none
rs7297677	12	78,852,366	T	C	0.0431	0.0301	0.0502	-0.7249	0.1638	9.58E-06	-----+-	none

Chr: chromosome number; EA: effect allele; RA: reference allele; Freq: frequency of effect allele; min/maxFreq: the minimum and maximum within cohort allele frequency; Effect: allele effect, in terms of the beta coefficient
