# Supplementary material for: Genome-Wide Association Meta-analysis of Neuropathologic Features of Alzheimer's Disease and Related Dementias
Source: PLoS Genet. 2014 Sep 4;10(9):e1004606. doi: 10.1371/journal.pgen.1004606 (PMC4154667; doi:10.1371/journal.pgen.1004606)
Supplement: Table S3 — Top association signals from the neurofibrillary tangle (NFT) Braak ordinal I phenotype. Chr: chromosome number; EA: effect allele; RA: reference allele; Freq: frequency of effect allele; min/maxFreq: the minimum and maximum within cohort allele frequency; Effect: allele effect, in terms of the beta coefficient. (PDF) [file pgen.1004606.s025.pdf]

Table S3: Top association signals from the neurofibrillary tangle (NFT) Braak ordinal I phenotype

Marker	Chr	Position	EA	RA	Freq	minFreq	maxFreq	Effect	StdErr	Pval	Direction	Gene
rs6857	19	45,392,254.00	T	C	0.3285	0.1484	0.3913	0.6632	0.046	4.73E-47	+++++++?-+	PVRL2
7-102030935	7	102,030,935.00	A	C	0.0178	0.0148	0.0201	-3.9538	0.7922	6.02E-07	-??-??????	none
rs4975209	4	130,236,771.00	C	T	0.7774	0.7488	0.8147	-0.2237	0.0465	1.49E-06	-----+-+-+	none
rs34746873	8	62,898,949.00	G	A	0.5884	0.5519	0.6436	-0.1865	0.0391	1.85E-06	-------++-	none
rs9309711	2	3,472,138.00	C	T	0.7072	0.6864	0.7295	0.2055	0.0441	3.24E-06	-++-+++++-	TTC15
rs12446940	16	3,962,620.00	A	G	0.3563	0.2727	0.3947	0.1929	0.0418	3.94E-06	+++++++?+-	none
rs10009321	4	130,236,228.00	G	T	0.796	0.7518	0.8237	-0.2186	0.0474	4.00E-06	-----+-+-+	none
rs12529314	6	164,343,726.00	T	C	0.1725	0.1558	0.229	-0.2433	0.0528	4.03E-06	--+----+--	none
1-107549757	1	107,549,757.00	T	C	0.0342	0.0272	0.0357	-0.8369	0.1838	5.31E-06	?-?-?-+???	none
rs9975691	21	14,928,355.00	G	T	0.6255	0.5526	0.7611	-0.2307	0.051	6.20E-06	+------??-	none
rs12084151	1	238,409,445.00	G	A	0.9493	0.9335	0.967	0.3896	0.0865	6.65E-06	++++--++--	none
5-161442770	5	161,442,770.00	T	C	0.0131	0.0128	0.0148	-1.3285	0.2952	6.78E-06	--?-??????	none
rs17818859	2	134,455,240.00	C	A	0.9858	0.9857	0.9884	2.5604	0.569	6.80E-06	??+?????+?	none
3-118356759	3	118,356,759.00	T	C	0.0355	0.013	0.0408	-0.6033	0.1346	7.43E-06	---+---?--	none
6-7026945	6	7,026,945.00	T	C	0.1035	0.0739	0.1338	0.2961	0.0663	7.88E-06	++++++-+-+	none
rs34075049	6	32,554,935.00	A	G	0.0522	0.0387	0.0847	-0.5887	0.1324	8.67E-06	----?----?	HLA-DRB1
4-56183854	4	56,183,854.00	C	T	0.9619	0.9507	0.9778	0.5671	0.128	9.45E-06	+++-+++?++	none

Chr: chromosome number; EA: effect allele; RA: reference allele; Freq: frequency of effect allele; min/maxFreq: the minimum and maximum within cohort allele frequency; Effect: allele effect, in terms of the beta coefficient
