# Supplementary material for: Genome-Wide Association Meta-analysis of Neuropathologic Features of Alzheimer's Disease and Related Dementias
Source: PLoS Genet. 2014 Sep 4;10(9):e1004606. doi: 10.1371/journal.pgen.1004606 (PMC4154667; doi:10.1371/journal.pgen.1004606)
Supplement: Table S4 — Top association signals from the neurofibrillary tangle (NFT) Braak ordinal II phenotype. Chr: chromosome number; EA: effect allele; RA: reference allele; Freq: frequency of effect allele; min/maxFreq: the minimum and maximum within cohort allele frequency; Effect: allele effect, in terms of the beta coefficient. (PDF) [file pgen.1004606.s026.pdf]

Table S4: Top association signals from the neurofibrillary tangle (NFT) Braak ordinal II phenotype

Marker	Chr	Position	EA	RA	Freq	minFreq	maxFreq	Effect	StdErr	Pval	Direction	Gene
rs6857	19	45,392,254	T	C	0.3219	0.1484	0.3904	0.7871	0.0565	4.83E-44	+++++++?-++	PVRL2
rs11675119	2	3,476,422	A	C	0.2699	0.2516	0.3825	-0.2541	0.0488	1.93E-07	---+-----+-	TTC15
5-161442770	5	161,442,770	T	C	0.0131	0.0128	0.0148	-1.51	0.3137	1.48E-06	--?-???????	none
7-102030935	7	102,030,935	A	C	0.0178	0.0148	0.0201	-4.0337	0.8423	1.68E-06	-??-???????	none
6-144226924	6	144,226,924	A	G	0.0165	0.0123	0.0214	-0.9204	0.1981	3.40E-06	----?+-?-??	C6orf94
rs10166461	2	127,859,413	A	G	0.1733	0.1406	0.2342	-0.2636	0.057	3.82E-06	+------+---	BIN1
3-118356759	3	118,356,759	T	C	0.0358	0.0203	0.0768	-0.6753	0.1461	3.83E-06	-------??--	none
rs34487851	2	106,642,554	G	A	0.7314	0.6929	0.7936	0.2523	0.055	4.46E-06	++++++--+-?	none
17-26640212	17	26,640,212	T	C	0.0721	0.0432	0.1085	-0.3709	0.0809	4.59E-06	----+---+-+	none
rs11654699	17	30,903,310	C	T	0.5243	0.4735	0.6209	0.2055	0.0449	4.63E-06	+++++++-+--	MYO1D
rs12595161	15	52,922,890	A	G	0.0511	0.0398	0.1158	-0.4178	0.0914	4.88E-06	----------+	KIAA1370
rs10209150	2	182,329,619	G	A	0.9795	0.948	0.9848	0.591	0.1301	5.53E-06	-+++++-??-?	ITGA4
10-29989988	10	29,989,988	T	C	0.0207	0.0102	0.0287	-0.7442	0.1659	7.24E-06	+-------?+?	SVIL
rs7339349	13	74,556,225	T	C	0.106	0.0655	0.1139	0.3467	0.0775	7.62E-06	++++++++++?	KLF12
rs7626019	3	42,270,681	T	C	0.2287	0.1478	0.2511	0.2353	0.0526	7.63E-06	+++-+++-++-	none
6-25766673	6	25,766,673	C	T	0.9842	0.9768	0.9895	0.9418	0.2111	8.11E-06	++++-++??+?	SLC17A4
rs11118993	1	206,715,851	A	G	0.4787	0.426	0.5578	-0.2051	0.0461	8.52E-06	---------+-	RASSF5
rs45576635	1	201,328,705	A	G	0.0323	0.0323	0.0323	-1.1179	0.2513	8.64E-06	?-?????????	TNNT2
15-76085367	15	76,085,367	C	T	0.9813	0.9732	0.9853	2.4396	0.549	8.83E-06	+???+???++?	LOC730058
2-52233271	2	52,233,271	C	T	0.9737	0.9621	0.9821	0.9237	0.2086	9.46E-06	++-+?++??++	none
rs9431720	1	232,035,150	G	A	0.6928	0.6621	0.7684	-0.2396	0.0541	9.57E-06	--------++-	DISC1
rs34445340	8	62,895,791	C	A	0.5322	0.4689	0.5755	0.1954	0.0442	9.75E-06	++++-++--+-	none
rs6875253	5	53,160,978	T	G	0.4876	0.4014	0.5841	-0.2043	0.0462	9.99E-06	--------+-+	none

Chr: chromosome number; EA: effect allele; RA: reference allele; Freq: frequency of effect allele; min/maxFreq: the minimum and maximum within cohort allele frequency; Effect: allele effect, in terms of the beta coefficient
