# Supplementary material for: Genome-Wide Association Meta-analysis of Neuropathologic Features of Alzheimer's Disease and Related Dementias
Source: PLoS Genet. 2014 Sep 4;10(9):e1004606. doi: 10.1371/journal.pgen.1004606 (PMC4154667; doi:10.1371/journal.pgen.1004606)
Supplement: Table S5 — Top association signals from the neuritic plaque ordinal phenotype. Chr: chromosome number; EA: effect allele; RA: reference allele; Freq: frequency of effect allele; min/maxFreq: the minimum and maximum within cohort allele frequency; Effect: allele effect, in terms of the beta coefficient. (PDF) [file pgen.1004606.s027.pdf]

Table S5: Top association signals from the neuritic plaque ordinal phenotype

Marker	Chr	Position	EA	RA	Freq	minFreq	maxFreq	Effect	StdErr	Pval	Direction	Gene
rs6857	19	45,392,254	T	C	0.3155	0.1473	0.401	0.9467	0.0656	3.14E-47	+++++++?	PVRL2
17-36075474	17	36,075,474	C	T	0.9661	0.9602	0.9667	1.4088	0.2906	1.25E-06	?+????+?	HNF1B
rs34487851	2	106,642,554	G	A	0.7349	0.6929	0.7581	0.297	0.0616	1.42E-06	++++++-+	none
rs34704559	10	111,296,290	G	T	0.8786	0.8564	0.9073	0.435	0.0909	1.70E-06	++++++++	none
rs7294478	12	7,266,805	G	A	0.5808	0.5352	0.6121	0.2385	0.0499	1.73E-06	++++++++	none
rs859812	8	5,593,810	T	C	0.5045	0.4874	0.5355	-0.2351	0.0498	2.35E-06	--++--+-	none
rs9406351	6	32,457,790	G	A	0.9433	0.9167	0.9559	0.8443	0.1812	3.16E-06	?++??+-+	none
rs62506864	8	52,300,770	C	T	0.9691	0.9628	0.9695	1.0201	0.2239	5.20E-06	++?+??+?	PXDNL
rs843451	2	240,330,994	G	A	0.742	0.7029	0.7483	-0.2849	0.0632	6.64E-06	--?--???	none
rs11805526	1	239,635,249	T	C	0.0213	0.0172	0.0229	-1.3355	0.2969	6.84E-06	?-?--???	none
rs9847987	3	59,418,533	T	C	0.2012	0.1905	0.2193	-0.2696	0.0602	7.53E-06	--------	none
22-21323101	22	21,323,101	A	C	0.3332	0.3141	0.3494	-0.4621	0.1036	8.27E-06	?-?+??-?	AIFM3
21-43678066	21	43,678,066	T	C	0.0108	0.0107	0.0114	-1.3434	0.3016	8.45E-06	--??????	ABCG1

Chr: chromosome number; EA: effect allele; RA: reference allele; Freq: frequency of effect allele; min/maxFreq: the minimum and maximum within cohort allele frequency; Effect: allele effect, in terms of the beta coefficient
