# Supplementary material for: Genome-Wide Association Meta-analysis of Neuropathologic Features of Alzheimer's Disease and Related Dementias
Source: PLoS Genet. 2014 Sep 4;10(9):e1004606. doi: 10.1371/journal.pgen.1004606 (PMC4154667; doi:10.1371/journal.pgen.1004606)
Supplement: Table S6 — Top association signals from the neuritic plaque case-control phenotype. Chr: chromosome number; EA: effect allele; RA: reference allele; Freq: frequency of effect allele; min/maxFreq: the minimum and maximum within cohort allele frequency; Effect: allele effect, in terms of the beta coefficient. (PDF) [file pgen.1004606.s028.pdf]

Table S6: Top association signals from the neuritic plaque case-control phenotype

Marker	Chr	Position	EA	RA	Freq	minFreq	maxFreq	Effect	StdErr	Pval	Direction	Gene
rs6857	19	45,392,254	T	C	0.2661	0.1473	0.3556	1.2932	0.1191	1.78E-27	++++++?-	PVRL2
rs62341097	4	174,094,940	A	G	0.0458	0.0233	0.0588	-1.1474	0.1975	6.00E-09	-------+	GALNT7
21-43678066	21	43,678,066	T	C	0.0108	0.0107	0.0114	-2.108	0.3658	8.00E-09	--??????	ABCG1
9-129356304	9	129,356,304	T	G	0.0172	0.0108	0.0179	-1.7657	0.3224	4.30E-08	?--?--??	none
rs17879437	19	36,642,884	C	T	0.7681	0.7547	0.7763	0.4789	0.0957	5.57E-07	++?-++?+	COX7A1
4-65287903	4	65,287,903	C	T	0.9882	0.9847	0.9899	1.6678	0.3399	9.28E-07	++??+???	none
rs9578438	13	22,729,205	T	C	0.0393	0.0367	0.0412	-1.2374	0.2537	1.08E-06	--?-????	none
3-72020152	3	72,020,152	G	T	0.9743	0.9429	0.9814	0.9748	0.2039	1.75E-06	-+-++-?+	none
rs28479400	15	99,995,884	G	A	0.9494	0.9281	0.9657	0.7864	0.1647	1.79E-06	++-+++?-	none
rs9938198	16	20,553,483	C	T	0.943	0.9269	0.9596	0.7999	0.1678	1.88E-06	+++++++-	ACSM2B
rs7294478	12	7,266,805	G	A	0.5847	0.5352	0.6121	0.3434	0.0728	2.42E-06	++++++++	none
6-14334854	6	14,334,854	C	T	0.9854	0.9759	0.989	1.5167	0.3246	2.98E-06	++?+++?+	none
rs61041336	16	58,733,162	G	A	0.8469	0.8326	0.8659	0.4512	0.0966	3.04E-06	+++++++-	none
rs34487851	2	106,642,554	G	A	0.7353	0.6929	0.771	0.4143	0.0903	4.47E-06	++++++++	none
rs8037790	15	92,542,831	A	G	0.2296	0.1981	0.258	-0.4495	0.0982	4.69E-06	------?-	SLCO3A1
3-140184142	3	140,184,142	G	A	0.9509	0.9407	0.964	0.8659	0.1908	5.67E-06	++++++-+	CLSTN2
10-3862933	10	3,862,933	T	C	0.128	0.1194	0.1458	-0.5928	0.1317	6.73E-06	----?-??	none
16-27101370	16	27,101,370	A	C	0.0587	0.046	0.0666	-0.9141	0.2051	8.32E-06	--?---?+	none
8-3743232	8	3,743,232	C	A	0.9794	0.9717	0.9869	1.0652	0.2397	8.83E-06	++?+?+--	CSMD1
8-91474673	8	91,474,673	T	C	0.0111	0.0111	0.0111	-2.7908	0.6293	9.23E-06	?-??????	none
14-23415178	14	23,415,178	G	A	0.97	0.9644	0.9792	1.3235	0.2987	9.41E-06	++?++???	none

Chr: chromosome number; EA: effect allele; RA: reference allele; Freq: frequency of effect allele; min/maxFreq: the minimum and maximum within cohort allele frequency; Effect: allele effect, in terms of the beta coefficient
