# Supplementary material for: Genome-Wide Association Meta-analysis of Neuropathologic Features of Alzheimer's Disease and Related Dementias
Source: PLoS Genet. 2014 Sep 4;10(9):e1004606. doi: 10.1371/journal.pgen.1004606 (PMC4154667; doi:10.1371/journal.pgen.1004606)
Supplement: Table S7 — Top association signals from the Lewy body disease (LBD) ordinal I phenotype. Chr: chromosome number; EA: effect allele; RA: reference allele; Freq: frequency of effect allele; min/maxFreq: the minimum and maximum within cohort allele frequency; Effect: allele effect, in terms of the beta coefficient. (PDF) [file pgen.1004606.s029.pdf]

Table S7: Top association signals from the Lewy body disease (LBD) ordinal I phenotype

Marker	Chr	Position	EA	RA	Freq	minFreq	maxFreq	Effect	StdErr	Pval	Direction	Gene
rs429358	19	45,411,941	C	T	0.732	0.6482	0.8712	-0.4997	0.0702	1.10E-12	------	APOE,LOC100129500
4-185405621	4	185,405,621	A	G	0.0524	0.0355	0.0613	0.528	0.1106	1.82E-06	++++++	none
rs1570582	9	85,910,512	C	A	0.9471	0.9259	0.9691	-0.5137	0.1082	2.07E-06	------	FRMD3
12-85076811	12	85,076,811	C	A	0.966	0.9613	0.9874	-0.7577	0.1614	2.66E-06	--+-+-	none
1-228841770	1	228,841,770	A	C	0.1068	0.0863	0.1234	0.3993	0.0851	2.71E-06	++++++	none
rs12590995	14	44,682,864	C	A	0.9191	0.9131	0.9527	-0.4494	0.0962	3.02E-06	-----+	none
rs6127813	20	55,255,054	T	C	0.2983	0.2515	0.3032	-0.2697	0.0581	3.42E-06	------	none
rs2124379	11	18,297,563	T	C	0.423	0.4075	0.4253	-0.2774	0.0598	3.49E-06	------	none
rs55770291	11	76,922,581	A	G	0.0114	0.0109	0.0163	1.6201	0.3527	4.37E-06	+++???	MYO7A
rs262741	5	165,132,974	G	A	0.5416	0.5355	0.5926	0.2447	0.0537	5.30E-06	?+++++	none
1-155733245	1	155,733,245	T	C	0.0602	0.0559	0.0833	0.7777	0.1711	5.48E-06	?++?+-	GON4L
rs28523990	7	124,436,139	T	C	0.282	0.2637	0.3198	-0.2719	0.0606	7.28E-06	-----+	none
rs133911	22	44,523,162	G	A	0.7038	0.696	0.7091	-0.3047	0.068	7.44E-06	---??-	PARVB
rs2452591	4	95,476,719	A	C	0.3832	0.3103	0.4043	-0.2693	0.0603	7.94E-06	------	PDLIM5
rs7078209	10	124,860,026	G	T	0.6751	0.646	0.7021	0.2658	0.0596	8.30E-06	++++++	none
rs2584363	8	108,158,383	T	C	0.1921	0.1682	0.2212	-0.3098	0.0696	8.45E-06	+-----	none
rs6887317	5	11,371,047	A	G	0.407	0.3885	0.4576	0.2466	0.0554	8.61E-06	?+++++	CTNND2
rs11148252	13	53,009,048	C	T	0.5913	0.5461	0.6042	-0.2351	0.0529	8.77E-06	+-----	VPS36
rs913351	9	130,033,547	G	A	0.9798	0.9775	0.9868	-0.811	0.1825	8.84E-06	--+-+-	GARNL3
15-52443375	15	52,443,375	A	G	0.0103	0.0102	0.0123	2.0434	0.4612	9.39E-06	?+???+	GNB5
rs11618716	13	53,046,836	C	T	0.5858	0.5478	0.5991	-0.2399	0.0542	9.67E-06	+-----	CKAP2

Chr: chromosome number; EA: effect allele; RA: reference allele; Freq: frequency of effect allele; min/maxFreq: the minimum and maximum within cohort allele frequency; Effect: allele effect, in terms of the beta coefficient
