# Supplementary material for: Genome-Wide Association Meta-analysis of Neuropathologic Features of Alzheimer's Disease and Related Dementias
Source: PLoS Genet. 2014 Sep 4;10(9):e1004606. doi: 10.1371/journal.pgen.1004606 (PMC4154667; doi:10.1371/journal.pgen.1004606)
Supplement: Table S8 — Top association signals from the Lewy body disease (LBD) case-control phenotype. Chr: chromosome number; EA: effect allele; RA: reference allele; Freq: frequency of effect allele; min/maxFreq: the minimum and maximum within cohort allele frequency; Effect: allele effect, in terms of the beta coefficient. (PDF) [file pgen.1004606.s030.pdf]

Table S8: Top association signals from the Lewy body disease (LBD) case-control phenotype

Marker	Chr	Position	EA	RA	Freq	minFreq	maxFreq	Effect	StdErr	Pval	Direction	Gene
rs429358	19	45,411,941	C	T	0.7344	0.6482	0.8712	-0.4905	0.0737	2.83E-11	------	APOE,LOC100129500
4-185384861	4	185,384,861	G	A	0.9531	0.9439	0.966	-0.659	0.1343	9.32E-07	----+-	IRF2
rs6127813	20	55,255,054	T	C	0.2984	0.2529	0.3032	-0.282	0.0597	2.31E-06	------	none
rs7078209	10	124,860,026	G	T	0.6744	0.6409	0.7021	0.2812	0.0611	4.10E-06	++-+++	none
rs727505	7	124,462,081	A	G	0.2876	0.2689	0.3287	-0.2807	0.0613	4.62E-06	------	none
rs2803904	14	38,641,706	C	T	0.5791	0.5434	0.5935	0.2574	0.0563	4.90E-06	++++++	none
rs11024598	11	18,291,736	G	A	0.4743	0.4644	0.5181	0.262	0.0585	7.60E-06	++++++	none
2-228891013	2	228,891,013	G	A	0.9785	0.97	0.9867	-1.1951	0.268	8.22E-06	----?-	SPHKAP
rs7132746	12	86,259,458	C	T	0.8751	0.8519	0.9059	-0.3439	0.0775	9.15E-06	--+--+	none

Chr: chromosome number; EA: effect allele; RA: reference allele; Freq: frequency of effect allele; min/maxFreq: the minimum and maximum within cohort allele frequency; Effect: allele effect, in terms of the beta coefficient
