# Supplementary material for: Genome-Wide Association Meta-analysis of Neuropathologic Features of Alzheimer's Disease and Related Dementias
Source: PLoS Genet. 2014 Sep 4;10(9):e1004606. doi: 10.1371/journal.pgen.1004606 (PMC4154667; doi:10.1371/journal.pgen.1004606)
Supplement: Table S9 — Top association signals from the Lewy body disease (LBD) ordinal II phenotype. Chr: chromosome number; EA: effect allele; RA: reference allele; Freq: frequency of effect allele; min/maxFreq: the minimum and maximum within cohort allele frequency; Effect: allele effect, in terms of the beta coefficient. (PDF) [file pgen.1004606.s031.pdf]

Table S9: Top association signals from the Lewy body disease (LBD) ordinal II phenotype

Marker	Chr	Position	EA	RA	Freq	minFreq	maxFreq	Effect	StdErr	Pval	Direction	Gene
rs429358	19	45,411,941	C	T	0.7347	0.6482	0.8712	-0.508	0.0735	4.87E-12	------	APOE,LOC100129500
4-185384861	4	185,384,861	G	A	0.9531	0.9439	0.9656	-0.6523	0.1335	1.02E-06	----+-	IRF2
rs6127813	20	55,255,054	T	C	0.2985	0.253	0.3032	-0.2843	0.0594	1.70E-06	------	none
rs7132746	12	86,259,458	C	T	0.8751	0.8519	0.9048	-0.3623	0.0769	2.47E-06	--+--+	none
rs7078209	10	124,860,026	G	T	0.6747	0.6455	0.7021	0.2772	0.0609	5.25E-06	++++++	none
rs12590995	14	44,682,864	C	A	0.9192	0.9131	0.9527	-0.4532	0.0997	5.47E-06	------	none
rs727505	7	124,462,081	A	G	0.2875	0.2689	0.3287	-0.2777	0.0612	5.70E-06	------	none
2-228891013	2	228,891,013	G	A	0.9785	0.9696	0.9867	-1.1721	0.2598	6.45E-06	----?-	SPHKAP
rs11739210	5	119,004,300	C	T	0.9824	0.9749	0.9869	-1.1285	0.2533	8.40E-06	+-+--?	none
rs1570582	9	85,910,512	C	A	0.9476	0.9259	0.9691	-0.502	0.113	8.83E-06	-----+	FRMD3
rs11024598	11	18,291,736	G	A	0.4741	0.4644	0.5181	0.2593	0.0585	9.31E-06	++++++	none

Chr: chromosome number; EA: effect allele; RA: reference allele; Freq: frequency of effect allele; min/maxFreq: the minimum and maximum within cohort allele frequency; Effect: allele effect, in terms of the beta coefficient
