# Supplementary material for: Genome-Wide Association Meta-analysis of Neuropathologic Features of Alzheimer's Disease and Related Dementias
Source: PLoS Genet. 2014 Sep 4;10(9):e1004606. doi: 10.1371/journal.pgen.1004606 (PMC4154667; doi:10.1371/journal.pgen.1004606)
Supplement: Table S10 — Top association signals from the vascular brain injury (VBI) case-control phenotype. Chr: chromosome number; EA: effect allele; RA: reference allele; Freq: frequency of effect allele; min/maxFreq: the minimum and maximum within cohort allele frequency; Effect: allele effect, in terms of the beta coefficient. (PDF) [file pgen.1004606.s032.pdf]

Table S10: Top association signals from the vascular brain injury (VBI) case-control phenotype

Marker	Chr	Position	EA	RA	Freq	minFreq	maxFreq	Effect	StdErr	Pval	Direction	Gene
rs11769293	7	28,911,807	T	C	0.0291	0.0233	0.0398	1.0518	0.2179	1.38E-06	++++?	none
rs11144781	9	78,793,421	T	G	0.0316	0.0228	0.038	0.8393	0.1793	2.87E-06	+++-+	PCSK5
10-106180121	10	106,180,121	G	T	0.9892	0.9856	0.9895	-2.5402	0.5447	3.11E-06	--+??	CCDC147
rs34660913	11	13,158,010	T	C	0.0931	0.0784	0.1065	-0.6319	0.1355	3.13E-06	---?-	none
rs7596264	2	144,168,635	G	A	0.8696	0.865	0.9259	0.4449	0.0978	5.40E-06	++++-	ARHGAP15
rs9685722	4	16,407,135	T	C	0.1213	0.0722	0.1388	-0.4804	0.1059	5.75E-06	----+	none
rs6041428	20	12,506,345	G	A	0.9842	0.9737	0.987	-1.256	0.2777	6.12E-06	+--??	none
rs7048146	9	112,299,322	T	C	0.3898	0.3602	0.4477	-0.2753	0.061	6.31E-06	-----	none
3-66952276	3	66,952,276	C	T	0.9842	0.9839	0.9873	-1.2693	0.2842	7.93E-06	+--??	none
rs11602337	11	25,113,781	T	C	0.2923	0.2764	0.3059	-0.2943	0.066	8.20E-06	-----	none
rs62174474	2	188,180,409	C	T	0.9439	0.9027	0.9499	-0.7084	0.159	8.35E-06	---?-	none
7-134674232	7	134,674,232	A	G	0.0219	0.0201	0.068	1.0975	0.2466	8.56E-06	?+??+	AGBL3
rs7000623	8	5,741,723	A	C	0.4026	0.3805	0.4445	-0.2921	0.0661	9.88E-06	--+--	none

Chr: chromosome number; EA: effect allele; RA: reference allele; Freq: frequency of effect allele; min/maxFreq: the minimum and maximum within cohort allele frequency; Effect: allele effect, in terms of the beta coefficient
