# Supplementary material for: Genome-Wide Association Meta-analysis of Neuropathologic Features of Alzheimer's Disease and Related Dementias
Source: PLoS Genet. 2014 Sep 4;10(9):e1004606. doi: 10.1371/journal.pgen.1004606 (PMC4154667; doi:10.1371/journal.pgen.1004606)
Supplement: Table S11 — Top association signals from the vascular brain injury (VBI) ordinal phenotype. Chr: chromosome number; EA: effect allele; RA: reference allele; Freq: frequency of effect allele; min/maxFreq: the minimum and maximum within cohort allele frequency; Effect: allele effect, in terms of the beta coefficient. (PDF) [file pgen.1004606.s033.pdf]

Table S11: Top association signals from the vascular brain injury (VBI) ordinal phenotype

Marker	Chr	Position	EA	RA	Freq	minFreq	maxFreq	Effect	StdErr	Pval	Direction	Gene
rs7048146	9	112,299,322	T	C	0.3891	0.3602	0.4596	-0.2933	0.0588	6.10E-07	------	none
rs11144781	9	78,793,421	T	G	0.0318	0.0228	0.0434	0.7998	0.167	1.67E-06	++-+-+	PCSK5
3-66952276	3	66,952,276	C	T	0.9841	0.9839	0.9873	-1.2736	0.266	1.69E-06	+-?-??	none
10-106180121	10	106,180,121	G	T	0.9893	0.9856	0.9895	-2.0531	0.4378	2.74E-06	--?+??	CCDC147
rs34660913	11	13,158,010	T	C	0.0922	0.0666	0.1065	-0.6007	0.1319	5.24E-06	--+-?-	none
7-134674232	7	134,674,232	A	G	0.0219	0.0201	0.0785	1.0385	0.2295	6.02E-06	?+???+	AGBL3
rs2018293	22	26,704,561	T	C	0.185	0.1538	0.2401	0.3091	0.0692	8.01E-06	++-++-	SEZ6L
rs11769293	7	28,911,807	T	C	0.0289	0.0233	0.0398	0.9011	0.202	8.18E-06	++-++?	none
8-80222312	8	80,222,312	T	C	0.1612	0.1163	0.1887	-0.3494	0.0787	9.00E-06	-----+	none
rs13331544	16	25,779,378	G	A	0.5781	0.4655	0.6283	0.2622	0.0592	9.60E-06	++-+++	none
3-108271501	3	108,271,501	T	C	0.0129	0.0127	0.0265	1.2639	0.2858	9.78E-06	++???+	KIAA1524

Chr: chromosome number; EA: effect allele; RA: reference allele; Freq: frequency of effect allele; min/maxFreq: the minimum and maximum within cohort allele frequency; Effect: allele effect, in terms of the beta coefficient
