# Supplementary material for: Genome-Wide Association Meta-analysis of Neuropathologic Features of Alzheimer's Disease and Related Dementias
Source: PLoS Genet. 2014 Sep 4;10(9):e1004606. doi: 10.1371/journal.pgen.1004606 (PMC4154667; doi:10.1371/journal.pgen.1004606)
Supplement: Table S12 — Top association signals from the hippocampal sclerosis (HS) case-control phenotype. Chr: chromosome number; EA: effect allele; RA: reference allele; Freq: frequency of effect allele; min/maxFreq: the minimum and maximum within cohort allele frequency; Effect: allele effect, in terms of the beta coefficient. (PDF) [file pgen.1004606.s034.pdf]

Table S12: Top association signals from the hippocampal sclerosis (HS) case-control phenotype

Marker	Chr	Position	EA	RA	Freq	minFreq	maxFreq	Effect	StdErr	Pval	Direction	Gene
18-6434981	18	6,434,981	A	C	0.0104	0.0104	0.0104	2.5601	0.4684	4.60E-08	?+??	none
rs9637454	3	178,257,562	A	G	0.2791	0.27	0.2819	0.5006	0.0929	7.10E-08	++++	KCNMB2
rs34132583	4	146,360,757	A	G	0.0416	0.0329	0.0512	0.9231	0.1793	2.64E-07	+++-	none
3-16865013	3	16,865,013	T	G	0.0165	0.0126	0.0245	1.3092	0.2653	8.01E-07	?++-	none
rs4589573	16	30,268,781	T	C	0.0152	0.0122	0.0154	2.023	0.4141	1.03E-06	++?-	none
rs8074980	17	56,010,960	A	G	0.1716	0.1538	0.1937	0.5238	0.1077	1.14E-06	+++-	none
rs11241261	5	113,395,481	T	C	0.1491	0.1326	0.1553	0.5443	0.1135	1.64E-06	++++	none
rs3007243	13	105,806,954	G	A	0.959	0.9425	0.9683	-0.8386	0.1752	1.69E-06	----	none
rs12950363	17	35,015,902	T	G	0.0749	0.0704	0.0767	0.9201	0.1931	1.89E-06	?+++	none
11-20955053	11	20,955,053	G	A	0.9555	0.9451	0.9593	-0.8951	0.1898	2.40E-06	---?	NELL1
20-38725000	20	38,725,000	C	T	0.9818	0.977	0.9832	-1.7275	0.3666	2.45E-06	---?	none
rs28671666	7	12,184,430	A	G	0.2128	0.1779	0.2244	0.5396	0.1157	3.09E-06	++++	none
6-149652735	6	149,652,735	T	C	0.0109	0.0108	0.0116	1.7356	0.373	3.27E-06	?++?	none
rs10906824	10	15,128,969	A	G	0.033	0.0244	0.0383	0.9719	0.2095	3.50E-06	+++-	ACBD7
16-59161124	16	59,161,124	G	A	0.9704	0.9696	0.9821	-1.0064	0.218	3.91E-06	----	none
rs12316703	12	118,840,457	G	A	0.9171	0.9099	0.9201	-0.6574	0.1427	4.09E-06	----	SUDS3
3-192820385	3	192,820,385	A	G	0.0177	0.0168	0.0182	1.5944	0.3466	4.22E-06	++++	none
2-62486295	2	62,486,295	C	T	0.8097	0.7916	0.8479	-0.585	0.1274	4.39E-06	---+	none
3-145097436	3	145,097,436	A	G	0.0411	0.0363	0.0425	1.1395	0.2486	4.59E-06	?++?	none
rs12498806	4	127,363,097	C	T	0.9832	0.9813	0.9835	-1.2967	0.2836	4.81E-06	--??	none
16-20899251	16	20,899,251	C	T	0.9721	0.9618	0.9796	-1.2586	0.2757	4.98E-06	----	DCUN1D3
11-126602487	11	126,602,487	G	A	0.9828	0.9822	0.9875	-1.6129	0.3589	6.97E-06	?-+-	KIRREL3
rs7235840	18	73,725,200	T	C	0.1476	0.1303	0.16	0.5571	0.124	7.06E-06	++++	none
8-288884	8	288,884	A	G	0.0406	0.0279	0.046	0.9309	0.208	7.64E-06	+++-	none
rs62277617	4	7,459,875	G	A	0.9132	0.9022	0.9507	-0.7386	0.1651	7.72E-06	----	SORCS2
12-127979137	12	127,979,137	T	C	0.0146	0.013	0.0151	1.635	0.3656	7.75E-06	?+++	none
16-82453134	16	82,453,134	T	C	0.0179	0.0147	0.0213	1.4961	0.3354	8.20E-06	++++	none
3-130938261	3	130,938,261	T	C	0.0219	0.0213	0.0241	1.3424	0.3014	8.42E-06	?++-	NEK11
12-3184006	12	3,184,006	T	C	0.0383	0.0292	0.0466	1.0377	0.2335	8.87E-06	?+++	none
6-147224959	6	147,224,959	T	C	0.0125	0.0125	0.0143	2.1229	0.4794	9.51E-06	-+??	none
8-63185819	8	63,185,819	A	C	0.0262	0.0119	0.0321	1.2774	0.2888	9.72E-06	++++	NKAIN3
rs5992977	22	18,530,144	T	C	0.0195	0.0185	0.0257	1.2384	0.2803	9.99E-06	++++	none

Chr: chromosome number; EA: effect allele; RA: reference allele; Freq: frequency of effect allele; min/maxFreq: the minimum and maximum within cohort allele frequency; Effect: allele effect, in terms of the beta coefficient
