# Supplementary material for: Genome-Wide Association Meta-analysis of Neuropathologic Features of Alzheimer's Disease and Related Dementias
Source: PLoS Genet. 2014 Sep 4;10(9):e1004606. doi: 10.1371/journal.pgen.1004606 (PMC4154667; doi:10.1371/journal.pgen.1004606)
Supplement: Table S13 — Top association signals from the cerebral amyloid angiopathy (CAA) case-control phenotype. Chr: chromosome number; EA: effect allele; RA: reference allele; Freq: frequency of effect allele; min/maxFreq: the minimum and maximum within cohort allele frequency; Effect: allele effect, in terms of the beta coefficient. (PDF) [file pgen.1004606.s035.pdf]

Table S13: Top association signals from the cerebral amyloid angiopathy (CAA) case-control phenotype

Marker	Chr	Position	EA	RA	Freq	minFreq	maxFreq	Effect	StdErr	Pval	Direction	Gene
rs6857	19	45,392,254	T	C	0.3429	0.1511	0.3997	0.6708	0.0709	2.92E-21	++++?+	PVRL2
rs16989979	4	34,545,346	T	G	0.2408	0.2166	0.2766	-0.3325	0.0697	1.82E-06	--+--+	none
rs17014760	4	130,340,880	A	G	0.2465	0.1702	0.2748	0.3262	0.0724	6.64E-06	+++++-	none
rs1116547	5	112,680,337	T	C	0.1269	0.0649	0.1594	-0.396	0.088	6.82E-06	---++-	MCC
rs11075138	16	12,723,811	G	A	0.9067	0.9019	0.9589	-0.5115	0.1145	7.95E-06	------	none
rs7817714	8	103,048,810	C	T	0.859	0.848	0.8901	-0.4448	0.0999	8.52E-06	--?---	NCALD
7-18698331	7	18,698,331	G	T	0.9733	0.9664	0.9775	-1.1568	0.2606	9.06E-06	--?--?	HDAC9
rs6921741	6	21,864,168	A	C	0.1491	0.0517	0.1763	0.4047	0.0916	9.91E-06	+++++-	none

Chr: chromosome number; EA: effect allele; RA: reference allele; Freq: frequency of effect allele; min/maxFreq: the minimum and maximum within cohort allele frequency; Effect: allele effect, in terms of the beta coefficient
