# Supplementary material for: Genome-Wide Association Meta-analysis of Neuropathologic Features of Alzheimer's Disease and Related Dementias
Source: PLoS Genet. 2014 Sep 4;10(9):e1004606. doi: 10.1371/journal.pgen.1004606 (PMC4154667; doi:10.1371/journal.pgen.1004606)
Supplement: Table S14 — Association of common AD risk variants with core AD neuropathologic features. Bold text indicates p-values meeting an alpha = 0.05 threshold, uncorrected for multiple testing. (PDF) [file pgen.1004606.s036.pdf]

Table S14: Association of common AD risk variants with core AD neuropathologic features

						IGAP Paper (CC)			NFT Braak (Ordinal-I)		
**Type**	**SNP**	**GENE**	**CHR**	**POS**	**Major/Minor**	**MAF**	**OR**	**p-value**	**MAF**	**OR**	**p-value**
Known	rs6656401	CR1	1	207,692,049	G/A	0.197	1.18	5.7E-24	0.204	1.07	1.80E-01
Known	rs6733839	BIN1	2	127,892,810	C/T	0.409	1.22	6.9E-44	0.439	1.19	**6.30E-05**
Known	rs10948363	CD2AP	6	47,487,762	A/G	0.266	1.10	5.2E-11	0.280	1.08	7.90E-02
Known	rs11771145	EPHA1	7	143,110,762	G/A	0.338	0.90	1.1E-13	0.333	0.94	1.10E-01
Known	rs9331896	CLU	8	27,467,686	T/C	0.379	0.86	2.8E-25	0.373	0.91	**2.90E-02**
Known	rs983392	MS4A6A	11	59,923,508	A/G	0.403	0.90	6.1E-16	0.390	0.94	9.80E-02
Known	rs10792832	PICALM	11	85,867,875	G/A	0.358	0.87	9.3E-26	0.350	0.89	**2.70E-03**
Known	rs4147929	ABCA7	19	1,063,443	G/A	0.190	1.15	1.1E-15	0.188	1.12	**3.00E-02**
Known	rs3865444	CD33	19	51,727,962	C/A	0.307	0.94	3.0E-06	0.301	0.92	6.30E-02
New D	rs9271192	HLA-DRB5/HLA-DRB	6	32,578,530	A/C	0.276	1.11	2.9E-12	0.285	1.06	2.30E-01
New D	rs28834970	PTK2B	8	27,195,121	T/C	0.366	1.10	7.4E-14	0.372	0.99	8.60E-01
New D	rs11218343	SORL1	11	121,435,587	T/C	0.039	0.77	9.7E-15	0.041	0.83	5.70E-02
New D	rs10498633	SLC24A4/RIN3	14	92,926,952	G/T	0.217	0.91	5.5E-09	0.212	0.93	9.00E-02
New D	rs8093731	DSG2	18	29,088,958	C/T	0.017	0.73	1.0E-04	0.013	0.80	4.70E-01
New D&R	rs35349669	INPP5D	2	234,068,476	C/T	0.488	1.08	3.2E-08	0.500	1.01	7.30E-01
New D&R	rs190982	MEF2C	5	88,223,420	A/G	0.408	0.93	3.2E-08	0.415	0.93	9.30E-02
New D&R	rs2718058	NME8	7	37,841,534	A/G	0.373	0.93	4.8E-09	0.359	1.03	4.40E-01
New D&R	rs1476679	ZCWPW1	7	100,004,446	T/C	0.287	0.91	5.6E-10	0.280	0.94	1.40E-01
New D&R	rs10838725	CELF1	11	47,557,871	T/C	0.316	1.08	1.1E-08	0.316	1.05	2.80E-01
New D&R	rs17125944	FERMT2	14	53,400,629	T/C	0.092	1.14	7.9E-09	0.096	1.13	6.20E-02
New D&R	rs7274581	CASS4	20	55,018,260	T/C	0.083	0.88	2.5E-08	0.074	0.82	**7.00E-03**

Bold text indicates p-values meeting an alpha=0.05 threshold, uncorrected for multiple testing

NFT Braak (Ordinal-II)			Neuritic Plaque (Ordinal)			Neuritic Plaque (CC)		
**MAF**	**OR**	**p-value**	**MAF**	**OR**	**p-value**	**MAF**	**OR**	**p-value**
0.202	1.10	1.00E-01	0.202	1.02	7.50E-01	0.192	0.97	7.30E-01
0.437	1.25	**7.80E-06**	0.433	1.28	**1.10E-05**	0.422	1.30	**1.90E-03**
0.279	1.06	2.20E-01	0.273	1.08	1.80E-01	0.268	1.18	**4.20E-02**
0.334	0.90	**2.60E-02**	0.334	0.94	2.40E-01	0.337	0.95	5.00E-01
0.373	0.94	1.70E-01	0.375	0.92	1.20E-01	0.377	0.89	1.40E-01
0.391	0.92	6.20E-02	0.395	0.91	8.10E-02	0.404	0.84	**1.90E-02**
0.350	0.86	**1.20E-03**	0.349	0.83	**2.40E-04**	0.353	0.85	**2.90E-02**
0.188	1.14	**2.80E-02**	0.186	1.15	**4.30E-02**	0.188	1.32	**1.10E-02**
0.300	0.94	1.80E-01	0.298	0.87	**1.10E-02**	0.296	0.87	9.30E-02
0.286	1.07	1.80E-01	0.282	1.03	5.60E-01	0.282	1.03	6.90E-01
0.373	1.02	6.00E-01	0.373	1.09	1.10E-01	0.381	1.09	2.20E-01
0.041	0.78	**1.50E-02**	0.039	0.83	1.30E-01	0.041	0.86	4.10E-01
0.213	0.94	2.20E-01	0.214	1.01	8.00E-01	0.219	1.12	2.00E-01
0.013	0.92	7.60E-01	0.015	2.24	1.20E-01	0.013	0.83	8.80E-01
0.498	1.00	9.50E-01	0.496	0.98	6.50E-01	0.485	1.01	8.90E-01
0.414	0.90	**3.10E-02**	0.415	0.91	6.80E-02	0.407	0.78	**1.30E-03**
0.358	1.06	1.90E-01	0.357	0.94	2.10E-01	0.355	0.96	5.90E-01
0.281	0.90	**3.80E-02**	0.281	0.91	9.50E-02	0.285	0.94	4.00E-01
0.314	1.03	5.70E-01	0.316	1.01	8.30E-01	0.311	1.00	9.70E-01
0.096	1.09	2.70E-01	0.095	1.22	**2.50E-02**	0.093	1.21	1.60E-01
0.074	0.77	**1.80E-03**	0.077	0.85	9.20E-02	0.076	0.64	**3.60E-04**
