# Supplementary material for: Genome-Wide Association Meta-analysis of Neuropathologic Features of Alzheimer's Disease and Related Dementias
Source: PLoS Genet. 2014 Sep 4;10(9):e1004606. doi: 10.1371/journal.pgen.1004606 (PMC4154667; doi:10.1371/journal.pgen.1004606)
Supplement: Table S15 — Association of common AD risk variants with coincident neuropathologic features. Bold text indicates p-values meeting an alpha = 0.05 threshold; uncorrected for multiple testing. (PDF) [file pgen.1004606.s037.pdf]

Table S15: Association of common AD risk variants with coincident neuropathologic features

						IGAP Paper (CC)			Lewy Body (Ordinal-I)			Le
Type	SNP	GENE	CHR	POS	Major/Mino	MAF	OR	p-value	MAF	OR	p-value	MAF
Known	rs6656401	CR1	1	207,692,049	G/A	0.197	1.18	5.7E-24	0.204	0.98	8.00E-01	0.203
Known	rs6733839	BIN1	2	127,892,810	C/T	0.409	1.22	6.9E-44	0.441	1.09	1.20E-01	0.441
Known	rs10948363	CD2AP	6	47,487,762	A/G	0.266	1.10	5.2E-11	0.276	1.00	9.90E-01	0.275
Known	rs11771145	EPHA1	7	143,110,762	G/A	0.338	0.90	1.1E-13	0.334	1.05	3.90E-01	0.334
Known	rs9331896	CLU	8	27,467,686	T/C	0.379	0.86	2.8E-25	0.372	0.94	2.50E-01	0.372
Known	rs983392	MS4A6A	11	59,923,508	A/G	0.403	0.90	6.1E-16	0.390	1.01	8.70E-01	0.390
Known	rs10792832	PICALM	11	85,867,875	G/A	0.358	0.87	9.3E-26	0.348	1.01	7.90E-01	0.348
Known	rs4147929	ABCA7	19	1,063,443	G/A	0.190	1.15	1.1E-15	0.187	0.98	8.00E-01	0.186
Known	rs3865444	CD33	19	51,727,962	C/A	0.307	0.94	3.0E-06	0.301	1.01	9.10E-01	0.301
New D	rs9271192	-DRB5/HLA-	6	32,578,530	A/C	0.276	1.11	2.9E-12	0.283	1.05	3.90E-01	0.282
New D	rs28834970	PTK2B	8	27,195,121	T/C	0.366	1.10	7.4E-14	0.364	0.98	7.40E-01	0.364
New D	rs11218343	SORL1	11	121,435,587	T/C	0.039	0.77	9.7E-15	0.036	1.31	**4.90E-02**	0.036
New D	rs10498633	LC24A4/RIN	14	92,926,952	G/T	0.217	0.91	5.5E-09	0.211	0.98	7.50E-01	0.211
New D	rs8093731	DSG2	18	29,088,958	C/T	0.017	0.73	1.0E-04	0.011	0.85	6.10E-01	0.011
New D&R	rs35349669	INPP5D	2	234,068,476	C/T	0.488	1.08	3.2E-08	0.505	1.03	5.70E-01	0.505
New D&R	rs190982	MEF2C	5	88,223,420	A/G	0.408	0.93	3.2E-08	0.417	0.90	6.40E-02	0.417
New D&R	rs2718058	NME8	7	37,841,534	A/G	0.373	0.93	4.8E-09	0.352	1.01	9.20E-01	0.352
New D&R	rs1476679	ZCWPW1	7	100,004,446	T/C	0.287	0.91	5.6E-10	0.277	1.03	5.90E-01	0.278
New D&R	rs10838725	CELF1	11	47,557,871	T/C	0.316	1.08	1.1E-08	0.318	1.00	9.80E-01	0.317
New D&R	rs17125944	FERMT2	14	53,400,629	T/C	0.092	1.14	7.9E-09	0.094	1.05	6.10E-01	0.094
New D&R	rs7274581	CASS4	20	55,018,260	T/C	0.083	0.88	2.5E-08	0.074	1.05	6.40E-01	0.074

Bold text indicates p-values meeting an alpha=0.05 threshold, uncorrected for multiple testing

wy Body (CC)		Lewy Body (Ordinal-II)			Vascular Brain Injury (CC)			Vascular Brain Injury (Ordinal)			Medial Temporal Scle	
OR	p-value	MAF	OR	p-value	MAF	OR	p-value	MAF	OR	p-value	MAF	OR
0.99	8.60E-01	0.203	0.99	8.40E-01	0.217	1.00	9.80E-01	0.217	0.99	8.90E-01	0.212	1.18
1.08	1.70E-01	0.441	1.10	1.10E-01	0.441	0.89	5.60E-02	0.441	0.91	1.50E-01	0.439	1.08
1.00	1.00E+00	0.276	0.99	9.00E-01	0.282	1.03	6.20E-01	0.282	1.05	4.30E-01	0.276	0.96
1.04	4.90E-01	0.334	1.04	5.10E-01	0.337	0.93	2.10E-01	0.337	0.91	1.20E-01	0.337	1.09
0.93	1.90E-01	0.372	0.92	1.60E-01	0.375	1.03	6.10E-01	0.375	1.03	6.00E-01	0.379	0.99
1.01	8.80E-01	0.390	1.01	8.30E-01	0.390	1.06	3.30E-01	0.390	1.06	3.10E-01	0.394	0.95
1.01	9.00E-01	0.348	1.01	9.20E-01	0.348	0.94	3.10E-01	0.347	0.97	5.50E-01	0.350	1.16
0.99	9.20E-01	0.186	1.00	9.80E-01	0.182	1.10	2.00E-01	0.182	1.08	3.10E-01	0.190	0.99
0.99	8.70E-01	0.301	1.00	9.50E-01	0.306	0.93	2.20E-01	0.306	0.95	3.90E-01	0.300	0.97
1.05	4.10E-01	0.283	1.05	4.00E-01	0.282	0.96	4.90E-01	0.283	0.96	5.10E-01	0.282	1.04
1.00	9.60E-01	0.364	0.99	8.80E-01	0.366	0.96	5.50E-01	0.367	0.95	4.20E-01	0.362	0.78
1.31	5.10E-02	0.036	1.32	**5.00E-02**	0.035	1.14	4.10E-01	0.035	1.13	4.30E-01	0.036	0.93
0.98	7.80E-01	0.211	0.97	6.20E-01	0.209	1.06	4.30E-01	0.211	1.08	2.60E-01	0.211	1.08
0.95	8.80E-01	0.011	0.94	8.40E-01	0.013	1.41	6.80E-01	0.015	1.09	8.90E-01	0.012	0.89
1.05	4.00E-01	0.505	1.04	5.10E-01	0.504	0.90	8.00E-02	0.502	0.93	2.00E-01	0.501	0.87
0.88	**2.20E-02**	0.417	0.88	**2.70E-02**	0.420	1.00	9.70E-01	0.420	0.98	7.80E-01	0.420	1.06
1.01	8.80E-01	0.352	1.01	7.90E-01	0.357	1.12	5.60E-02	0.356	1.12	**4.90E-02**	0.349	1.10
1.05	4.30E-01	0.278	1.04	5.30E-01	0.275	1.00	9.50E-01	0.275	0.99	8.20E-01	0.274	0.94
0.98	7.20E-01	0.317	0.97	6.30E-01	0.321	1.06	3.80E-01	0.320	1.05	4.50E-01	0.319	0.84
1.06	5.40E-01	0.094	1.07	4.90E-01	0.099	0.89	2.40E-01	0.099	0.91	3.40E-01	0.093	1.12
1.04	6.90E-01	0.074	1.03	8.00E-01	0.077	1.13	2.80E-01	0.078	1.06	5.70E-01	0.078	1.12

erosis (CC)	Amyloid Angiopathy (CC)		
p-value	MAF	OR	p-value
1.10E-01	0.210	1.03	6.70E-01
4.30E-01	0.444	1.02	7.10E-01
6.90E-01	0.283	0.98	8.10E-01
3.80E-01	0.333	1.07	2.70E-01
8.90E-01	0.376	0.94	3.80E-01
5.90E-01	0.389	0.97	6.50E-01
1.00E-01	0.349	0.96	4.70E-01
9.50E-01	0.186	1.06	4.70E-01
7.60E-01	0.305	0.94	3.10E-01
7.20E-01	0.279	0.97	7.20E-01
**9.40E-03**	0.362	0.90	1.10E-01
7.80E-01	0.036	0.98	8.90E-01
4.60E-01	0.210	1.00	9.60E-01
8.00E-01	0.011	0.82	5.40E-01
1.40E-01	0.504	0.97	6.70E-01
5.60E-01	0.424	0.98	8.00E-01
2.80E-01	0.347	1.04	5.30E-01
5.60E-01	0.278	1.07	3.10E-01
6.70E-02	0.323	0.94	3.50E-01
4.50E-01	0.097	0.91	3.60E-01
4.70E-01	0.077	1.07	5.70E-01
