# Supplementary material for: Genome-Wide Association Meta-analysis of Neuropathologic Features of Alzheimer's Disease and Related Dementias
Source: PLoS Genet. 2014 Sep 4;10(9):e1004606. doi: 10.1371/journal.pgen.1004606 (PMC4154667; doi:10.1371/journal.pgen.1004606)
Supplement: Table S16 — Cohort contact information. ACT: Adult Changes in Thought Study; ADC: Alzheimer's Disease Center; MAYO: Mayo Clinic Alzheimer's Disease Research Center; MBB: University of Miami Brain Endowment Bank; NIA-LOAD: National Institute on Aging Late–Onset Alzheimer's Disease Family Study; OHSU: Oregon Health & Science University Alzheimer's Disease Center; ROSMAP: Religious Orders Study and Memory and Aging Project; TGEN: Translational Genomics Research Institute; UM/VU/MSSM: University of Miami Hussman Institute for Human Genomics/Vanderbilt University Center for Human Genetics Research/Mount Sinai School of Medicine; UP: University of Pittsburgh Alzheimer's Disease Research Center; ADGC: Alzheimer's Disease Genetics Consortium; dbGAP: database of genotypes and phenotypes; eMERGE: electronic medical records and genomics; NACC: National Alzheimer's Coordinating Center; NCRAD: National Cell Repository for Alzheimer's Disease. (PDF) [file pgen.1004606.s038.pdf]

Table S16: Cohort contact information

Cohort	Available through request on dbGAP?	Study website	Study contact (study email)
ACT	No	(ACT) http://www.grouphealthresearch.org/capabilities/clinic/clin_std.html#act<br>(eMERGE) https://www.mc.vanderbilt.edu/victr/dcc/projects/acc/index.php/Main_Page	Eric B. Larson, MD, MPH, MACP (larson.e@ghc.org)
ADCs	No	(NACC) https://www.alz.washington.edu/<br>(NCRAD) http://ncrad.iu.edu/	(NACC) Walter Kukull, PhD (naccmail@uw.edu)<br>(NCRAD) Tatiana Foroud, PhD (alzstudy@iupui.edu)
MAYO	No	http://mayoresearch.mayo.edu/mayo/research/alzheimers_center/	Steven G. Younkin, MD, PhD (younkin.steven@mayo.edu)
MBB	No	http://brainbank.med.miami.edu/	Deborah Mash (dmash@med.miami.edu)
NIA-LOAD	Yes	http://www.niageneticsinitiative.org/	Richard Mayeux, MD, MSc (rpm2@columbia.edu)
OHSU	No	http://www.ohsu.edu/xd/research/centers-institutes/neurology/alzheimers/research/data-tissue/biomarkers-genetics.cfm	Patricia L. Kramer (kramer@ohsu.edu)
ROSMAP	Yes	https://www.radc.rush.edu/res/ext/docs/Rush_ADCC_Data_Sharing.html	David Bennett, MD (dbennett@rush.edu)<br>Data Sharing: Gregory Klein (Gregory_Klein@rush.edu)
TGEN2	No	http://www.tgen.org/research/index.cfm?pageid=1065	Eric Reiman, MD (eric_reiman@tgen.org)
UM/VU/MSSM	No	http://www.hihg.org/ (Contact Directly)	Margaret A. Pericak-Vance, PhD (mpericak@med.miami.edu)
UP	No	http://www.adrc.pitt.edu/neurocore.asp (Contact Directly)	M. Ilyas Kamboh, PhD (kamboh@pitt.edu)
**ADGC Neuropath Study**		(Main Study) http://alois.med.upenn.edu/adgc/<br>(Dataset Access) http://www.niageneticsdata.org/	Gerard Schellenberg (gerardsc@mail.med.upenn.edu),<br>Data Sharing: Li-San Wang (lswang@mail.med.penn.edu)

ACT: Adult Changes in Thought Study; ADC: Alzheimer’s Disease Center; MAYO: Mayo Clinic Alzheimer’s Disease Research Center; MBB: University of Miami Brain Endowment Bank; NIA-LOAD: National Institute on Aging Late-Onset Alzheimer’s Disease Family Study; OHSU: Oregon Health & Science University Alzheimer’s Disease Center; ROSMAP: Religious Orders Study and Memory and Aging Project; TGEN: Translational Genomics Research Institute; UM/VU/MSSM: University of Miami Hussman Institute for Human Genomics/Vanderbilt University Center for Human Genetics Research/Mount Sinai School of Medicine; UP: University of Pittsburgh Alzheimer’s Disease Research Center; ADGC: Alzheimer’s Disease Genetics Consortium; dbGAP: database of genotypes and phenotypes; eMERGE: electronic medical records and genomics; NACC: National Alzheimer’s Coordinating Center; NCRAD: National Cell Repository for Alzheimer's Disease.
