# Supplementary material for: Genome-Wide Association Meta-analysis of Neuropathologic Features of Alzheimer's Disease and Related Dementias
Source: PLoS Genet. 2014 Sep 4;10(9):e1004606. doi: 10.1371/journal.pgen.1004606 (PMC4154667; doi:10.1371/journal.pgen.1004606)
Supplement: Table S17 — Descriptive statistics of cohorts. AAO: age at onset; AAD: age at death; AAE: age at exam; SD: standard deviation. APOE: relative frequency of APOE genotypes where * represents E2 or E3. Cohorts: ACT: Adult Changes in Thought Study; ADC: Alzheimer's Disease Center; TGEN: Translational Genomics Research Institute; LOAD: National Institute on Aging Late-Onset Alzheimer's Disease Family Study; MAYO: Mayo Clinic Alzheimer's Disease Research Center; ROSMAP: Religious Orders Study and Memory and Aging Project; UPITT: University of Pittsburgh Alzheimer's Disease Research Center; UM/MASH: University of Miami Brain Endowment Bank; OHSU: Oregon Health & Science University Alzheimer's Disease Center; UM/VU/MSSM: University of Miami Hussman Institute for Human Genomics/Vanderbilt University Center for Human Genetics Research/Mount Sinai School of Medicine. (PDF) [file pgen.1004606.s039.pdf]

Table S17: Descriptive statistics of cohorts

	Case	Control	Case Sample Descriptives: Female (%)	AAO (SD)	AAD (SD)	APOE (**/*4/44)
**ACT**	63	116	43(68.3%)	84.67(5)	87.47(3.8)	0.56/0.39/0.05
**ADCs**	2301	141	1249(54.3%)	72.86(7.4)	81.42(7.5)	0.33/0.51/0.16
**TGEN2**	668	353	433(64.8%)	74.88(7.5)	82.1(7.7)	0.35/0.49/0.17
**LOAD**	424	45	276(65.1%)	74.13(7.1)	.	0.26/0.56/0.18
**MAYO**	221	209	110(49.8%)	73.63(5.4)	.	0.40/0.45/0.15
**ROSMAP**	148	80	99(66.9%)	85.12(6.6)	90.02(6.0)	0.59/0.39/0.02
**UPITT**	211	2	121(57.3%)	71.01(6.6)	.	0.37/0.51/0.12
**UM/MASH**	50	60	38(76.0%)	78.66(8.4)	84.68(7.8)	0.43/0.43/0.14
**OHSU**	12	23	8(66.7%)	89.58(6.1)	94.25(4.5)	0.58/0.42/0.00
**UMVUMSS_B**	54	1	38(70.4%)	71.81(6.8)	81.13(7.1)	0.33/0.39/0.28
**UMVUMSS_C**	21	1	12(57.1%)	71.67(6.8)	79.8(6.0)	0.33/0.48/0.19

	Case	Control	Control Sample Descriptives: Female (%)	AAE (SD)	AAD (SD)	APOE (**/*4/44)
**ACT**	63	116	67(57.8%)	82.96 (5.7)	84.1(5.4)	0.81/0.19/0.00
**ADCs**	2301	141	68(48.2%)	82.45(8.1)	82.52(8.3)	0.84/0.16/0.00
**TGEN2**	668	353	170(48.2%)	79.81(8.7)	79.81(8.7)	0.79/0.20/0.02
**LOAD**	424	45	29(64.4%)	86.67(7.4)	.	0.84/0.13/0.02
**MAYO**	221	209	79(37.8%)	71.64(5.6)	.	0.74/0.25/0.01
**ROSMAP**	148	80	52(65.0%)	84.25(6.4)	85.57(6.5)	0.94/0.05/0.01
**UPITT**	211	2			.	
**UM/MASH**	50	60	28(46.7%)	79.08(14.1)	79.08(14.1)	0.87/0.13/0.00
**OHSU**	12	23	15(65.2%)	89.52(8.1)	89.52(8.1)	0.90/0.10/0.00
**UMVUMSS_B**	54	1				
**UMVUMSS_C**	21	1				

AAO: age at onset; AAD: age at death; AAE: age at exam; SD: standard deviation. APOE: relative frequency of APOE genotypes where * represents E2 or E3.

ACT: Adult Changes in Thought Study; ADC: Alzheimer's Disease Center; TGEN: Translational Genomics Research Institute; LOAD: National Institute on Aging Late-Onset Alzheimer's Disease Family Study; MAYO: Mayo Clinic Alzheimer's Disease Research Center; ROSMAP: Religious Orders Study and Memory and Aging Project; UPITT: University of Pittsburgh Alzheimer's Disease Research Center; UM/MASH: University of Miami Brain Endowment Bank; OHSU: Oregon Health & Science University Alzheimer's Disease Center; UM/VU/MSSM: University of Miami Hussman Institute for Human Genomics/Vanderbilt University Center for Human Genetics Research/Mount Sinai School of Medicine.
