# Supplementary material for: Genome-Wide Association Meta-analysis of Neuropathologic Features of Alzheimer's Disease and Related Dementias
Source: PLoS Genet. 2014 Sep 4;10(9):e1004606. doi: 10.1371/journal.pgen.1004606 (PMC4154667; doi:10.1371/journal.pgen.1004606)
Supplement: Table S18 — Sample size and cohort inclusion by phenotype. A missing point (“.”) indicates the category had fewer than 5 observations and was not included in the analysis. A cohort must have 5 observations in two or more categories to be included in a particular analysis. Ordinal traits were coded in the order listed here. ACT: Adult Changes in Thought Study; ADC: Alzheimer's Disease Center; OHSU: Oregon Health & Science University Alzheimer's Disease Center; MAYO: Mayo Clinic Alzheimer's Disease Research Center; MBB: University of Miami Brain Endowment Bank; NIA-LOAD: National Institute on Aging Late-Onset Alzheimer's Disease Family Study; ROSMAP: Religious Orders Study and Memory and Aging Project; TGEN: Translational Genomics Research Institute; UM/VU/MSSM: University of Miami Hussman Institute for Human Genomics/Vanderbilt University Center for Human Genetics Research/Mount Sinai School of Medicine; UP: University of Pittsburgh Alzheimer's Disease Research Center. (PDF) [file pgen.1004606.s040.pdf]

Table S18: Sample size and cohort inclusion by phenotype

Cohort	Platform	Primary		Total	Complete		Total	NFT Braak (Ordinal-I)					
		Case	Control		Case	Control		0	I	II	III	IV	V
**ACT**	**Illumina 660**	63	116	179	63	116	179	13	39	63	54	34	33
**ADC**	**Illumina 660**	2301	141	2442	2299	141	2440	22	62	63	170	371	806
**OHSU**	**Illumina 300/370**	12	23	35	12	23	35	.	7	7	5	.	9
**MAYO**	**Illumina 300/370**	221	209	430	221	209	430	39	51	94	32	11	71
**MBB**	**Affy 6.0**	50	60	110	.	.	.	.	11	6	17	13	18
**NIA-LOAD**	**Illumina 610**	424	45	469	201	31	232	.	12	15	24	32	71
**ROSMAP**	**Affy 6.0**	148	80	228	148	80	228	.	22	31	75	87	99
**TGEN2**	**Affy 6.0**	668	353	1021	100	58	158	26	81	66	18	36	172
**UMVUMSS(I)**	**Illumina 610**	.	.	.	.	.	.	.	5	.	.	10	15
**UMVUMSS(II)**	**Illumina 1M**	.	.	.	.	.	.	.	.	.	.	.	.
**UP**	**Illumina 1M**	.	.	.	.	.	.	.	.	6	16	41	60
**TOTAL**	**n/a**	3887	1027	4914	3044	658	3702	100	290	351	411	635	1354

A missing point (".") indicates the category had fewer than 5 observations and was not included in the analysis. A cohort must have 5 observations in two or more categories to be included in a particular analysis. Ordinal traits were coded in the order listed here.

ACT: Adult Changes in Thought Study; ADC: Alzheimer’s Disease Center; OHSU: Oregon Health & Science University Alzheimer’s Disease Center; MAYO: Mayo Clinic Alzheimer’s Disease Research Center; MBB: University of Miami Brain Endowment Bank; NIA-LOAD: National Institute on Aging Late-Onset Alzheimer’s Disease Family Study; ROSMAP: Religious Orders Study and Memory and Aging Project; TGEN: Translational Genomics Research Institute; UM/VU/MSSM: University of Miami Hussman Institute for Human Genomics/Vanderbilt University Center for Human Genetics Research/Mount Sinai School of Medicine; UP: University of Pittsburgh Alzheimer’s Disease Research Center.

	Total	NFT Braak (Ordinal-II)				Total	Neuritic Plaque (Ordinal)				Total	Neuritic Plaque		Total
VI		0	I-II	III-IV	V-VI		None	Sparse	Moderate	Frequent		None	Any	
36	272	13	102	88	69	272	70	78	69	55	272	70	202	272
990	2484	22	125	541	1796	2484	98	73	432	1883	2486	98	2388	2486
5	33	.	14	7	14	35	19	5	.	13	37	19	19	38
139	437	39	145	43	210	437	198	20	6	213	437	198	239	437
7	72	.	17	30	25	72	45	19	27	13	104	45	59	104
123	277	.	27	56	194	277	34	12	27	134	207	34	173	207
7	321	.	53	162	106	321	119	10	62	133	324	119	205	324
182	581	26	147	54	354	581	37	33	15	93	178	37	141	178
11	41	.	8	13	26	47	.	.	.	.	.	.	.	.
.	.	.	.	10	7	17	.	.	.	.	.	.	.	.
66	189	.	9	57	126	192	.	.	12	175	187	.	.	.
1566	4707	100	647	1061	2927	4735	620	250	650	2712	4232	620	3426	4046

Lewy Body (Ordinal-I)					Total	Lewy Body		Total	Lewy Body (Ordinal-II)			Total
None	Brainstem	Transitional	Diffuse	Other		None	Any		None	Brainstem	Transitional, Diffuse, Other	
208	7	26	9	.	250	208	43	251	208	7	36	251
1525	70	189	287	165	2236	1525	711	2236	1525	70	641	2236
.	.	.	.	.	.	.	.	.	.	.	.	.
281	21	36	64	35	437	281	156	437	281	21	135	437
.	.	.	.	.	.	.	.	.	.	.	.	.
104	.	30	31	.	170	104	66	170	104	2	64	170
248	13	22	41	.	324	248	76	324	248	13	63	324
.	.	.	.	.	.	.	.	.	.	.	.	.
.	.	.	.	.	.	.	.	.	.	.	.	.
.	.	.	.	.	.	.	.	.	.	.	.	.
25	.	50	33	.	108	25	83	108	25	.	83	108
2391	111	353	465	200	3525	2391	1135	3526	2391	113	1022	3526

Vascular Brain Injury		Total	Vascular Brain Injury (Ordinal)			Total	Medial Temporal Sclerosis		Total	Amyloid Angiopathy		Total
None	Any		None	Microinfarcts	Large infarct or Lacunes		None	Any		None	Any	
115	142	257	115	27	115	257	236	17	253	197	73	270
1439	756	2195	1439	219	537	2195	1928	201	2129	767	1431	2198
14	16	30	14	.	12	26	.	.	.	22	10	32
.	.	.	.	.	.	.	.	.	.	.	.	.
106	24	130	106	9	15	130	.	.	.	122	8	130
.	.	.	.	.	.	.	.	.	.	.	.	.
.	.	.	.	34	146	180	255	67	322	5	13	18
.	.	.	.	.	.	.	.	.	.	.	.	.
.	.	.	.	.	..	.	..	.	.	.	.	.
.	.	.	.	.	.	.	.	.	.	.	.	.
98	54	152	98	22	32	152	157	25	182	21	138	159
1772	992	2764	1772	311	857	2940	2576	310	2886	1134	1673	2807
