# Supplementary material for: Genome-Wide Association Meta-analysis of Neuropathologic Features of Alzheimer's Disease and Related Dementias
Source: PLoS Genet. 2014 Sep 4;10(9):e1004606. doi: 10.1371/journal.pgen.1004606 (PMC4154667; doi:10.1371/journal.pgen.1004606)
Supplement: Text S1 — Additional Alzheimer's Disease Genetics Consortium (ADGC) members and affiliations. (DOCX) [file pgen.1004606.s041.docx]

ADGC consortium

Regina M Carney^1^, Deborah C Mash^2^, Marilyn S Albert^3^, Roger L Albin^4^;5, Liana G Apostolova^6^, Steven E Arnold^7^, Michael M Barmada^8^, Lisa L Barnes^9;10^, Thomas G Beach^11^, Eileen H Bigio^12^, Thomas D Bird^13^, Bradley F Boeve^14^, James D Bowen^15^; Adam Boxer^16^, James R Burk^17^, Nigel J Cairns^18^, Chuanhai Cao^19^, Chris S Carlson^20^, Steven L Carroll^21^, Lori B Chibnik^22;23^, Helena C Chui^24^, David G Clark^25^, Jason Corneveaux^26^, David G Cribbs^27^, Charles DeCarli^28^, Steven T DeKosky^29^, F Yesim Demirci^8^, Malcolm Dick^30^, Dennis W Dickson^31^, Ranjan Duara^32^, Nilufer Ertekin-Taner^31;33^, Kenneth B Fallon^21^, Martin R Farlow^34^, Steven Ferris35, Matthew P Frosch^36^, Douglas R Galasko^37^, Mary Ganguli^38^, Marla Gearing^39;40^, Daniel H Geschwind^41^, Bernardino Ghetti^42^, Sid Gilman^4^, Jonathan D Glass^43^, John H Growdon^44^, Ronald L Hamilton^45^, Lindy E Harrell^46^, Elizabeth Head^47^, Lawrence S Honig^48^, Christine M Hulette^49^, Bradley T Hyman^44^, Gail P Jarvik^50;51^, Gregory A Jicha^52^, Lee-Way Jin^53^, Anna Karydas^16^, John SK Kauwe^54^, Jeffrey A Kaye^55;56^, Ronald Kim^57^, Edward H Koo^37^, Neil W Kowall^58;60^, Joel H Kramer^60^, Patricia Kramer^55;61^, Frank M LaFerla^62^, James J Lah43, James B Leverenz^64^, Allan I Levey^42^, Ge Li^64^, Andrew P Lieberman^65^, Constantine G Lyketsos^66^,Wendy J Mack^67^, Daniel C Marson^46^, Frank Martiniuk^68^, Eliezer Masliah^37;69^,Wayne C McCormick^70^, SusanMMcCurry^71^, Andrew N McDavid^20^, Ann C McKee^58;59^, Marsel Mesulam^72^, Bruce L Miller^16^, Carol A Miller^73^, Joshua W Miller^74^, John C Morris^18;75^, The Alzheimer’s Disease Neuroimaging Initiative*, Jill R Murrell^41;76^, John M Olichney^77^, Vernon S Pankratz^78^, Joseph E Parisi^79;81^, Elaine Peskind^64^, Ronald C Petersen^81^, Aimee Pierce^27^, Wayne W Poon^29^, Huntington Potter^82^, Joseph F Quinn^55^, Ashok Raj^82^, Murray Raskind^64^, Eric M. Reiman^26;83;84^, Barry Reisberg^35;85^, John M Ringman^6^, Erik D Roberson^46^, Howard J Rosen^16^, Roger N Rosenberg^86^, Mary Sano^87^, Andrew J Saykin^42;88^, Julie A Schneider^9;89^, Lon S Schneider^6;90^, William W Seeley16, Amanda G Smith^82^, Joshua A Sonnen^63^, Salvatore Spina^42^, Robert A Stern^58^, Rudolph E Tanzi^44^, John Q Trojanowski^91^, Juan C Troncoso^92^, Vivianna M Van Deerlin^91^, Linda J Van Eldik^93^, Harry V Vinters^6;94^, Jean Paul Vonsattel^95^, Sandra Weintraub^72^, Kathleen A Welsh-Bohmer^96;97^, Jennifer Williamson^48^, Randall L Woltjer^98^, Chang-En Yu^70^, Robert Barber^99^.

 1. The John P. Hussman Institute for Human Genomics, University of Miami, Miami, FL, 33124, USA

 2. Department of Neurology, University of Miami, Miami, FL, 33124, USA

 3. Department of Neurology, Johns Hopkins University, Baltimore, MD, 21218, USA

 4. Department of Neurology, University of Michigan, Ann Arbor, MI, 48109, USA

 5. Geriatric Research, Education and Clinical Center (GRECC), VA Ann Arbor Healthcare System (VAAAHS), Ann Arbor, MI, 48109, USA

 6. Department of Neurology, University of California Los Angeles, Los Angeles, CA, 94607, USA

 7. Department of Psychiatry, University of Pennsylvania Perelman School of Medicine, Philadelphia, PA, 19104, USA

 8. Department of Human Genetics, University of Pittsburgh, Pittsburgh, PA, 15213, USA

 9. Department of Neurological Sciences, Rush University Medical Center, Chicago, IL, 60612, USA

10. Department of Behavioral Sciences, Rush University Medical Center, Chicago, IL, 60612, USA

11. Civil Laboratory for Neuropathology, Banner Sun Health Research Institute, Phoenix, AZ, 85351, USA

12. Department of Pathology, Northwestern University, Chicago, IL, 60208, USA

13. Department of Neurology, University of Washington, Seattle, WA, 98195, USA

14. Department of Neurology, Mayo Clinic, Rochester, MN, 55902, USA

15. Swedish Medical Center, Seattle, WA, 98195, USA

16. Department of Neurology, University of California San Francisco, San Francisco, CA, 94122, USA

17. Department of Medicine, Duke University, Durham, NC, 27710, USA

18. Department of Pathology and Immunology, Washington University, St. Louis, MO, 63130, USA

19. USF Health Byrd Alzheimer’s Institute, University of South Florida, Tampa, CA, 33620, USA

20. Fred Hutchinson Cancer Research Center, Seattle, WA, 98195, USA

21. Department of Pathology, University of Alabama at Birmingham, Birmingham, AL, 35294, USA

22. Program in Translational NeuroPsychiatric Genomics, Institute for the Neurosciences, Department of Neurology & Psychiatry, Bringham and Women’s Hospital, Harvard Medical School, Boston, MA, 02215, USA

23. Program in Medical and Population Genetics, Broad Institute, Boston, MA, 02215, USA

24. Department of Neurology, University of Southern California, Los Angeles, CA, 94607, USA

25. Department of Neurology, University of Alabama at Birmingham, Birmingham, AL, 35294, USA

26. Neurogenomics Division, Translational Genomics Research Institute, Phoenix, AZ, 85004, USA

27. Department of Neurology, University of California Irvine, Irvine, CA, 92617, USA

28. Department of Neurology, University of California Davis, Sacramento, CA, 95616, USA

29. University of Virginia School of Medicine, Charlottesville, VA, 22903, USA

30. Institute for Memory Impairments and Neurological Disorders, University of California Irvine, Irvine, CA, 92617, USA

31. Department of Neuroscience, Mayo Clinic, Jacksonville, FL, 55902, USA

32. Wien Center for Alzheimer’s Disease and Memory Disorders, Mount Sinai Medical Center, Miami Beach, FL, 10029, USA

33. Department of Neurology, Mayo Clinic, Jacksonville, FL, 55902, USA

34. Department of Neurology, Indiana University, Indianapolis, IN, 46202, USA

35. Department of Psychiatry, New York University, New York, NY, 10027, USA

36. C.S. Kubik Laboratory for Neuropathology, Massachusetts General Hospital, Charlestown, MA, 02114, USA

37. Department of Neurosciences, University of California San Diego, La Jolla, CA, 92093, USA

38. Department of Psychiatry, University of Pittsburgh, Pittsburgh, PA, 15213, USA

39. Department of Pathology and Laboratory Medicine, Emory University, Atlanta, GA, 30329, USA

40. Emory Alzheimer’s Disease Center, Emory University, Atlanta, GA, 30329, USA

41. Neurogenetics Program, University of California Los Angeles, Los Angeles, CA, 94607, USA

42. Department of Pathology and Laboratory Medicine, Indiana University, Indianapolis, IN, 46202, USA

43. Department of Neurology, Emory University, Atlanta, GA, 30329, USA

44. Department of Neurology, Massachusetts General Hospital/Harvard Medical School, Boston, MA, 02215, USA

45. Department of Pathology (Neuropathology), University of Pittsburgh, Pittsburgh, PA, 15213, USA

46. Department of Neurology, University of Alabama at Birmingham, Birmingham, AL, 35294, USA

47. Sanders-Brown Center on Aging, Department of Molecular and Biomedical Pharmacology, University of Kentucky, Lexington, KY, 40506, USA

48. Taub Institute on Alzheimer’s Disease and the Aging Brain, Department of Neurology, Columbia University, New York, NY, 10027, USA

49. Department of Pathology, Duke University, Durham, NC, 27710, USA

50. Department of Genome Sciences, University of Washington, Seattle, WA, 98195, USA

51. Department of Medicine (Medical Genetics), University of Washington, Seattle, WA, 98195, USA

52. Sanders-Brown Center on Aging, Department Neurology, University of Kentucky, Lexington, KY, 40506, USA

53. Department of Pathology and Laboratory Medicine, University of California Davis, Sacramento, CA, 95616, USA

54. Department of Biology, Brigham Young University, Provo, UT 02215, USA

55. Department of Neurology, Oregon Health & Science University, Portland, OR, 97239, USA

56. Department of Neurology, Portland Veterans A_airs Medical Center, Portland, OR, 97239, USA

57. Department of Pathology and Laboratory Medicine, University of California Irvine, Irvine, CA, 92617, USA

58. Department of Neurology, Boston University School of Medicine, Boston, MA, 02215, USA

59. Department of Pathology, Boston University School of Medicine, Boston, MA, 02215, USA

60. Department of Neuropsychology, University of California San Francisco, San Francisco, CA, 94122, USA

61. Department of Molecular & Medical Genetics, Oregon Health & Science University, Portland, OR, 97239, USA

62. Department of Neurobiology and Behavior, University of California Irvine, Irvine, CA, 92617, USA

63. Department of Pathology, University of Washington, Seattle, WA, 98195, USA

64. Department of Psychiatry and Behavioral Sciences, University of Washington, Seattle, WA, 98195, USA

65. Department of Pathology, University of Michigan, Ann Arbor, MI, 48109, USA

66. Department of Psychiatry, Johns Hopkins University, Baltimore, MD, 21218, USA

67. Department of Preventive Medicine, University of Southern California, Los Angeles, CA, 94607, USA

68. Department of Medicine - Pulmonary, New York University, New York, NY, 10027, USA

69. Department of Pathology, University of California San Diego, La Jolla, CA, 92093, USA

70. Department of Medicine, University of Washington, Seattle, WA, 98195, USA

71. School of Nursing Northwest Research Group on Aging, University of Washington, Seattle, WA, 98195, USA

72. Cognitive Neurology and Alzheimer’s Disease Center, Northwestern University, Chicago, IL, 60208, USA

73. Department of Pathology, University of Southern California, Los Angeles, CA, 94607, USA

74. Department of Pathology and Laboratory Medicine, University of California Davis, Sacramento, CA, 95616, USA

75. Department of Neurology, Washington University, St. Louis, MO, 98101, USA

76. Department of Medical and Molecular Genetics, Indiana University, Indianapolis, IN, 46202, USA

77. Department of Neurology, University of California Davis, Sacramento, CA, 95616, USA

78. Department of Biostatistics, Mayo Clinic, Rochester, MN, 55902, USA

79. Department of Anatomic Pathology, Mayo Clinic, Mayo Clinic, Rochester, MN, 55902, USA

80. Department of Laboratory Medicine and Pathology, Mayo Clinic, Rochester, MN, 55902, USA

81. Department of Neurology, Mayo Clinic, Rochester, MN, 55902, USA

82. USF Health Byrd Alzheimer’s Institute, University of South Florida, Tampa, FL, 33613, USA

83. Arizona Alzheimer’s Consortium, Department of Psychiatry, University of Arizona, Phoenix, AZ, 85004, USA

84. Banner Alzheimer’s Institute, Phoenix, AZ, 85004, USA

85. Alzheimer’s Disease Center, New York University, New York, NY, 10027, USA

86. Department of Neurology, University of Texas Southwestern, Dallas, TX, 75390, USA

87. Department of Psychiatry, Mount Sinai School of Medicine, New York, NY, 10027, USA

88. Department of Radiology and Imaging Sciences, Indiana University, Indianapolis, IN, 46202, USA

89. Department of Pathology (Neuropathology), Rush University Medical Center,Chicago, IL, 60208, USA

90. Department of Psychiatry, University of Southern California, Los Angeles, CA, 94607, USA

91. Department of Pathology and Laboratory Medicine, University of Pennsylvania, Perelman School of Medicine, Philadelphia, PA, 19104, USA

92. Department of Pathology, Johns Hopkins University, Baltimore, MD, 21218, USA

93. Sanders-Brown Center on Aging, Department of Anatomy and Neurobiology, University of Kentucky, Lexington, KY, 40506, USA

94. Department of Pathology & Laboratory Medicine, University of California Los Angeles, Los Angeles, CA, 94607, USA

95. Taub Institute on Alzheimer’s Disease and the Aging Brain, Department of Pathology, Columbia University, New York, NY, 10027, USA

96. Department of Medicine, Duke University, Durham, NC, 27710, USA

97. Department of Psychiatry & Behavioral Sciences, Duke University, Durham, NC, 27710, USA

98. Department of Pathology, Oregon Health & Science University, Portland, OR, 97239, USA

99. Department of Pharmacology and Neuroscience, University of North Texas Health Science Center, FortWorth, TX, 76102, USA
