# Supplementary material for: Correction: Genome-Wide Association Meta-analysis of Neuropathologic Features of Alzheimer’s Disease and Related Dementias
Source: PLoS Genet. 2014 Nov 12;10(11):e1004867. doi: 10.1371/journal.pgen.1004867 (PMC4229109; doi:10.1371/journal.pgen.1004867)
Supplement: Text S1 — Additional Alzheimer's Disease Genetics Consortium (ADGC) members and affiliations (updated). (DOCX) [file pgen.1004867.s001.docx]

Alzheimer’s Disease Genetics Consortium Members

Marilyn S. Albert^1^, Roger L. Albin^2-4^, Liana G. Apostolova^5^, Steven E. Arnold^6^, Sanjay Asthana^7-9^, Craig S. Atwood^7,9^, Clinton T. Baldwin^10^, Michael M. Barmada^11^, Lisa L. Barnes^12,13^, Thomas G. Beach^14^, James T. Becker^15^, Duane Beekly^16^, Eileen H. Bigio^17,18^, Thomas D. Bird^19,20^, Deborah Blacker^21,22^, Bradley F. Boeve^23^, James D. Bowen^24^, Adam Boxer^25^, James R. Burke^26^, Nigel J. Cairns^27^, Laura B. Cantwell^28^, Chuanhai Cao^29^, Chris S. Carlson^30^, Cynthia M. Carlsson^8^, Regina M. Carney^31^, Minerva M. Carrasquillo^32^, Steven L. Carroll^33^, Helena C. Chui^34^, David G. Clark^35^, David H. Cribbs^36^, Elizabeth A. Crocco^31^, Carlos Cruchaga^37^, Charles DeCarli^38^, F. Yesim Demirci^11^, Malcolm Dick^39^, Ranjan Duara^40^, Nilufer Ertekin-Taner^32,41^, Kelley M. Faber^42^, Kenneth B. Fallon^33^, Martin R. Farlow^43^, Steven Ferris^44^, Tatiana M. Foroud^42^, Douglas R. Galasko^45^, Marla Gearing^46,47^, Daniel H. Geschwind^48^, Jonathan D. Glass^49^, Alison M. Goate^37^, Neill R. Graff-Radford^32,41^, Robert C. Green^50^, John H. Growdon^51^, Hakon Hakonarson^52^, Ronald L. Hamilton^53^, Lindy E. Harrell^35^, Elizabeth Head^54^, Lawrence S. Honig^55^, Christine M. Hulette^56^, Gail P. Jarvik^57,58^, Gregory A. Jicha^59^, Lee-Way Jin^60^, Gyungah Jun^10,61,62^, Anna Karydas^25^, John S.K. Kauwe^63^, Jeffrey A. Kaye^64,65^, Ronald Kim^66^, Neil W. Kowall^67,68^, Joel H. Kramer^69^, Brian W. Kunkle^70^, Frank M. LaFerla^71^, James J. Lah^49^, James B. Leverenz^72^, Allan I. Levey^49^, Ge Li^73^, Andrew P. Lieberman^74^, Chiao-Feng Lin^28^, Oscar L. Lopez^75^, Kathryn L. Lunetta^61^, Constantine G. Lyketsos^76^, Wendy J. Mack^77^, Daniel C. Marson^35^, Frank Martiniuk^78^, Eliezer Masliah^45,79^, Wayne C. McCormick^80^, Susan M. McCurry^81^, Andrew N. McDavid^30^, Ann C. McKee^67,68^, Marsel Mesulam^18,82^, Bruce L. Miller^25^, Carol A. Miller^83^, Joshua W. Miller^60^, John C. Morris^27,84^, Shubhabrata Mukherjee^80^, Jill R. Murrell^42,85^, John M. Olichney^38^, Vernon S. Pankratz^86^, Joseph E. Parisi^87^, Amanda Partch^28^, Henry L. Paulson^88^, William Perry^70^, Elaine Peskind^73^, Ronald C. Petersen^23^, Aimee Pierce^36^, Wayne W. Poon^39^, Huntington Potter^89^, Joseph F. Quinn^64^, Ashok Raj^29^, Murray Raskind^73^, Barry Reisberg^44,90^, Christiane Reitz^55,91,92^, John M. Ringman^5^, Erik D. Roberson^35^, Ekaterina Rogaeva^93^, Howard J. Rosen^25^, Roger N. Rosenberg^94^, Mark A. Sager^8^, Mary Sano^95^, Andrew J. Saykin^42,96^, Lon S. Schneider^34,97^, William W. Seeley^25^, Amanda G. Smith^29^, Joshua A. Sonnen^98^, Salvatore Spina^85^, Peter St George-Hyslop^93,99^, Robert A. Stern^67^, Rudolph E. Tanzi^51^, Tricia A. Thornton-Wells^100^, Juan C. Troncoso^101^, Debby W. Tsuang^20,73^, Otto Valladares^28^, Vivianna M. Van Deerlin^28^, Linda J. Van Eldik^102^, Badri N. Vardarajan^55,91,92^, Harry V. Vinters^5,103^, Li-San Wang^28^, Sandra Weintraub^18,104^, Kathleen A. Welsh-Bohmer^26,105^, Jennifer Williamson^55^, Sarah Wishnek^70^, Randall L. Woltjer^106^, Clinton B. Wright^107^, Chang-En Yu^80^, Lei Yu^12^

^1^Department of Neurology, Johns Hopkins University, Baltimore, Maryland, ^2^Department of Neurology, University of Michigan, Ann Arbor, Michigan, ^3^Geriatric Research, Education and Clinical Center (GRECC), VA Ann Arbor Healthcare System (VAAAHS), Ann Arbor, Michigan, ^4^Michigan Alzheimer Disease Center, Ann Arbor, Michigan, ^5^Department of Neurology, University of California Los Angeles, Los Angeles, California, ^6^Department of Psychiatry, University of Pennsylvania Perelman School of Medicine, Philadelphia, Pennsylvania, ^7^Geriatric Research, Education and Clinical Center (GRECC), University of Wisconsin, Madison, Wisconsin, ^8^Department of Medicine, University of Wisconsin, Madison, Wisconsin, ^9^Wisconsin Alzheimer's Institute, Madison, Wisconsin, ^10^Department of Medicine (Genetics Program), Boston University, Boston, Massachusetts, ^11^Department of Human Genetics, University of Pittsburgh, Pittsburgh, Pennsylvania, ^12^Department of Neurological Sciences, Rush University Medical Center, Chicago, Illinois, ^13^Department of Behavioral Sciences, Rush University Medical Center, Chicago, Illinois, ^14^Civin Laboratory for Neuropathology, Banner Sun Health Research Institute, Phoenix, Arizona, ^15^Departments of Psychiatry, Neurology, and Psychology, University of Pittsburgh School of Medicine, Pittsburgh, Pennsylvania, ^16^National Alzheimer's Coordinating Center, University of Washington, Seattle, Washington, ^17^Department of Pathology, Northwestern University Feinberg School of Medicine, Chicago, Illinois, ^18^Cognitive Neurology and Alzheimer's Disease Center, Northwestern University Feinberg School of Medicine, Chicago, Illinois, ^19^Department of Neurology, University of Washington, Seattle, Washington, ^20^VA Puget Sound Health Care System/GRECC, Seattle, Washington, ^21^Department of Epidemiology, Harvard School of Public Health, Boston, Massachusetts, ^22^Department of Psychiatry, Massachusetts General Hospital/Harvard Medical School, Boston, Massachusetts, ^23^Department of Neurology, Mayo Clinic, Rochester, Minnesota, ^24^Swedish Medical Center, Seattle, Washington, ^25^Department of Neurology, University of California San Francisco, San Francisco, California, ^26^Department of Medicine, Duke University, Durham, North Carolina, ^27^Department of Pathology and Immunology, Washington University, St. Louis, Missouri, ^28^Department of Pathology and Laboratory Medicine, University of Pennsylvania Perelman School of Medicine, Philadelphia, Pennsylvania, ^29^USF Health Byrd Alzheimer's Institute, University of South Florida, Tampa, Florida, ^30^Fred Hutchinson Cancer Research Center, Seattle, Washington, ^31^Department of Psychiatry and Behavioral Sciences, Miller School of Medicine, University of Miami, Miami, Florida, ^32^Department of Neuroscience, Mayo Clinic, Jacksonville, Florida, ^33^Department of Pathology, University of Alabama at Birmingham, Birmingham, Alabama, ^34^Department of Neurology, University of Southern California, Los Angeles, California, ^35^Department of Neurology, University of Alabama at Birmingham, Birmingham, Alabama, ^36^Department of Neurology, University of California Irvine, Irvine, California, ^37^Department of Psychiatry and Hope Center Program on Protein Aggregation and Neurodegeneration, Washington University School of Medicine, St. Louis, Missouri, ^38^Department of Neurology, University of California Davis, Sacramento, California, ^39^Institute for Memory Impairments and Neurological Disorders, University of California Irvine, Irvine, California, ^40^Wien Center for Alzheimer's Disease and Memory Disorders, Mount Sinai Medical Center, Miami Beach, Florida, ^41^Department of Neurology, Mayo Clinic, Jacksonville, Florida, ^42^Department of Medical and Molecular Genetics, Indiana University, Indianapolis, Indiana, ^43^Department of Neurology, Indiana University, Indianapolis, Indiana, ^44^Department of Psychiatry, New York University, New York, New York, ^45^Department of Neurosciences, University of California San Diego, La Jolla, California, ^46^Department of Pathology and Laboratory Medicine, Emory University, Atlanta, Georgia, ^47^Emory Alzheimer's Disease Center, Emory University, Atlanta, Georgia, ^48^Neurogenetics Program, University of California Los Angeles, Los Angeles, California, ^49^Department of Neurology, Emory University, Atlanta, Georgia, ^50^Division of Genetics, Department of Medicine and Partners Center for Personalized Genetic Medicine, Brigham and Women's Hospital and Harvard Medical School, Boston, Massachusetts, ^51^Department of Neurology, Massachusetts General Hospital/Harvard Medical School, Boston, Massachusetts, ^52^Center for Applied Genomics, Children's Hospital of Philadelphia, Philadelphia, Pennsylvania, ^53^Department of Pathology (Neuropathology), University of Pittsburgh, Pittsburgh, Pennsylvania, ^54^Sanders-Brown Center on Aging, Department of Molecular and Biomedical Pharmacology, University of Kentucky, Lexington, Kentucky, ^55^Taub Institute on Alzheimer's Disease and the Aging Brain, Department of Neurology, Columbia University, New York, New York, ^56^Department of Pathology, Duke University, Durham, North Carolina, ^57^Department of Genome Sciences, University of Washington, Seattle, Washington, ^58^Department of Medicine (Medical Genetics), University of Washington, Seattle, Washington, ^59^Sanders-Brown Center on Aging, Department Neurology, University of Kentucky, Lexington, Kentucky, ^60^Department of Pathology and Laboratory Medicine, University of California Davis, Sacramento, California, ^61^Department of Biostatistics, Boston University, Boston, Massachusetts, ^62^Department of Ophthalmology, Boston University, Boston, Massachusetts, ^63^Department of Biology, Brigham Young University, Provo, Utah, ^64^Department of Neurology, Oregon Health & Science University, Portland, Oregon, ^65^Department of Neurology, Portland Veterans Affairs Medical Center, Portland, Oregon, ^66^Department of Pathology and Laboratory Medicine, University of California Irvine, Irvine, California, ^67^Department of Neurology, Boston University, Boston, Massachusetts, ^68^Department of Pathology, Boston University, Boston, Massachusetts, ^69^Department of Neuropsychology, University of California San Francisco, San Francisco, California, ^70^The John P. Hussman Institute for Human Genomics, University of Miami, Miami, Florida, ^71^Department of Neurobiology and Behavior, University of California Irvine, Irvine, California, ^72^Cleveland Clinic Lou Ruvo Center for Brain Health, Cleveland Clinic, Cleveland, Ohio, ^73^Department of Psychiatry and Behavioral Sciences, University of Washington School of Medicine, Seattle, Washington, ^74^Department of Pathology, University of Michigan, Ann Arbor, Michigan, ^75^University of Pittsburgh Alheimer's Disease Research Center, Pittsburgh, Pennsylvania, ^76^Department of Psychiatry, Johns Hopkins University, Baltimore, Maryland, ^77^Department of Preventive Medicine, University of Southern California, Los Angeles, California, ^78^Department of Medicine - Pulmonary, New York University, New York, New York, ^79^Department of Pathology, University of California San Diego, La Jolla, California, ^80^Department of Medicine, University of Washington, Seattle, Washington, ^81^School of Nursing Northwest Research Group on Aging, University of Washington, Seattle, Washington, ^82^Department of Neurology, Northwestern University Feinberg School of Medicine, Chicago, Illinois, ^83^Department of Pathology, University of Southern California, Los Angeles, California, ^84^Department of Neurology, Washington University, St. Louis, Missouri, ^85^Department of Pathology and Laboratory Medicine, Indiana University, Indianapolis, Indiana, ^86^Department of Biostatistics, Mayo Clinic, Rochester, Minnesota, ^87^Department of Laboratory Medicine and Pathology, Mayo Clinic, Rochester, Minnesota, ^88^Michigan Alzheimer's Disease Center, Department of Neurology, University of Michigan, Ann Arbor, Michigan, ^89^Department of Neurology, University of Colorado School of Medicine, Aurora, Colorado, ^90^Alzheimer's Disease Center, New York University, New York, New York, ^91^Gertrude H. Sergievsky Center, Columbia University, New York, New York, ^92^Department of Neurology, Columbia University, New York, New York, ^93^Tanz Centre for Research in Neurodegenerative Disease, University of Toronto, Toronto, Ontario, ^94^Department of Neurology, University of Texas Southwestern, Dallas, Texas, ^95^Department of Psychiatry, Mount Sinai School of Medicine, New York, New York, ^96^Department of Radiology and Imaging Sciences, Indiana University, Indianapolis, Indiana, ^97^Department of Psychiatry, University of Southern California, Los Angeles, California, ^98^Department of Pathology, University of Washington, Seattle, Washington, ^99^Cambridge Institute for Medical Research and Department of Clinical Neurosciences, University of Cambridge, Cambridge, UK ^100^Center for Human Genetics and Research, Department of Molecular Physiology and Biophysics, Vanderbilt University, Nashville, Tennessee, ^101^Department of Pathology, Johns Hopkins University, Baltimore, Maryland, ^102^Sanders-Brown Center on Aging, Department of Anatomy and Neurobiology, University of Kentucky, Lexington, Kentucky, ^103^Department of Pathology & Laboratory Medicine, University of California Los Angeles, Los Angeles, California, ^104^Department of Psychiatry, Northwestern University Feinberg School of Medicine, Chicago, Illinois, ^105^Department of Psychiatry & Behavioral Sciences, Duke University, Durham, North Carolina, ^106^Department of Pathology, Oregon Health & Science University, Portland, Oregon, ^107^Evelyn F. McKnight Brain Institute, Department of Neurology, Miller School of Medicine, University of Miami, Miami, Florida
